# Ice nucleation by kaolinite: Morphology shapes the size dependence of ice nucleation active sites on dust particles

**DOI:** 10.1126/sciadv.aeg1206

**Published:** 2026-10-14

**Authors:** Yuhang Song, Claudia Marcolli, Jie Chen, Ulrike Lohmann

**Affiliations:** Institute for Atmospheric and Climate Science, Swiss Federal Institute of Technology (ETH) Zürich, 8092 Zürich, Switzerland.

## Abstract

Mineral dusts are the most important type of ice-nucleating particles and play a key role in cloud formation and climate. Their activity is commonly assumed to scale with particle surface area, yet whether this assumption holds across particle sizes remains unverified. Here, we quantify size-dependent ice nucleation activity using compositionally pure, size-segregated kaolinite (KGa-1b). Ice nucleation active site (INAS) densities were determined for size classes from submicron to 48 μm, revealing two distinct ice-nucleation regimes. At low temperatures (∼240 K), INAS density per surface area is independent of particle size. In contrast, at warm temperatures (∼260 K), particles of 6 to 12 μm exhibit the highest INAS densities, up to two orders of magnitude greater than smaller particles and nonsize-selected bulk samples. Linking these results to particle morphology indicates that well-aligned, multilayered platelet edges provide the continuous hydroxylated surfaces required for efficient ice nucleation. Our findings demonstrate that morphology can modulate particle size–dependent ice nucleation, highlighting limitations of simple surface area–based parameterizations.

## INTRODUCTION

Clouds play a crucial role in Earth’s climate system by influencing radiation, precipitation, and hydrological cycles (*1*–*4*). Ice crystals within clouds impact cloud lifetime and radiative properties (*5*, *6*), making it essential to understand the mechanisms of ice nucleation (IN) for accurate climate modeling (*7*, *8*).

In the absence of suitable particles, homogeneous freezing occurs at around ∼236 K. At higher temperatures (236 K < *T* < 273 K), ice nucleating particles (INPs) can trigger heterogeneous IN, leading to mixed-phase clouds. Immersion freezing, where an INP is suspended in a supercooled cloud droplet, is considered the dominant pathway for liquid cloud glaciation (*9*, *10*). Mineral dusts sourced from arid regions around the globe and ranging from submicron to tenth of microns in particle size (*11*) are the most prominent type of INPs due to their abundance and IN efficiency, particularly below 250 K (*12*, *13*).

The IN activity of mineral dust is influenced by both composition (*13*–*15*) and particle size. Immersion freezing is typically assumed to scale with particle surface area: within classical nucleation theory (CNT), heterogeneous nucleation rates are proportional to the surface area of the substrate that promotes ice formation (*16*–*18*). On the other hand, the investigation of IN on individual INPs revealed specific surface locations, so-called active sites that nucleate ice at higher temperatures than the surrounding areas (*19*). The concept of ice nucleation active site (INAS) density likewise assumes proportionality to surface area (*14*, *20*). Both perspectives therefore imply that smaller particles are less efficient INPs than larger ones and that IN activity scales with surface area. However, empirical tests of this assumption remain limited. For submicron kaolinite (*21*) and feldspar (*22*), IN activity was shown to increase with particle size, but surface area scaling has not been established. Studies of atmospheric dust samples showed increasing INAS densities from submicron to ∼10-μm particles, although these trends may rather reflect size-dependent variations in composition than intrinsic nucleation efficiency of a single type of INP (*23*–*25*). An intercomparison study found that IN activity of illite scales with surface area across 236 to 262 K and over roughly 10 orders of magnitude in INAS density, albeit with substantial intermethod variability (*26*). Here, we quantify how particle size influences ice-nucleating activity across a broad temperature and surface area range using a size-segregated mineral dust sample.

To isolate size effects within one single mineral, we used KGa-1b kaolinite from the Clay Minerals Society (CMS), a mineralogically almost pure (∼96%) reference sample (*27*, *28*) widely used in IN research (*17*, *21*, *29*–*33*). Kaolinite particles are layered aluminosilicates made of silica and alumina sheets, which are stacked to form platy particles with silica and alumina basal surfaces and hydroxylated edges (*34*, *35*). These platelets may further aggregate to form larger structures (*36*, *37*). Both the silica surfaces and the edges have been discussed as potential locations of IN (*29*, *30*, *38*, *39*), with electron microscopy observations indicating preferential ice formation at the hydroxylated platelet edges (*40*). To investigate the size dependence of INAS densities, we separated the aerosolized kaolinite particles with an impactor into different size fractions and conducted immersion freezing experiments from 236 to 273 K using two different drop freezing assays to cover IN over this large temperature range.

## RESULTS

### Freezing experiments

KGa-1b kaolinite particles were aerosolized to size-segregate them in an impactor, from which the seven size stages with the highest collected masses (table S2) were selected for freezing experiments (see Materials and Methods). These were the stages with cutoff points D50 = 0.32, 0.56, 1.0, 1.8, 3.2, 5.6, and 10 μm. Particle size distributions for these stages were determined from scanning electron microscopy (SEM) measurements, which revealed that small particles were dominant in number on all stages but that only the stages with larger cutoffs contained larger particles (fig. S2). Although full size segregation with the impactor was not achieved—likely due to the irregular particle morphologies—it was partially successful, as indicated by the increase in mass-equivalent diameter ($D_{\text{me}}$) from 1.87 μm for D50 = 0.32 to 11.43 μm for D50 = 10 μm.

Immersion freezing of the particles collected from these stages was investigated using two complementary droplet freezing methods. With 50-μl droplets, the Droplet Ice Nuclei Counter Zürich (DRINCZ) is suited for detecting freezing events down to ∼250 K where freezing on omnipresent IN-active impurities becomes dominant. In contrast, the Microfluidic Ice Nuclei Counter Zürich (MINCZ) uses 75-μm-diameter (∼220 pl) droplets, which are ∼200,000 times smaller in volume. This substantial reduction in droplet size minimizes the influence of random impurities, enabling the investigation of INPs down to the temperature where homogeneous freezing sets in (∼236 K for this droplet size).

DRINCZ experiments (Materials and Methods) performed for the concentrations of 0.05 and 0.1 weight % (wt %) revealed a clear particle size dependence of frozen fractions, with the trend of larger cutoff sizes (D50 = 3.2, 5.6, and 10 μm) exhibiting higher frozen fractions than smaller ones (D50 = 0.32, 0.56, and 1.0 μm) and freezing onsets reaching up to 268 K (Fig. 1 and fig. S3). Using Brunauer-Emmett-Teller (BET) gas adsorption specific surface areas (SSAs; see Materials and Methods), frozen fractions were converted to INAS densities. The resulting stage-derived INAS densities confirm the trend observed for frozen fractions, namely, higher IN activities for the stages with larger cutoffs (fig. S4).

**Fig. 1.**
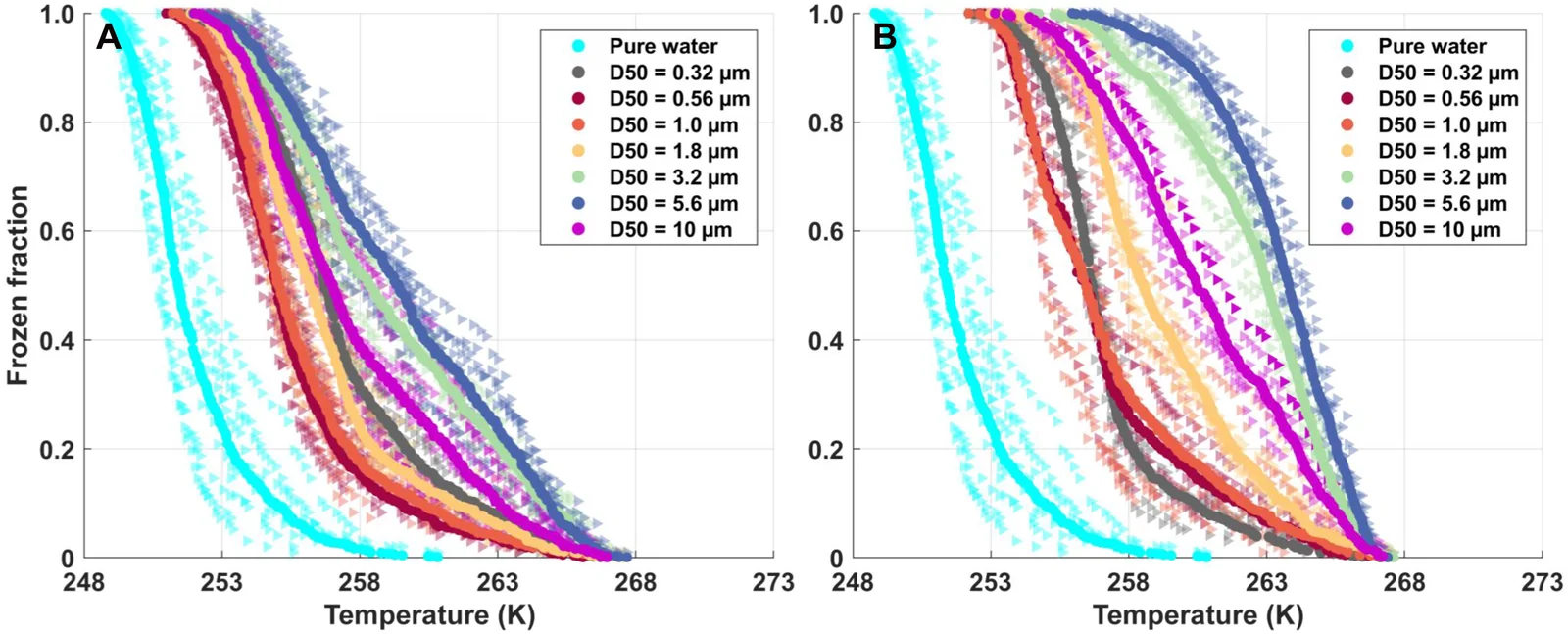
Freezing behavior across impactor stages in DRINCZ droplets. Frozen fractions of 50-μl DRINCZ droplets containing (**A**) 0.05 wt % and (**B**) 0.1 wt % kaolinite KGa-1b from the seven impactor stages (D50 = 0.32, 0.56, 1.0, 1.8, 3.2, 5.6, and 10 μm) during cooling at a rate of 1 K min^−1^. Dark solid circles denote the average frozen fraction, and the semitransparent triangles indicate individual experiments.

MINCZ experiments were performed at 0.05, 0.1, and 0.5 wt %, with additional 1.0 wt % measurements for D50 = 1.0 and 1.8 μm (Fig. 2). Frozen fraction curves show again a temperature dependence; however, it is reversed compared with the DRINCZ experiments. Yet, this result should be taken with caution because the small droplet volume in MINCZ experiments (∼220 pl) may introduce variability in particle number per droplet, potentially underrepresenting larger particles. Converting the frozen fractions to INAS densities reveals that experiments at different suspension concentrations do not yield consistent stage-derived INAS densities, but that for some cutoff sizes, an increase in concentration yields a disproportionate increase in INAS densities (fig. S5). This variance likely arises because a fraction of low-concentration droplets contains few or no particles, resulting in uneven particle distributions among droplets.

**Fig. 2.**
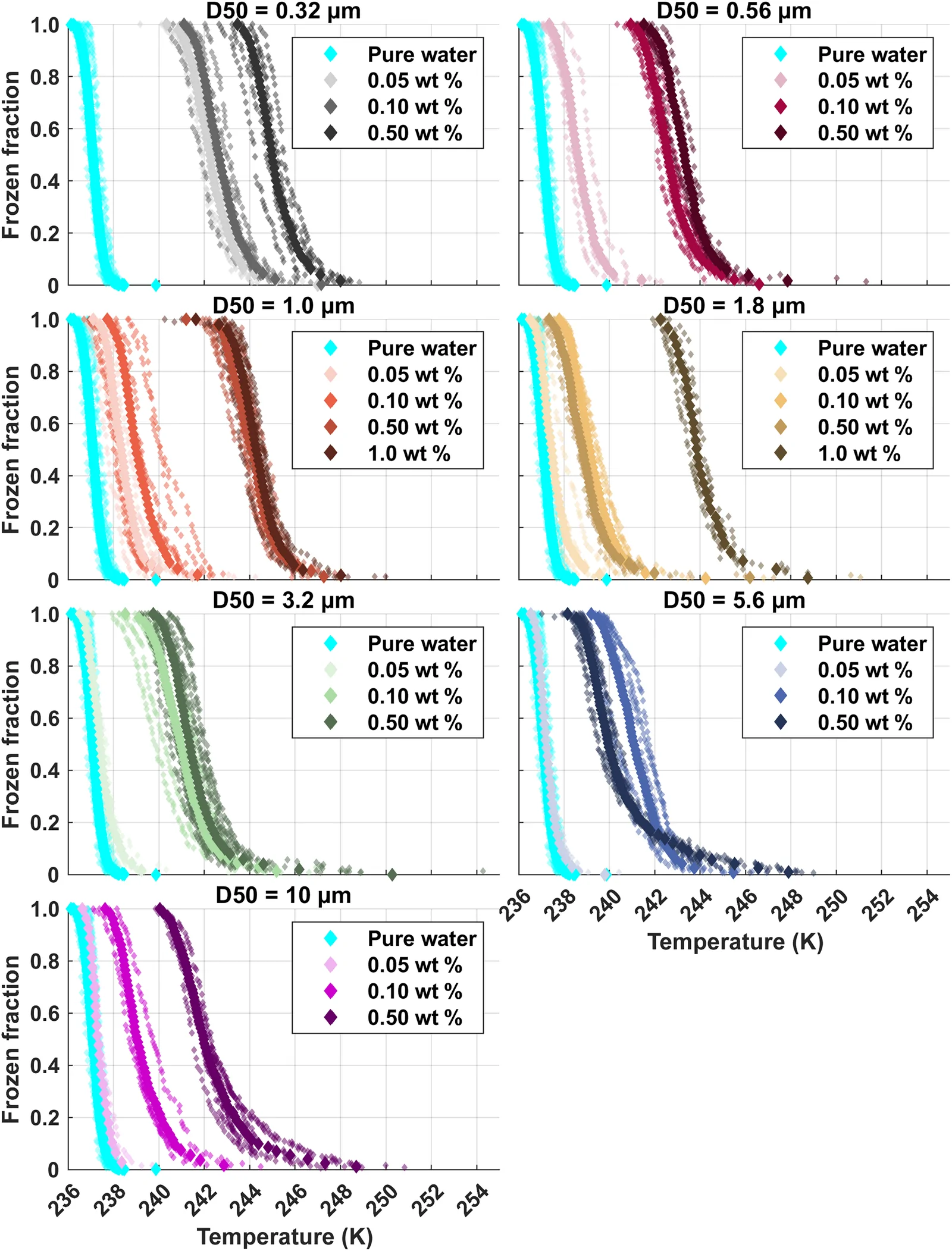
Freezing behavior across impactor stages in MINCZ droplets. Frozen fractions of 75-μm-diameter MINCZ droplets containing 0.05, 0.1, 0.5, and 1.0 wt % KGa-1b for the investigated impactor stages. Frozen fractions for 1.0 wt % have only been measured for D50 = 1.0 and 1.8 μm. Solid diamonds denote the average frozen fraction, whereas semitransparent diamonds indicate individual experiments.

To assess whether the full range of particle sizes is represented in each sample aliquot, we estimated the average number of particles per droplet based on the size distribution established in fig. S2, along with the droplet volumes and particle concentrations used (section S3). These calculations revealed that the 50-μl droplets used in DRINCZ are large enough to accommodate the full range of particle sizes detected on the investigated Micro-Orifice Uniform Deposit Impactor (MOUDI) stages, resulting in a relatively uniform particle size distribution across the wells of the DRINCZ plate (tables S4 to S10). Yet, the MINCZ droplets are too small to comprise the full size range, including the largest particles.

In a next step, we used Monte Carlo simulations (section S3 and fig. S7) to investigate the number and size distribution of particles in populations of MINCZ droplets. These simulations reveal that only for D50 = 0.32 μm at 0.5 wt % all particle sizes are present and evenly distributed across all 150 simulated droplets (figs. S8 to S14). For larger cutoffs or lower suspension concentrations, particle distributions become increasingly uneven: Thus, only some droplets contain the largest particles, while others are filled only with a few smaller ones. Overall, the number of particles per droplet decreases with increasing D50, and for D50 = 10 μm at 0.05 wt %, over half of the droplets were even predicted empty.

Comparing these simulations with the measured MINCZ experiments indicates that the overlap of the freezing curve for D50 = 10 μm at 0.05 wt % with that of pure water likely arises from empty droplets that freeze homogeneously. Yet, MINCZ experiments with D50 = 1.8- to 5.6-μm particles, suspended at 0.05 wt %, froze close to the homogeneous freezing curve, although the Monte Carlo simulations predict the presence of particles. This discrepancy possibly results from deviations from the nominal droplet concentrations caused by additional particle losses during suspension handling and droplet generation, such as sedimentation and adhesion of particles to the tubing walls. It could also reflect inaccuracies in the SEM-derived size distributions, leading to droplets being predicted as occupied when they were in fact empty. Alternatively, droplets containing kaolinite particles may still have frozen homogeneously if these particles lacked active sites capable of inducing freezing above the homogeneous freezing temperature of the MINCZ droplets.

Stage-derived frozen fractions and INAS densities reflect the overall IN activity of particles collected within a given impactor stage. However, the broad particle-size range within each stage, together with the variable number distribution of differently sized particles among droplets, makes it difficult to directly quantify the IN activity of individual particle sizes. To address this limitation, we developed an integrated experimental and simulation framework that combines size-segregated aerosol sampling, droplet freezing experiments, SEM-derived particle size distributions, and iterative optimization to derive size-resolved INAS densities (fig. S15). This framework enables a more direct and quantitative evaluation of the size dependence of IN activity.

### Derivation of size-resolved INAS densities and IN-active particle fraction

To elucidate how IN depends on particle size, we derived INAS densities and IN-active particle fractions of kaolinite by simulating the measured DRINCZ and MINCZ freezing curves in an iterative procedure by (i) guessing INAS densities, (ii) converting INAS densities to temperature-dependent IN-active particle fractions, which were then used to (iii) simulate the MINCZ and DRINCZ frozen fractions based on the simulated particles present in each droplet. The stage-derived INAS densities were used to identify datasets in which the actual particle loading in droplets deviated substantially from nominal concentration (section S2 and fig. S5), and these nonrepresentative datasets were excluded from the subsequent simulation optimization (table S15). In addition, the stage-derived INAS densities provided the initial guess for size-resolved INAS densities, $n_{s}\left(T\right)$, in the simulation procedure. To account for incomplete size segregation in the impactor, the simulation of particles per droplet has been based on the SEM-derived number size distributions (fig. S2), which were merged into 10 size classes: 0 to 1, 1 to 2, 2 to 3, 3 to 4, 4 to 5, 5 to 6, 6 to 7, 7 to 9, 9 to 12, and 12 to 48 μm, covering the full particle size range identified on the impactor stages (D50 = 0.32 to 10 μm) (Fig. 3A).

**Fig. 3.**
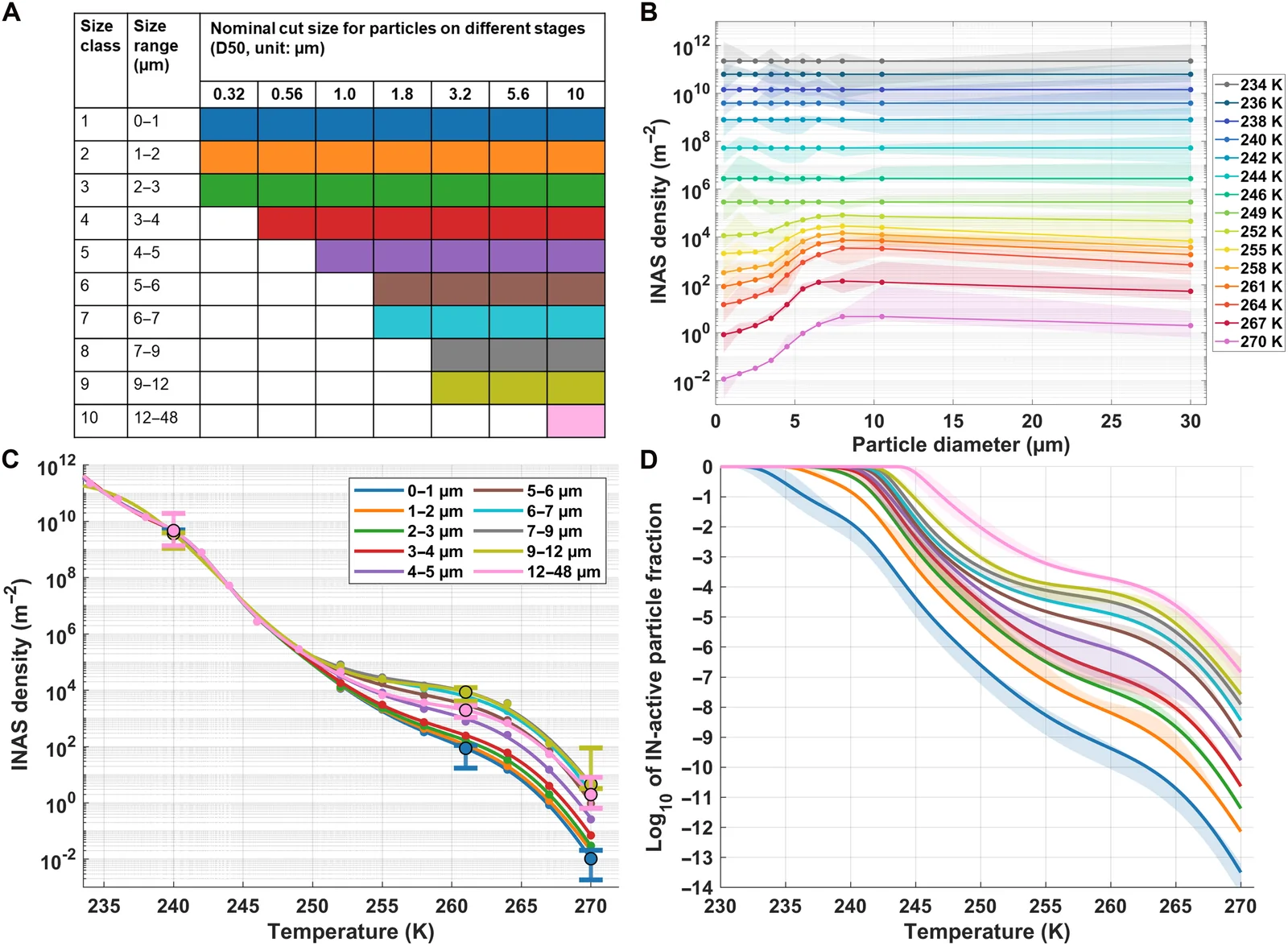
Temperature-dependent size-resolved INAS densities and IN-active particle fractions. (**A**) Particle size classes *i* identified on the different impactor stages, as indicated by the different colors consistent with (C) and (D). (**B**) INAS densities per unit surface area for the ten particle size classes at the temperatures indicated in the legend. The *x* axis indicates the midpoint of each size class (e.g., 30 μm corresponds to the 12- to 48-μm class). Shaded regions indicate uncertainty envelopes of the INAS densities. (**C**) Temperature-dependent INAS densities fitted using a two-Gaussian model with a quadratic baseline for each size class. Vertical bars indicate uncertainty ranges for three representative size classes (0 to 1, 9 to 12, and 12 to 48 μm) at 240, 261, and 270 K. (**D**) Log_10_-transformed IN-active particle fraction, $p_{i}\left(T\right)$, for each size class $\left\{p_{i}\left(T\right)={\text{log}}_{10}\left[F_{i}\left(T\right)\right]\right\}$. Shaded regions indicate uncertainty envelopes of the IN-active particle fractions. The data points in (C) and (D) correspond to those shown in (B). The legend shown in (C) applies also to (D).

Converting INAS densities into IN-active particle fractions via Eq. 3 (Materials and Methods) requires the surface area per particle for each size class ($A_{i,\text{class}}$). These surface areas cannot be estimated assuming spherical particles, as such theoretical surface areas (SSA_th_) would substantially underestimate the true surface areas. These are larger due to particle morphologies, as reflected in BET SSAs of particles collected from the impactor stages (SSA_BET_), which are substantially larger than calculated SSA_th_ (see fig. S16A). Therefore, we corrected the theoretical surface areas using a function that correlates SSA_th_ with SSA_BET_ (section S4 and fig. S16b), yielding more realistic surface areas for all size classes ($A_{i,\text{class}}$) (table S13).

We started the simulation from the assumption of a uniform temperature dependence of INAS densities across all size classes, for which we optimized the agreement of simulated and measured DRINCZ and MINCZ frozen fractions in an iterative procedure. For temperatures where this assumption resulted in either systematically too low or too high frozen fractions depending on size class, we allowed size-dependent INAS densities to achieve agreement between measured and simulated frozen fractions. To fit the INAS densities shown in Fig. 3C at the 15 selected temperatures given in Fig. 3B, we used a two-Gaussian model with a quadratic baseline (Eq. 2); see Materials and Methods for more details).

For temperatures covered by MINCZ, i.e., <250 K, the resulting INAS densities shown in Fig. 3 (B and C) are in agreement with the no-size-dependence assumption, while for temperatures >250 K covered by DRINCZ, INAS densities had to be assumed size-dependent with highest densities for particles ranging from 6 to 12 μm. With this assumption, the simulated DRINCZ frozen fractions agreed well with the measurements (figs. S18 to S24), with remaining discrepancies primarily arising from inconsistencies between the two suspension concentrations measured for each impactor stage. For example, for D50 = 5.6 μm, simulations agreed well with the 0.1 wt % measurements but deviated at 0.05 wt % (fig. S23). For MINCZ, agreement across all concentrations was only obtained for the D50 = 0.32 μm stage (fig. S18). For stages with larger cutoff diameters, good agreement was achieved mainly for the higher-concentrated samples; for instance, D50 = 0.56-μm curves matched poorly at 0.05 wt % but were consistent with the bulk of the experimental data at 0.1 and 0.5 wt % (fig. S19). These inconsistencies likely reflect the underrepresentation of large particles in the MINCZ droplets when samples with larger D50 cutoff diameters were used, and these experiments were therefore excluded from the INAS parameterization (Materials and Methods, table S15, and fig. S5).

The systematic disagreement between simulated and measured DRINCZ frozen fractions for the no-size-dependence assumption is shown in the Supplementary Materials. When choosing an INAS density that reproduces the lowest (D50 = 0.32 μm) or highest (D50 = 5.6 μm) frozen fraction curves well (fig. S25), high discrepancies occurred for the opposite size range (fig. S26), confirming that INAS densities indeed vary with particle size in this temperature range. The increase in INAS densities with particle size up to ∼12 μm, followed by a decrease for even larger particles also aligns with the lower INAS densities observed for the largest impactor stage (D50 = 10 μm) compared with D50 = 5.6 μm (fig. S4).

Figure 3D shows that larger particles exhibit higher IN-active particle fractions across all temperatures. At ∼240 K, only around 1 in 100-μm particles nucleates ice while almost all particles >4 μm nucleate ice. Differences in ice-active particle fractions between size classes grow gradually with increasing temperature, reaching nearly four orders of magnitude at 250 K and even six at 260 K. While the differences below 250 K are solely due to the difference in surface areas (similar INAS density across particle size; Fig. 3C), which covers approximately four orders of magnitude (table S13), the increasing difference for *T* > 250 K is caused by the higher INAS densities of larger particles (Fig. 3C).

The two Gaussian components used to fit INAS densities in Fig. 3C capture two distinct IN activity peaks at low (∼240 K) and high (∼260 K) temperatures, reflecting two characteristic types of ice-nucleating sites. The low-temperature peak at ∼240 K is less pronounced and exhibits no specific size dependence, as the number of active sites scales with particle surface area. In contrast, the more pronounced high-temperature peak near ∼260 K displays the highest INAS densities for particle size classes of 6 to 12 μm. These high-temperature nucleation sites are likely linked to size-dependent morphological features, which we discuss in the following section. The comparison of the simulated INAS densities with previous measurements using KGa-1b (fig. S27) shows a generally good agreement at low temperatures (<245 K) with Murray *et al.* (*17*). At higher temperatures (>250 K), the INAS densities reported by Whale *et al.* (*41*) and Ren *et al.* (*42*) fall within the range typical of particles <5 μm. Yet, particles in the 6- to 12-μm range exhibit INAS densities per unit area approximately one to two orders of magnitude higher than the bulk sample. Notably, although bulk samples are dominated in mass by very large particles, their INAS densities are similar to those of small particles (<5 μm). This counterintuitive behavior is likely related to the morphology of the very large particles, as will be discussed below. The SSA_BET_ values of 11.8 and 11.2 m^2^ g^−1^ reported by Murray *et al.* (*17*) and Whale *et al.* (*41*), respectively, also lie within our measured range and agree best with the value determined for D50 = 1.8 μm (table S12).

### Morphological characteristics of size-segregated particles

Transmission electron microscopy (TEM) and SEM images reveal morphological differences across the size classes investigated (Fig. 4). Submicron particles are mostly translucent in TEM images, consistent with thin platelets composed of only a few unevenly stacked layers. In contrast, particles in the 6- to 12-μm size range frequently exhibit thick, multilayered platelet structures with well-aligned edges, appearing dark in TEM images and well resolved by SEM (Fig. 4, E and F).

**Fig. 4.**
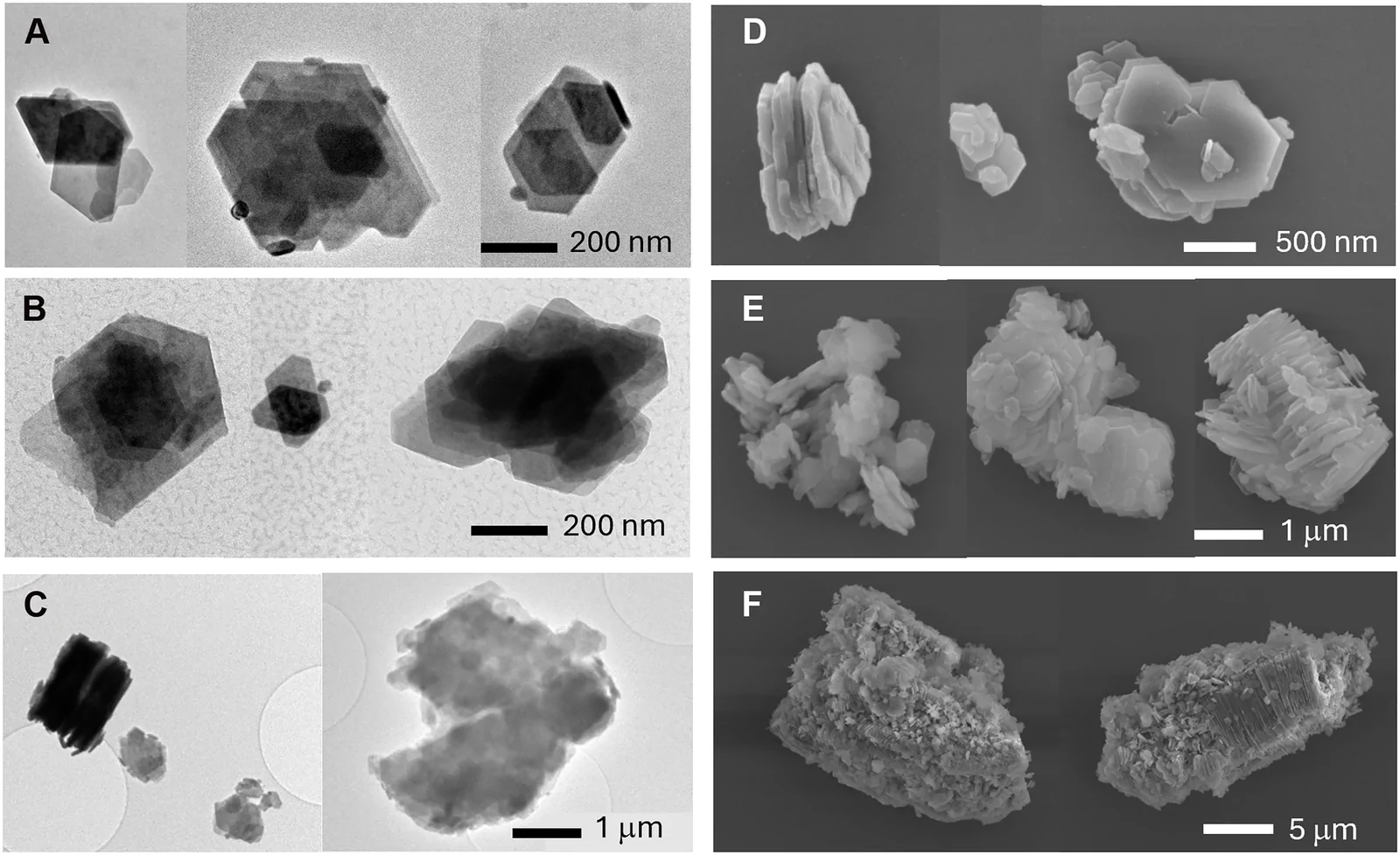
Morphology of kaolinite particles across different size ranges. Selected TEM (**A** to **C**) and SEM images (**D** to **F**) representing morphologies of kaolinite particles from submicron to super micron particles.

For particles larger than ∼12 μm, however, platelets tend to aggregate into more spheroidal shapes rather than forming extended stacks (fig. S42). Our particle counts indicate that 40% of particles >12 μm lack a defined edge-basal distinction (table S16). Notably, the pronounced multilayered edge structures are most prevalent in the 6- to 12-μm size range, corresponding to the size class that exhibits enhanced INAS densities at temperatures above 250 K.

## DISCUSSION

### Morphological features relevant for IN

According to CNT, heterogeneous IN is thermodynamically favored on surfaces that reduce the free-energy barrier for ice embryo formation (*43*). In CNT-based formulations, nucleation is parameterized using an effective contact angle, which serves as a phenomenological descriptor of IN efficiency (*44*). Smaller effective contact angles correspond to surfaces that more efficiently promote ice embryo formation. Consequently, the effective contact angle required for freezing depends strongly on temperature. As supercooling increases and approaches the homogeneous freezing limit, even weakly active sites corresponding to large effective contact angles (∼90°) can induce freezing. At warmer temperatures such as 268 K, however, only highly active sites with much smaller effective contact angles can reduce the nucleation barrier enough to permit freezing (section S5, fig. S17A, and table S14). Simultaneously, the minimum diameter of a nucleation site capable of hosting a critical ice embryo, or to sustain a thermodynamically stable ice embryo, increases with temperature (fig. S17B). Warmer temperatures therefore require larger nucleation site areas. For clay minerals, most INAS are believed to be located on particle edges, which are highly hydroxylated and should therefore favor IN (*29*, *40*, *44*–*46*). In this context, a larger effective surface area for nucleation corresponds to large, multilayer edge structures. Previous studies highlight the critical role of layer stacking: Particles that completely delaminate into single layers in water proved to be IN inactive (*45*), whereas halloysites with well-aligned tubular edges initiate freezing at higher temperatures than kaolinite particles with misaligned stacking (*29*).

The morphological differences across particle size classes provide a plausible explanation for the observed size-dependent INAS densities. De Almeida Ribeiro *et al.* (*47*) have shown that immersion freezing may involve two sequential barriers: the one to form an ice embryo of critical size and the subsequent growth barrier of this embryo into the surrounding water (*47*). CNT calculations suggest that the critical embryo remains relatively small (∼10 nm in diameter even at 270 K), indicating that embryo formation itself is unlikely to be the primary limiting factor for IN on kaolinite at higher temperatures (fig. S17B).

Instead, at relatively warm temperatures (>250 K), the limiting step is more likely the subsequent lateral growth and propagation of the embryo, which requires sufficiently large and continuous active surface areas. A conceptually similar post-nucleation growth limitation has been described for pore condensation and freezing (PCF), where ice must propagate beyond the confined regions in which it initially forms (*47*–*51*). In kaolinite, well-aligned and well-stacked multilayer edge structures may provide sufficiently large and continuous surfaces that enable neighboring embryos to merge, thereby facilitating ice growth and propagation—a mechanism analogous to the one described for PCF in mesoporous silica (*50*). In contrast, particles containing many but poorly aligned layers may fail to form sufficiently extended edge surfaces for efficient nucleation at high temperatures. This interpretation is consistent with previous studies emphasizing the importance of layer stacking and edge alignment for IN activity (*29*, *45*). Thick, multilayered kaolinite platelets shown in Fig. 4 (E and F), which occur predominantly in the 6- to 12-μm size range, may therefore be responsible for the high-temperature freezing mode at ∼260 K. The enrichment of well-aligned multilayered edge structure in this size class could explain the size-dependent INAS densities, increasing the maximum difference in IN-active particle fraction from approximately four orders of magnitude below 250 K to approximately six orders above 250 K (Fig. 3B). This interpretation is further supported by electron microscopy observations showing preferential ice formation at the hydroxylated edges of kaolinite particles rather than on the basal surfaces (*40*). Although these observations were obtained under condensation-freezing conditions, they provide independent evidence that platelet edges are preferential sites for ice initiation in kaolinite.

At lower temperatures (<250 K), IN may occur on less efficient active sites corresponding to larger effective contact angles. Moreover, the surface area required for embryo growth is reduced compared to higher temperatures. Both these effects facilitate IN on smaller particles. In this temperature regime, the agreement between simulated and experimental frozen fractions is compatible with no particle size dependence in INAS density within experimental uncertainty (Fig. 3, C and D).

For particles larger than ∼12 μm, the fraction of particles with well-defined edge structure decreases (table S16). The reduction in well-aligned edge surface area likely accounts for the observed decrease in INAS densities for particles >12 μm at *T* > 250 K (Fig. 3, B and C).

For bulk samples, which contain aggregates and particles substantially larger than 48 μm, irregular morphologies seem again to reduce the abundance of well-defined stacked edge structures (*52*, *53*). Consequently, bulk kaolinite samples (*41*, *42*) exhibit overall INAS densities similar to those of smaller size classes (0 to 5 μm; fig. S27).

### Atmospheric implications

Overall, this study shows that assuming size-independent INAS densities per surface area is a reasonable first-order approximation for mineral dust INPs when the specific nature of nucleation sites is unknown, yet surface area scaling does not necessarily apply. The size-segregated analysis of IN activity reveals two specific IN ranges for kaolinite: a low-temperature range (∼240 K) characterized by size-independent INAS densities and a high-temperature range (∼260 K) in which particles of 6 to 12 μm exhibit an increased abundance of nucleation sites per surface area. We attribute this enhanced high-temperature activity to the prevalence of well-aligned, multilayered platelet edges in this size range, which provide larger, more continuous, and laterally extended hydroxylated edge surfaces that facilitate IN.

In the atmosphere, the size of mineral dust spans a wide range from <0.1 to >100 μm in diameter, with particles from 2 to 20 μm dominating in mass and submicron particles in number, while particles from submicron to ∼20 μm contribute roughly equally to total surface area (*54*, *55*). Under the assumption of size-independent INAS densities, particles covering this size range would therefore contribute similarly to immersion freezing in mixed-phase clouds. Based on our results for kaolinite, this assumption is appropriate for *T* < 250 K, while for higher temperature, particles of 6 to 12 μm should contribute disproportionately to IN.

Several limitations must be considered when extrapolating these findings to atmospheric dusts in general. Supermicron kaolinite particles with well-stacked platelet structures may be relatively scarce in the atmosphere. Moreover, variations in dust particle size are usually accompanied by changes in mineralogical composition (*56*, *57*), which further influences size-dependent IN activity and may outweigh effects associated with particle morphology observed for compositionally pure minerals. Nevertheless, our results highlight that particle morphology alone can induce pronounced size-dependent IN activity even in the absence of compositional variability. Immersion freezing behavior of a single species is often represented by a single active-site parameterization, although it remains unresolved whether particle size itself modifies active-site diversity (*58*). Distinct warm- and cold-temperature freezing regimes are commonly associated with different ice-nucleating components, such as different protein aggregates in Snomax (*59*). In contrast, our results show that compositionally uniform kaolinite can develop distinct freezing regimes solely through size-dependent morphology. The enhanced warm-temperature (>250 K) INAS densities observed for 6- to 12-μm kaolinite particles is associated with the higher abundance of well-stacked particles in this size range.

This site-specific activity echoes the activation behaviors observed in other major atmospheric dust components. For instance, the IN activity of feldspars is dictated by specific surface features, structural defects, and mineralogical heterogeneities rather than bulk geometric surface area (*60*, *61*). These findings support a broader paradigm in which heterogeneous IN across aluminosilicate minerals is governed by localized structural motifs—such as the multilayered edges of kaolinite—rather than macroscale particle dimensions. Given that K-feldspar dominates high-temperature immersion freezing in natural desert dusts (*14*, *15*), these insights raise an important open question: To what extent does particle morphology also modulate the ice-nucleating efficiency of feldspars? This underscores the need for future size-resolved studies that systematically investigate morphology effects across different mineral types and jointly consider size, morphology, and mineralogy. These efforts are essential for refining parameterizations of mineral dust IN and improving the predictive skills of cloud and climate models.

Beyond mineral dust, our results are consistent with previous studies highlighting the role of total nucleation site area in enhancing activity at warm temperatures. Similar findings have been reported for pore condensation freezing in mesoporous materials (*50*) and for biological ice nucleators such as *Pseudomonas syringae* (*62*–*64*). In the latter case, extending individual water-organizing domains did not substantially increase nucleation temperature, whereas lateral alignment of these domains enlarged the effective nucleation area and markedly enhanced efficiency (*62*). In our case, enhanced warm-temperature activity is consistent with the presence of larger, continuous edge areas formed by well-aligned kaolinite platelets. Together, these findings conform with the presence of a second energy barrier that has to be overcome for ice growth into the water volume (*47*).

## MATERIALS AND METHODS

### Size segregation of INPs with the MOUDI impactor

KGa-1b particles from the CMS were aerosolized from the dry powder mixed with bronze beads using a fluidized bed aerosol generator (FBG, Model 3400A; TSI Inc.) and then fed into a MOUDI (MSP Corporation, USA; model 120 MOUDI II) at a rate of 30 ± 0.5 liter min^−1^ with an FBG output flow rate of ∼15 liter min^−1^ supplemented by an additional flow from filtered air. The MOUDI segregated the aerosol particles into 10 stages according to their aerodynamic diameter with cutoff points (D50, the aerodynamic diameter collected with 50% efficiency) ranging from 0.056 to 18 μm under dry suspension conditions. Particles were collected on aluminum foil substrates mounted on each impactor stage. The FBG operation with bronze beads represents a low-energy, nondestructive dispersion technique, which mainly disrupts macroscopic particle agglomerates rather than altering the primary crystal morphology or modifying the surface ice-nucleating sites of kaolinite (*32*, *65*). Deposited particles were gently scraped from the foil surface and transferred into glass vials under dry conditions for subsequent analysis. A comprehensive description of the MOUDI and its collection efficiency for various particle sizes is provided by Marple *et al.* (*66*). Based on the yield (table S2), we selected the seven impactor stages with D50 = 0.32, 0.56, 1.0, 1.8, 3.2, 5.6, and 10 μm to investigate the size-dependent IN ability.

### INP immersion freezing experiments

MINCZ is a microfluidic device designed for picoliter-scale volumes, as described by Isenrich *et al.* (*67*) and Chen *et al.* (*68*). This system enables probing average nucleation site activity of INPs within droplets. Droplets of 75-μm diameter are generated in a polydimethylsiloxane (PDMS) chip and subsequently transferred to fluoropolymer tubing, which minimizes droplet shrinkage by preventing water permeation that typically occurs in PDMS channels. This droplet size allows detection of freezing events down to homogeneous freezing temperatures, enabling the study of immersion freezing almost down to the single-particle level without interference from random impurities. The filled tubing segments, containing on average about 70 to 120 droplets, are placed into custom-made tubing holders and submerged in an ethanol bath for controlled cooling and efficient heat exchange. Temperature is monitored using two thermocouples placed at the same height as the droplets. The droplet temperature is taken as the average of the two thermocouple readings. The average temperature difference between them is used to estimate the temperature uncertainty, which is approximately ±0.2 K (*67*). Temperature is decreased at a cooling rate of 1 K min^−1^, and IN events are detected optically by illumination from above and monitoring intensity changes of the light reflected by a glass slide beneath the droplets. For each size stage and concentration (0.05, 0.1, 0.5, and optionally 1.0 wt %), a suspension was prepared. Before experiments, suspensions were sonicated for 3 min and gently shaken to achieve homogeneous dispersions and to minimize aggregation and sedimentation. Portions of each suspension were used to generate droplets collected into 4 to 10 tubing segments. Freezing experiments were then performed individually for each tubing segment as described above.

The DRINCZ drop-freezing assay uses 96-well polymerase chain reaction trays, with each well containing 50 μl of the particle suspension (*69*). The trays are cooled at a rate of 1 K min^−1^ in an ethanol bath connected to a recirculating chiller and illuminated from below using light-emitting diode lights. Freezing events are identified by the reduction in light transmittance caused by ice formation in the wells. This setup enables accurate recording of heterogeneous freezing events with a temperature resolution of ±0.9 K down to ∼250 K. Around this threshold, nucleation on random impurities sets in DRINCZ is particularly well-suited for detecting the rare, most efficient IN sites within a suspension. For each investigated impactor stage and both tested concentrations (0.05 and 0.1 wt %), three independent suspensions were prepared. The same sonication and shaking pretreatment as for the MINCZ experiments were also applied in the DRINCZ experiments. Each suspension was subjected to three replicate freezing experiments. Between replicates, the suspensions were held at ∼10°C for 10 min to ensure complete melting of previously frozen droplets, resulting in a total of nine freezing curves per concentration and impactor stage.

### Frozen fraction background correction and INAS density calculation

When converting frozen fraction curves to INAS densities, the background freezing curve of pure water was subtracted from that of the KGa-1b samples if their temperature ranges overlapped.

Previous studies have typically performed background corrections after binning the differential nucleus concentration into fixed temperature intervals (e.g., Δ*T* = 0.2° or 0.5°C) (*69*, *70*). However, this approach reduces data resolution.

In this study, we developed an improved background correction method that uses variable temperature intervals without the need for prebinning data points. This approach preserves data resolution, ensuring a more accurate representation of the freezing behavior. The detailed procedure can be found in section S1.

To compare the IN ability of the different impactor stages, we calculated the cumulative INAS densities per unit surface area [*n*_s_(*T*)], based on background-corrected frozen fractions $\left[{\text{FF}}_{\text{corr}}\left(T\right)\right]$ or background-corrected cumulative nucleus concentrations [$K_{\text{corr}}\left(T\right)$, INP numbers per droplet volume] (see section S1)

$$n_{s}\left(T\right)=-\frac{1}{A}\text{ln}\left[1-{\text{FF}}_{\text{corr}}\left(T\right)\right]=\frac{K_{\text{corr}}\left(T\right)\cdot V}{A}$$ (1)

Here, *A* represents the average INP surface area of the particles immersed in each droplet. As the first and last data points—corresponding to the initial and final droplet freezing events—suffer large random variability, they were excluded from the conversion to INAS densities.

### Electron microscopy and particle characterization

We operated the MOUDI for 3 s to collect KGa-1b particles on aluminum foil substrates mounted on the impactor stages. The deposited particles were subsequently transferred from the aluminum foil onto silicon plates for SEM analysis (JEOL JSM7100F) by gently bringing the adhesive side of the silicon plate into contact with the particles. This procedure was used to determine the particle size distribution for impactor stages with D50 = 0.56, 1.0, 1.8, and 3.2 μm. For D50 = 0.32, 5.6, and 10 μm, a sampling time of one minute was required due to the lower particle abundance at these stages.

The size and morphological characteristics of the particles collected on each impactor stage were analyzed using SEM at 3 to 10 kV (figs. S28 to S34). Particles’ major and minor axes were measured from the SEM images using ImageJ to determine size distributions (based on the major axis) and to calculate apparent aspect ratios. Note that SEM images display only two lateral dimensions, which are generally larger than the third (out-of-plane) dimension of most particles. Size distributions divided into 200-nm bins are provided in fig. S2. The total number of particles analyzed ranged from 539 (D50 = 1.0-μm stage) to 3698 (D50 = 10-μm stage). To determine representative apparent aspect ratios depending on particle size, the major axis lengths were grouped into the 10 size classes, and the average aspect ratio was calculated for each of them. These aspect ratios were subsequently used to estimate particle volume and mass. Values are reported in table S11 and visualized in fig. S6.

In addition to major and minor axes measurements, particle thickness was qualitatively assessed using TEM (JEOL JEM 1400, Tungsten filament, 120 kV). Aqueous suspensions of particles from each impactor stage were prepared at concentrations of 0.05, 0.1, and 0.5 wt %, with concentrations tailored to ensure optimal particle density for imaging. One, three, or six droplets of each suspension were deposited onto TEM grids and allowed to dry, leaving the kaolinite particles on the grid surface. Both suspension concentration and droplet number were carefully adjusted to achieve well-dispersed particles while avoiding overcrowding. Representative TEM images for each impactor stage are shown in figs. S35 to S41.

### Surface area determination for INAS density calculations

To convert IN-active particle fractions of the size classes to INAS densities, their surface areas (*A*_i,class_) are required. To this end, SSAs of all evaluated impactor stages were measured (SSA_BET_) to establish a correlation with their theoretical SSA (SSA_stage,th_), which were calculated on the basis of the number size distributions of particles collected on each impactor stage. In calculating SSA_stage,th_, we assumed spherical particle shapes, estimating the average particle diameter from the major length and aspect ratio and using a kaolinite density of $\rho_{\text{KGa}-1b\;}$= 2.68 × 10^3^ kg/m^3^ (*71*).

The SSA_BET_ of all investigated impactor stages were measured using the BET gas adsorption method (*72*), following DIN-ISO 9277 and DIN 66131 standards. Krypton (Kr) was used as the adsorbate due to its lower detection limit compared with nitrogen (N_2_), allowing accurate measurement of limited sample quantities. Measurements were conducted by 3P Instruments GmbH & Co. KG (Odelzhausen, Germany), and results are provided in table S12. The detailed calculation equations and procedures are outlined in Gao *et al.* (*73*), Hwang and Barron (*74*), and Stephen *et al.* (*72*).

Figure S16 shows the measured and theoretical SSAs for each impactor stage (fig. S16A) and the correlation between theoretical and measured values (fig. S16B), from which a four-parameter Boltzmann function was fitted. The fitted curve was then used to convert SSA_th_ to the actual SSAs for each size class SSA_class_ (section S4.1). The estimated average surface area per particle in each size class and its associated uncertainty range are summarized in table S13. With these values, the size-dependent IN-active particle fractions were converted to size-dependent INAS densities.

### Simulation of size-dependent INAS densities and the fraction of IN-active particles

To simulate the MINCZ and DRINCZ freezing curves, we used an iterative procedure by adjusting and optimizing the INAS densities per surface area to best match the MINCZ and DRINCZ frozen fractions covering the temperature range from 235 to 270 K and across all suspension concentrations and impactor stages. During this procedure, freezing curves that were incompatible with the bulk of the freezing data—due to few or no particles present in MINCZ droplets at low concentrations—were excluded (table S15 and fig. S5).

We prescribed INAS densities [$n_{s,i}\left(T\right)$] at 15 selected temperatures (234 to 246 K in 2 K steps and 249 to 270 K in 3 K steps) for the 10 size classes and fitted them using a two-Gaussian model with a quadratic baseline

$${\text{log}}_{10}\left[n_{s,i}\left(T\right)\right]=aT^{2}+\mathrm{bT}+c+A_{1}e^{-\frac{{\left(T-\mu_{1}\right)}^{2}}{2\sigma_{1}^{2}}}+A_{2}e^{-\frac{{\left(T-\mu_{2}\right)}^{2}}{2\sigma_{2}^{2}}}$$ (2)

The quadratic baseline ensures a monotonic increase in INAS density toward lower temperatures. Parameters a, b, and c describe the curvature, slope, and vertical offset of the baseline, respectively. The two Gaussian functions capture the distinct INAS density peaks in the low- and high-temperature regimes; $A_{1,2}$ set the peak amplitudes, $\mu_{1,2}$ define the peak centers, and $\sigma_{1,2}$ control the peak widths.

Using the fitted expressions for $n_{s,i}\left(T\right)$, we calculated the fraction of IN-active particles in each size class as

$$p_{i}\left(T\right)={\text{log}}_{10}\left\{1-\text{exp}\left[-A_{i,\text{class}}\cdot n_{s,i}\left(T\right)\right]\right\}$$ (3)

where $A_{i,\text{class}}$ is the surface area per particle for size class i (table S13). The IN-active particle fraction, $F_{i}\left(T\right)=10^{p_{i}\left(T\right)}$, which quantifies the fraction of particles within each size class that nucleate ice at a given temperature was then incorporated into a probabilistic frozen-fraction model

$${\mathrm{FF}}_{\text{sim}}\left(T\right)=1-\prod_{i=1}^{10}{\left[1-F_{i}\left(T\right)\right]}^{N_{i}}$$ (4)

where $N_{i}$ is the number of particles of size class i per droplet, obtained from merged size bins for DRINCZ (tables S4 to S10) and from Monte Carlo simulations for MINCZ. Unlike the use of Monte Carlo methods to evaluate freezing kinetics and parameter stochasticity across populations (*75*), our simulation here explicitly accounts for the droplet-to-droplet variability in particle population similar to the approach of Alpert and Knopf (*76*). Under the singular (site-specific) framework (*77*), this stochastic particle distribution inherently shapes the macroscopic frozen fraction. This statistical treatment is particularly crucial for the microfluidic system (MINCZ), where a low average number of particles per droplet amplifies sampling fluctuations.

The INAS densities were optimized by minimizing the error between simulated and observed frozen fractions, first by assuming size-independent INAS densities for all 15 selected temperatures and then allowing temperature dependence if required. Optimization proceeded sequentially across impactor stages, starting with the smallest cutoff (D50 = 0.32 μm) to determine $n_{s,i}\left(T\right)$ for size classes i=1 to 3. All freezing curves from this stage were used (see table S15), with the resulting simulations shown in fig. S18. We then optimized the INAS density for the next size class using progressively larger D50 values: 0.56 μm for $n_{s,4}\left(T\right)$ (excluding the MINCZ 0.05 wt % curves, as shown in table S15), 1.0 μm for $n_{s,5}\left(T\right)$, 1.8 μm for $n_{s,6}\left(T\right)$ and $n_{s,7}\left(T\right)$, 3.2 and 5.6 μm for $n_{s,8}\left(T\right)$ and $n_{s,9}\left(T\right)$, and 10 μm for $n_{s,10}\left(T\right)$, as summarized in Fig. 3A. The resulting simulated freezing curves are shown in figs. S18 to S24. The optimized INAS densities exhibit size-independence for *T* < 250 K and size dependence for *T* > 250 K. Details of the uncertainty analysis for the particle surface area $\left(A_{i,\text{class}}\right)$, IN-active particle fraction $\left[F_{i}\left(T\right)\right]$, and the derived INAS densities $\left[n_{s,i}\left(T\right)\right]$ are provided in section S4.

To assess the best fit for the assumption of size-independent INAS densities, we carried out a series of simulations in which the INAS density was held constant for all particle sizes. The constant INAS density was varied systematically by shifting the INAS-density curve vertically (fig. S25). For each tested value, simulated frozen fraction curves were compared against the DRINCZ measurements for each impactor stage. The lower bound of the constant-INAS-density range was defined as the smallest value that allowed the simulation to reproduce the lowest frozen fraction curve (D50 = 0.32 μm). Conversely, the upper bound was defined as the largest value capable of reproducing the highest frozen fraction curve (D50 = 5.6 μm). No single constant-INAS-density value within this range was able to reproduce all size classes simultaneously (fig. S26).

## Supplementary Materials

### This PDF file includes:

Supplementary Text Figs. S1 to S42 Tables S1 to S16 References
