# Supplementary material for: Ice nucleation by kaolinite: Morphology shapes the size dependence of ice nucleation active sites on dust particles
Source: Sci Adv. 2026 Oct 14;12(42):eaeg1206. doi: 10.1126/sciadv.aeg1206 (PMC13666003; doi:10.1126/sciadv.aeg1206)
Supplement: Supplementary file 1 [file sciadv.aeg1206_sm.pdf]

Science Advances
AAAS

Supplementary Materials for

Ice nucleation by kaolinite: Morphology shapes the size dependence of ice nucleation active sites on dust particles

Yuhang Song *et al.*

Corresponding author: Yuhang Song, yuhang.song@env.ethz.ch; Claudia Marcolli, claudia.marcolli@env.ethz.ch

Sci. Adv. **12**, eaeg1206 (2026)
DOI: 10.1126/sciadv.aeg1206

This PDF file includes:

Supplementary Text
Figs. S1 to S42
Tables S1 to S16
References

Supplementary Text

S1. Background correction based on variable temperature intervals

To establish the pure-water background for DRINCZ and MINCZ, we constructed a normalized frozen-fraction background curve (FF_{BGfit}) for each instrument by merging all pure-water experiments (Molecular Biology Reagent, W4502, Sigma-Aldrich; hereafter referred to as SA water) conducted with that instrument into a single dataset. Specifically, nine SA water experiments were merged for DRINCZ and 23 for MINCZ, and the resulting datasets were each fitted using a three-parameter Boltzmann function to obtain the corresponding background curves.

$$FF_{BGfit}(T, a, b, c) = \frac{1}{(1+e^{a(T-b)})^c}, \tag{S1}$$

with T representing the freezing temperature. The fitting parameters a, b, and c correspond to the slope of the fit, the inflection point and the asymmetrical factor, respectively, and are summarized in **Table S1** for DRINCZ and MINCZ background curves. The merged pure water data and the corresponding fit curves are shown in **Figure S1**. Using the fitted frozen fraction, the background cumulative nucleus concentration ($K_{bg}(T)$) for the SA water model is given as:

$$K_{bg}(T) = -\frac{\ln[1-FF_{BGfit}(T)]}{V} \tag{S2}$$

with V representing the droplet volume (50 µL for DRINCZ and 220 pL for MINCZ). The corresponding differential nucleus concentration ($k_{bg}(T)$) is then calculated as

$$k_{bg}(T) = \frac{K_{bg}(T_2)-K_{bg}(T_1)}{\Delta T}. \tag{S3}$$

Here, ΔT represents the variable temperature intervals, which are specific for each experiment and therefore vary by case.

The differential nucleus concentration ($k_{sam}(T)$) for each sample solution is directly calculated from the frozen fraction curves as:

$$k_{sam}(T) = -\frac{1}{V\Delta T} \cdot \ln(1 - \frac{\Delta N}{N(T)}). \tag{S4}$$

The number of unfrozen droplets ($N(T)$) before a temperature step ΔT, the number of droplets that freeze during the temperature step (ΔN), and ΔT are taken from the freezing experiment. The ΔT values from the experiment are then inserted in Eq.(S3) to calculate a $k_{bg}(T)$ curve that is adapted to the experiment.

With $k_{bg}(T)$ and $k_{sam}(T)$, the background-corrected differential nucleus concentration ($k_{corr}(T)$) is calculated as:

$$k_{corr}(T) = k_{sam}(T) - k_{bg}(T) \tag{S5}$$

The background-corrected cumulative nucleus concentration ($K_{corr}(T)$) and the background-corrected frozen fraction ($FF_{corr}(T)$) can be calculated as:

$$K_{corr}(T) = \sum_{0}^{T} k_{corr}(T) \cdot \Delta T \quad \text{(S6)}$$

$$FF_{corr}(T) = 1 - \exp[-K_{corr}(T) \cdot V] \quad \text{(S7)}$$

S2. Retrieval of mean frozen fraction curves and ice nucleation active site (INAS) densities

To compare freezing behavior across the different impactor stages and suspension concentrations, the data obtained from individual experiments – frozen fractions (*FF*) and INAS densities – were merged into single datasets.

To determine mean frozen fraction curves for each investigated impactor stage and suspension concentration, *FF* values of individual experiments (prior to background correction) were extracted at fixed *FF* values. This approach differs from that used in previous studies (*68, 78*), which averaged FF values at fixed temperatures. In DRINCZ measurements, the fixed *FF* values were set to 1/96, 2/96…95/96, corresponding to the freezing of the 96 wells in a PCR tray. For MINCZ measurements, the *FF* values were set to $1/N_{av}$, $2/N_{av}$ … $(N_{av-1})/N_{av}$, where N_{av} represents the average number of data points per experiment for each impactor stage (**Table S3**). The freezing temperature at each *FF* value within a dataset was determined via linear interpolation between the measured *FF* data points above and below the fixed *FF* value. The freezing temperature for each *FF* value was obtained by averaging the interpolated temperatures across datasets to produce a merged *FF* curve. The average and individual frozen fraction curves for DRINCZ are shown in both **Figure 1** of the main text and **Figure S3**, while those for MINCZ are shown in **Figure 2** of the main text.

To obtain mean INAS densities, *FF* curves of individual experiments were firstly background corrected. Merged *FF* curves were then obtained using the same method described above and subsequently converted into cumulative nucleus concentrations as described in Sect. S1. The cumulative nucleus concentrations were refined by applying upper and lower FF limits to remove outliers and then converted into INAS densities per unit surface area using Eq. (1) of the main text. Note that *FF* curves that approach background values were excluded from INAS averaging, as only a limited number of valid data points remained after background correction. This filtering step may introduce a high bias in the averaged INAS densities. The averaged INAS densities per surface area and the corresponding individual datasets for DRINCZ experiments are shown in **Figure S4**. The INAS densities per surface area for all freezing experiments are presented in **Figure S5**.

The stage-resolved INAS densities shown in **Figure S5** were used for two auxiliary purposes: (i) to identify datasets with particle loadings deviating from the nominal concentrations, enabling exclusion of non-representative cases from the subsequent optimization, and (ii) to provide

physically reasonable initial estimates of the INAS densities, $n_s(T)$, for the parameterization procedure. Importantly, the stage-resolved INAS densities (**Figure S4** and **Figure S5**) were not used as the basis for parameterization. The parameterization was instead performed directly on the experimental frozen-fraction curves. Therefore, data processing steps applied to the stage-resolved INAS densities (e.g., removal of outliers) do not affect the optimization or final parameterization results.

S3. Particle number and size distribution of particles in MINCZ and DRINCZ droplets

Using the particle size distributions obtained from SEM images, we calculated the average number of particles per size bin in sample aliquots of DRINCZ and MINCZ experiments. **Tables S4–S10** show that DRINCZ droplets – owing to their large volume (50 µL) – can accommodate the full range of particle sizes that were detected by electron microscopy on the respective impactor stages, enabling direct calculation of average particle numbers per droplet. In contrast, the much smaller volume of MINCZ droplets (~75 µm diameter) introduces greater variability in particle number and size distribution. To address this, we used the particle size distributions to compute the total number of particles in a bulk suspension with a volume corresponding to 2000 droplets (V_{susp} = 2000 x V_{MINCZ}) and then used a Monte Carlo simulation to distribute these particles randomly between the 2000 droplets to simulate MINCZ droplet populations with random particle occupation.

The number of particles in the bulk suspension was calculated based on the particle size distributions shown in **Figure S2** such that the total mass of all particles corresponds with the respective suspension concentration. Assuming spherical geometry, the mass of an individual particle in bin j (m_j) was calculated as:

$$m_j = \frac{\pi}{6}\left(\frac{a_j + \frac{a_j}{\alpha_j}}{2}\right)^3 \cdot \rho_{KGa-1b} \qquad \text{(S8)}$$

where ρ_{KGa-1b}= **2.68 × 10^3 kg/m^3** is the mass density of KGa-1b (*71*), and $\boldsymbol{a_j}$ is the apparent major axis of the particles. The corresponding minor axis for each of the ten size classes was obtained by dividing $\boldsymbol{a_j}$ by the average aspect ratio (α_j), as listed in **Table S11** and illustrated in **Figure S6**. For instance, an aspect ratio of 1.405 was assigned to particles in size class 1 (0–1 µm) while a ratio of 1.649 was used for particles in size class 6 (5–6 µm).

With the particle mass ($\boldsymbol{m_j}$) and occurrence probability ($\boldsymbol{P_j}$) taken from the particle size distributions in **Figure S2**, we calculated the mass contribution ratio ($\boldsymbol{R_j^m}$) of each bin j to the total mass for the different impactor stages as:

$$R_j^m = \frac{m_j \cdot P_j}{\sum_{j=1}^{n}(m_j \cdot P_j)} \qquad \text{(S9)}$$

For the different experimental suspension concentrations ($\boldsymbol{C_{susp}}$ = 0.05 wt%, 0.1 wt%, 0.5 wt%, and 1.0 wt%) the total mass ($\boldsymbol{m_{total,j}}$) that each size bin j contributes to the total solute mass ($\boldsymbol{m_{solute}}$) for a suspension volume V_{susp} is given as:

$$m_{total,j} = m_{solute} \cdot R_j^m = \rho_{water} \cdot V_{susp} \cdot C_{susp} \cdot R_j^m \quad \text{(S10)}$$

Finally, the particle number that each size bin contributes to the total number of particles in V_{susp} equals:

$$N_j = round(\frac{m_{total,j}}{m_j}) \quad \text{(S11)}$$

with N_j rounded to the closest natural number. To simulate random particle occupation in droplet populations of MINCZ experiment, we divided the suspension volume V_{susp} into 2000 virtual droplets and distributed N_j particles for each bin j across the droplets. From this pool of 2000 droplets, 150 were randomly selected to represent a MINCZ droplet population. This process was repeated twice with the remaining droplets to emulate a total of three independent experiments, enabling a statistically robust estimate of the particle size distribution within individual MINCZ droplets. A schematic of the simulation workflow is provided in **Figure S7**. The simulated particle occupation of MINCZ experiments for all investigated impactor stages are shown in **Figure S8–Figure S14**.

S4. Estimation and uncertainty analysis of particle surface area ($A_{i,class}$), IN-active particle fraction ($F_i(T)$), and INAS density ($n_{s,i}(T)$) for each size class

S4.1. Estimation and uncertainty analysis of size-class particle surface areas ($A_{i,class}$)

S4.1.1. Estimation of actual particle surface areas

1) Calculation of theoretical specific surface area for each impactor stage ($SSA_{stage,th}$)

To derive specific surface areas for each particle size class *i*, we corrected their theoretical specific surface areas ($SSA_{i,th}$), calculated assuming spherical geometry, using an experimentally derived factor. This factor accounts for the higher specific surface areas measured with the BET method for each impactor stage ($SSA_{stage,BET}$). It was obtained by establishing a functional relationship between the measured BET surface area ($SSA_{stage,BET}$) and their corresponding theoretical values ($SSA_{stage,th}$).

The theoretical SSAs for each impactor stage ($SSA_{stage,th}$) were calculated based on the particle size distribution for the stages (**Figure S2**), which present the major-axis lengths grouped into 0.2 µm bins. The corresponding minor axis was then determined based on the representative aspect ratio of its size class (**Figure S6**), allowing calculation of the mean particle diameter for each bin. Assuming a bulk density of 2.68×10^3 kg m^{-3} for kaolinite (*71*), the mass ($\boldsymbol{m_j}$) and surface area ($\boldsymbol{A_j}$) of a single particle in each bin *j* were estimated. The representative theoretical mass and surface area of a particle ensemble within a given stage were obtained by weighting $\boldsymbol{m_j}$ and $\boldsymbol{A_j}$ by the occurrence probability ($\boldsymbol{P_j}$) of each size bin and summing across all bins. The theoretical specific surface area for the stage was then calculated as the ratio of the weighted surface area to the weighted mass:

$$SSA_{stage,th} = \frac{\sum_j A_j \cdot P_j}{\sum_j m_j \cdot P_j} \tag{S12}$$

2) Establishment of the function to convert theoretical to BET-corrected surface areas

The BET-measured and theoretical SSA for each impactor stage are listed in **Table S12** and visualized in **Figure S16a**. The comparison reveals that SSA_{BET} values are substantially larger than $SSA_{stage,th}$, due to the additional surface area contributed by features such as cracks and pores (*79*). To obtain more realistic surface area estimates, we correlated SSA_{BET} with $SSA_{stage,th}$ using a four-parameter Boltzmann function:

$$SSA_{BET}(x) = \frac{A_1 - A_2}{1 + e^{(x - x_0) \cdot k}} + A_2 \tag{S13}$$

where A_1 and A_2 are the asymptotic limits, x_0 is the inflection point, k determines the slope, and x represents SSA_{th}. The final fit is shown in **Figure S16b**, and the resulting correction function is given by:

$$SSA_{BET}(x) = \frac{-7.164}{1+e^{5.43(x-0.711)}} + 13.66 \tag{S14}$$

3) Calculation of actual particle surface area for each size class ($A_{i,class}$)

To estimate the actual surface area per particle in each size class—used for calculating size-dependent INAS densities per unit surface area—we applied the following procedure.

The actual surface area (**$A_{i,class}$**) per particle for each size class i was determined based on the finer size bins j of 0.2 µm. The relative occurrence probability (P_j^i) of each size bin j within a given size class i was derived from the combined particle size distributions of all impactor stages, consistent with the calculation of average aspect ratios shown in **Figure S6**. The mass of a theoretical spherical particle (m_j) in bin j was calculated using Eq. (S8). The corresponding actual specific surface area ($SSA_{j,act}$) was obtained by correcting the theoretical values with the fitted conversion function Eq. (S14):

$$SSA_{j,act} = f(SSA_{j,th}) \tag{S15}$$

The actual surface area of a single particle in bin j was then calculated as

$$A_{j,act} = m_j \times SSA_{j,act} \tag{S16}$$

Finally, the actual surface area per particle in each size class ($A_{i,class}$) was obtained by weighting with the occurrence probability and summing across all size bins:

$$A_{i,class} = \sum_j P_j^i \cdot A_{j,act} \tag{S17}$$

S4.1.2. Monte Carlo estimation of surface-area uncertainty

The estimation of actual particle surface area involves multiple sources of uncertainty, including uncertainties associated with SEM-derived particle counting statistics, BET surface-area measurements, and the theoretical-to-BET correction function. Explicit analytical propagation of all these coupled uncertainties through the full calculation procedure would be very complicated. Therefore, a Monte Carlo framework was used to account for the combined effects of these uncertainty sources on the estimated particle surface areas. The complete calculation sequence described above was repeated 5000 times, including resampling of SEM-derived particle size distributions, perturbation of BET-measured SSA values within the instrumental uncertainty range (±1%), and refitting of the theoretical-to-BET correction function in each iteration. The 5th and

95th percentiles of the resulting actual surface-area distributions were then taken as the lower and upper uncertainty bounds, respectively. This percentile-based approach has been widely used to characterize uncertainty ranges in atmospheric measurements and simulation results *(80, 81)*. In this way, the cumulative effects of the major uncertainty sources involved in the surface-area estimation process were incorporated into the final uncertainty range. Similar approaches were adopted in previous studies *(75, 81)*. Each Monte Carlo iteration included the following steps:

1) Resampling of SEM-derived particle size distributions

The SEM-derived particle counts of each stage were resampled using multinomial resampling while preserving the original total particle number of each stage. This procedure generated new realizations of stage-based particle size distributions.

2) Recalculation of theoretical SSA and refitting of the correction function

Using the resampled particle size distributions, the theoretical SSA of each stage was recalculated according to Eq. (S12). The measured Kr-BET SSA values were simultaneously perturbed within the instrumental uncertainty range (±1%) using a uniform random distribution. The relationship between theoretical SSA and BET-measured SSA was then refitted using Eq. (S13), generating a new theoretical-to-BET correction function.

3) Recalculation of corrected particle surface area for each size class

The resampled particle counts from all stages were combined to construct updated global particle-size distributions. Using the new correction function, the corrected actual particle surface area for each size class ($A_{i,class}$) was recalculated according to Eqs. (S15)–(S17).

4) Estimation of uncertainty intervals

After 5000 iterations, distributions of corrected particle surface area were obtained for all size classes. For each size class, the median corrected surface area was used as the representative value, while the 5th and 95th percentiles were taken as the lower (A_{lower}) and upper (A_{upper}) uncertainty bounds, respectively. The resulting actual particle surface areas ($A_{i,class}$) and corresponding uncertainty ranges for all size classes are summarized in **Table S13**.

S4.2. Estimation of uncertainty in IN-active particle fraction ($F_i(T)$)

The optimized IN-active particle fraction for each size class, $F_i(T)$, was derived by fitting the measured frozen fraction curves. To obtain a representative uncertainty range for $F_i(T)$, we fitted the upper and lower bounds of the frozen fraction curves estimated from replicate freezing experiments. For each D50 stage and concentration, 4–10 frozen fraction datasets were available. Following the procedure described in SI Appendix, Sect. 2, all datasets were interpolated at fixed FF values, and the corresponding freezing temperatures were determined by linear interpolation between neighboring measured data points. The 5th and 95th percentile temperatures among replicate datasets were then calculated at each fixed FF value, yielding the corresponding 5th and 95th percentile frozen fraction curves for each D50 stage and concentration.

A two-Gaussian model with a quadratic baseline was then used to simulate the INAS density, $n_{s,i}(T)$, at each temperature. The median particle surface area (A_{median}) was adopted to calculate $F_i(T)$ using Eq. 3, followed by simulation of the frozen fraction, $FF_{sim}(T)$, with the probabilistic frozen fraction model (Eq. 4). The simulated $FF_{sim}(T)$ curves were then fitted separately to the 5th and 95th percentile frozen fraction curves for each stage using the same optimization procedure described in Methods. This yielded the uncertainty envelope of $F_i(T)$, which are shown as shaded regions in **Fig. 3d**.

S4.3. Propagation of uncertainty to INAS density ($n_{s,i}(T)$)

The uncertainty in $n_{s,i}(T)$ was estimated by combining the uncertainty in $F_i(T)$ with the independently derived uncertainty range of the corrected particle surface area.

As described in Sect. S4.2, lower and upper bounds of $F_i(T)$ were obtained for each size class. These bounds were combined with the lower and upper uncertainty bounds of $A_{i,class}$ (**Table S13**) to estimate the corresponding uncertainty range in $n_{s,i}(T)$. The simulated upper and lower bounds of INAS density derived from the uncertainty range of $F_i(T)$ are denoted as $n_{s,i}(T)_{upper}$ and $n_{s,i}(T)_{lower}$, respectively. Since all $F_i(T)$ values were calculated using A_{median}, the upper

uncertainty bound of $n_{s,i}(T)$ was corrected using the factor A_{median}/A_{lower}, whereas the lower uncertainty bound was corrected using A_{median}/A_{upper}.

The resulting uncertainty ranges for each size class at 15 selected temperatures are shown as shaded regions in **Fig. 3b**. In addition, vertical bars indicate the uncertainty ranges for three representative size classes (0–1, 9–12, and 12–48 μm) at 240, 261, and 270 K (**Fig. 3c**). These size classes represent the low-INAS regime (0–1 μm), the peak-INAS regime (9–12 μm), and the declining-INAS regime beyond the peak (12–48 μm), respectively.

S5. Contact angle, critical embryo diameter and stable embryo diameter required at different temperatures based on classical nucleation theory (CNT)

To quantify the conditions for ice embryo formation and growth at different temperatures, we calculated the effective contact angle (α) between the ice embryo and the substrate surface based on **classical nucleation theory (CNT)**. Here, α should be considered as a phenomenological descriptor of ice nucleation efficiency rather than as a directly measurable physical property. The effective contact angle α ranges from 0° to 180°. Smaller values correspond to surfaces that more efficiently stabilize ice embryos and promote ice nucleation, whereas larger values indicate weaker ice nucleation activity. An effective contact angle of 180° corresponds to the homogeneous freezing limit, where the surface provides no enhancement to ice nucleation. Additionally, we estimated the diameters of the critical and stable ice embryos at different temperatures to investigate the characteristic size of nucleation sites required for freezing.

According to CNT, the homogeneous ice nucleation rate for supercooled water ($j_{hom}(T)$) is expressed as (*18*)

$$j_{hom}(T) = \frac{kT}{h}\exp[-\frac{\Delta F_{diff}(T)}{kT}] \times n_v \exp[-\frac{\Delta G(T)}{kT}], \quad \text{(S18)}$$

where *k* and *h* represent the Boltzmann and Planck constants, respectively. *T* is the absolute temperature and n_v is the number density of water molecules ($n_v = 3.1 \times 10^{22} cm^{-3}$). $\Delta F_{diff}(T)$ denotes the diffusion activation energy required for a water molecule to cross the water–ice embryo interface while the whole first term (before the times sign) describes the diffusive flux of water molecules to the ice embryo. Following Smith *et al.* (*82*), Zobrist *et al.* (*83*) , and Atkins *et al.* (*84*), the diffusion activation energy is given by

$$\Delta F_{diff}(T) = \frac{kT^2 E}{(T-T_0)^2}. \quad \text{(S19)}$$

For liquid water, we adopt E = 892 K and T_0 = 118 K, as determined by Smith *et al.* (*82*) and valid in the temperature range of 150–273 K.

In Eq. (S18), the term after the multiplication sign denotes the concentration of critical (viable) ice embryos present at the nucleus-water interface. $\Delta G(T)$ can be expressed as (*85*)

$$\Delta G(T) = \frac{16\pi}{3}\frac{v_{ice}{}^2(T)\sigma_{sl}{}^3(T)}{[kTlnS(T)]^2}. \quad \text{(S20)}$$

Here, $v_{ice}(T)$ is the molecular volume of H_2O in ice (values adopted from Zobrist *et al.* (*83*)), and $\sigma_{sl}(T)$ is the interfacial tension between water and the ice embryo. The interfacial tension $\sigma_{sl}(T)$ follows Reinhardt *et al.* (*86*). The ice saturation ratio $S(T)$ is defined as

$$S(T) = \frac{p_{H2O}(T)}{p_{ice}(T)}, \quad \text{(S21)}$$

where $p_{H2O}(T)$ and $p_{ice}(T)$ are the vapor pressure of supercooled liquid water and ice, respectively. Parameterizations for $p_{H2O}(T)$ and $p_{ice}(T)$ were taken from Murphy and Koop (*87*).

In the presence of an ice nucleus, the heterogeneous ice nucleation rate ($j_{het}(T)$) can be expressed as (*18*):

$$j_{het}(T) = \frac{kT}{h}\exp[-\frac{\Delta F_{diff}(T)}{kT}] \times n_s \exp[-\frac{\Delta G(T) \times f_{het}}{kT}], \quad \text{(S22)}$$

where $n_s(\approx 10^{15}$ cm^{-2}) denotes the number density of water molecules at the ice nucleus–water interface, and $f_{het}(\leqslant 1)$ is the compatibility function that quantifies the reduction in the Gibbs free energy barrier due to the presence of the ice nucleus. The compatibility function is expressed as follows (*85*):

$$f_{het} = \frac{1}{4}(2 + cos\alpha)(1 - cos\alpha)^2 \quad \text{(S23)}$$

The variation of the contact angle α from 0° to 180° corresponds to f_{het} values ranging from 0 (perfect compatibility) to 1 (no compatibility).

Kaufmann *et al.* (*88*) estimate that $j_{het}(T)$ ranges from 10^9 to $10^{13} cm^{-2}s^{-1}$ when assuming that a single active site in the sample initiates ice nucleation. In our analysis, we presume a heterogeneous nucleation rate of $1.0 \times 10^{10} cm^{-2}s^{-1}$ ($j_{het\_thr} = 1.0 \times 10^{10} cm^{-2}s^{-1}$) to determine the threshold contact angle (α_{thr}). The contact angle α was then decreased incrementally from 180° to 0° in steps of 0.1°, and the α value at which $j_{het}(T)$ first exceeded $j_{het,thr}$ was defined as α_{thr}. This procedure was repeated for each temperature between 234 K and 273 K at 1 K intervals.

The resulting α_{thr} values at each temperature are summarized in **Table S14**, which shows that smaller contact angles are required at higher temperatures, indicating that ice nucleation at warmer temperatures demands greater compatibility between the ice nucleus and water. A linear fit between temperature and α_{thr} yielded a slope of −2.1869 and an intercept of 612.805 (**Figure S17a**). The temperature-dependent threshold contact angle ($\alpha_{thr}(T)$) is expressed as:

$$\alpha_{thr}(T) = -2.1869T + 612.805. \quad \text{(S24)}$$

The coefficient of determination (R^2) of the linear fit is 0.9911.

By setting the $dG/dr = 0$, we obtained the radius of the critical ice embryo (r_{cri}) for homogeneous freezing:

$$r_{cri} = \frac{2v_{ice}(T)\sigma_{sl}(T)}{kTlnS(T)}. \quad \text{(S25)}$$

For heterogeneous freezing, the corresponding diameter of the critical ice embryo ($D_{cri}^{het}(T)$) on a substrate is given by

$$D_{cri}^{het}(T) = 2r_{cri}\sin(\alpha_{thr}(T)). \quad \text{(S26)}$$

However, an ice embryo of critical size remains metastable because $\Delta G(T) \geq 0$, as also discussed by Marcolli (*51*). It must grow further until $\Delta G(T) \leq 0$ to become thermodynamically stable. Assuming spherical growth, by setting $\Delta G(T) = 0$, the diameter of a stable ice embryo ($D_{stb}(T)$) is therefore

$$D_{stb}(T) = \frac{6v_{ice}(T)\sigma_{sl}(T)}{kTlnS(T)}. \quad \text{(S27)}$$

Figure S17b shows that the diameters of both critical ($D_{cri}^{het}(T)$) and stable ($D_{stb}(T)$) ice embryos increase with temperature, indicating that formation of a critical and a stable ice embryo at warmer temperatures require larger surface areas for nucleation.

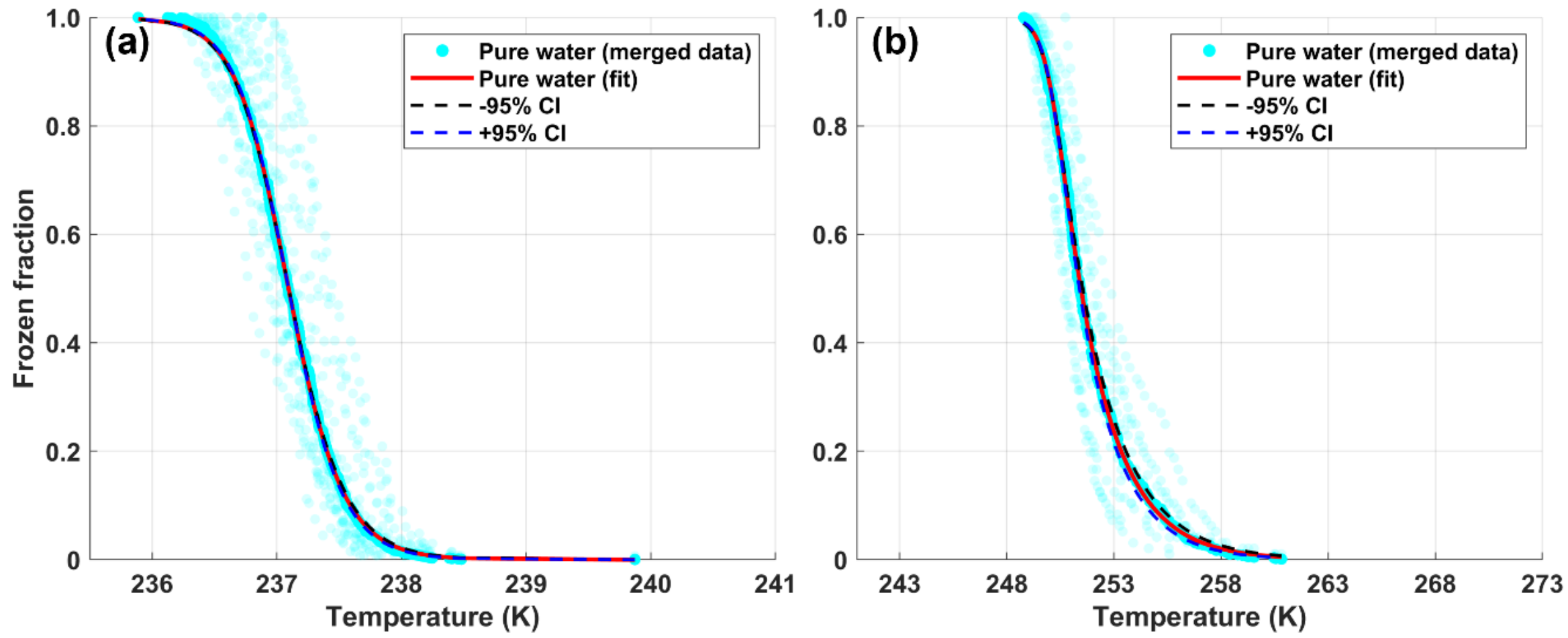

Figure S1. Merged pure-water data and corresponding Boltzmann fits for (a) MINCZ and (b) DRINCZ experiments, together with their 95% confidence intervals (±95% CI).

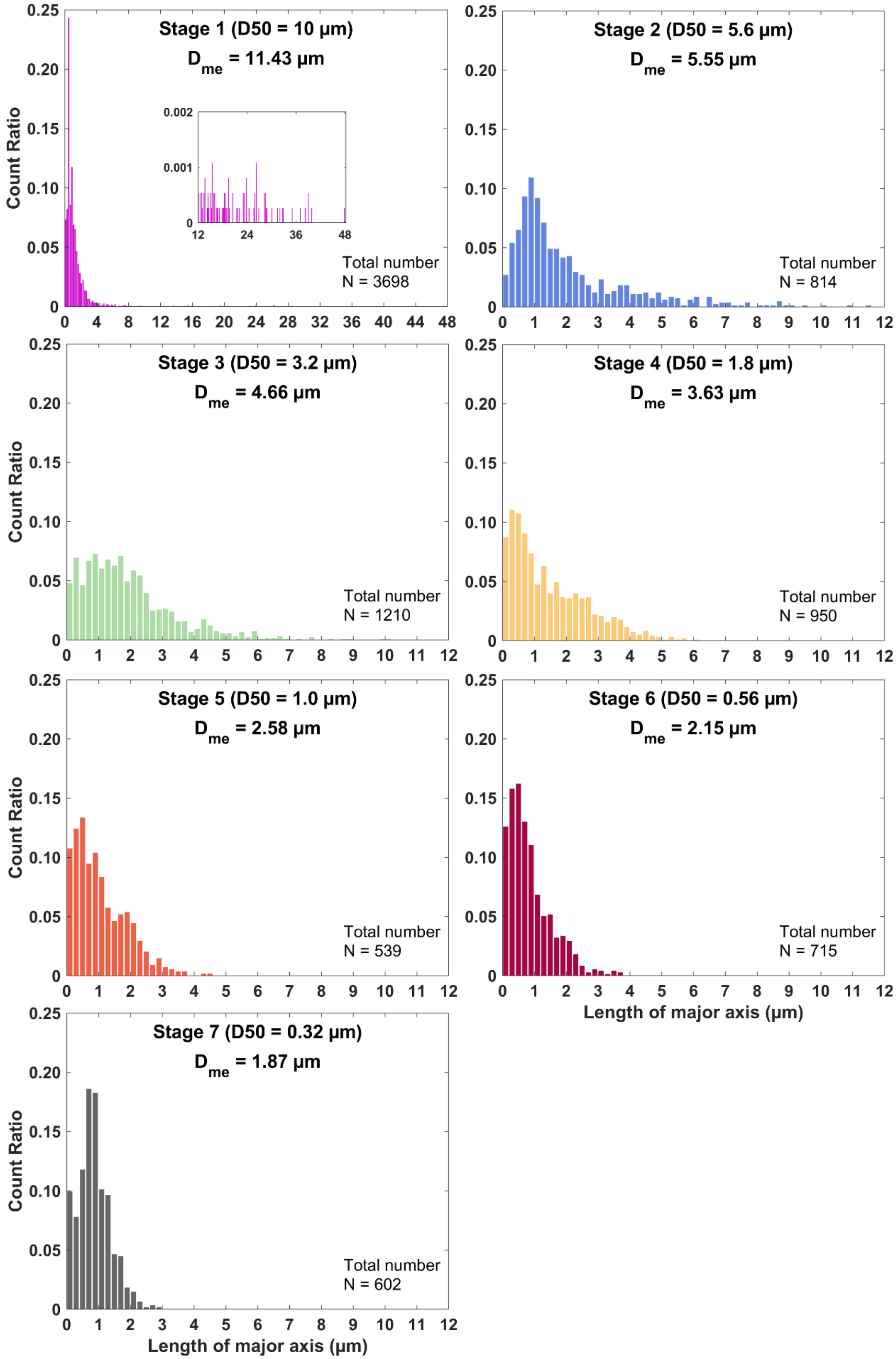

Figure S2. Particle size distribution in terms of major axis lengths of the seven investigated impactor stages. The mass-equivalent diameter (D_{me}) for each stage is indicated in the respective panel. Note the different x-axis scales.

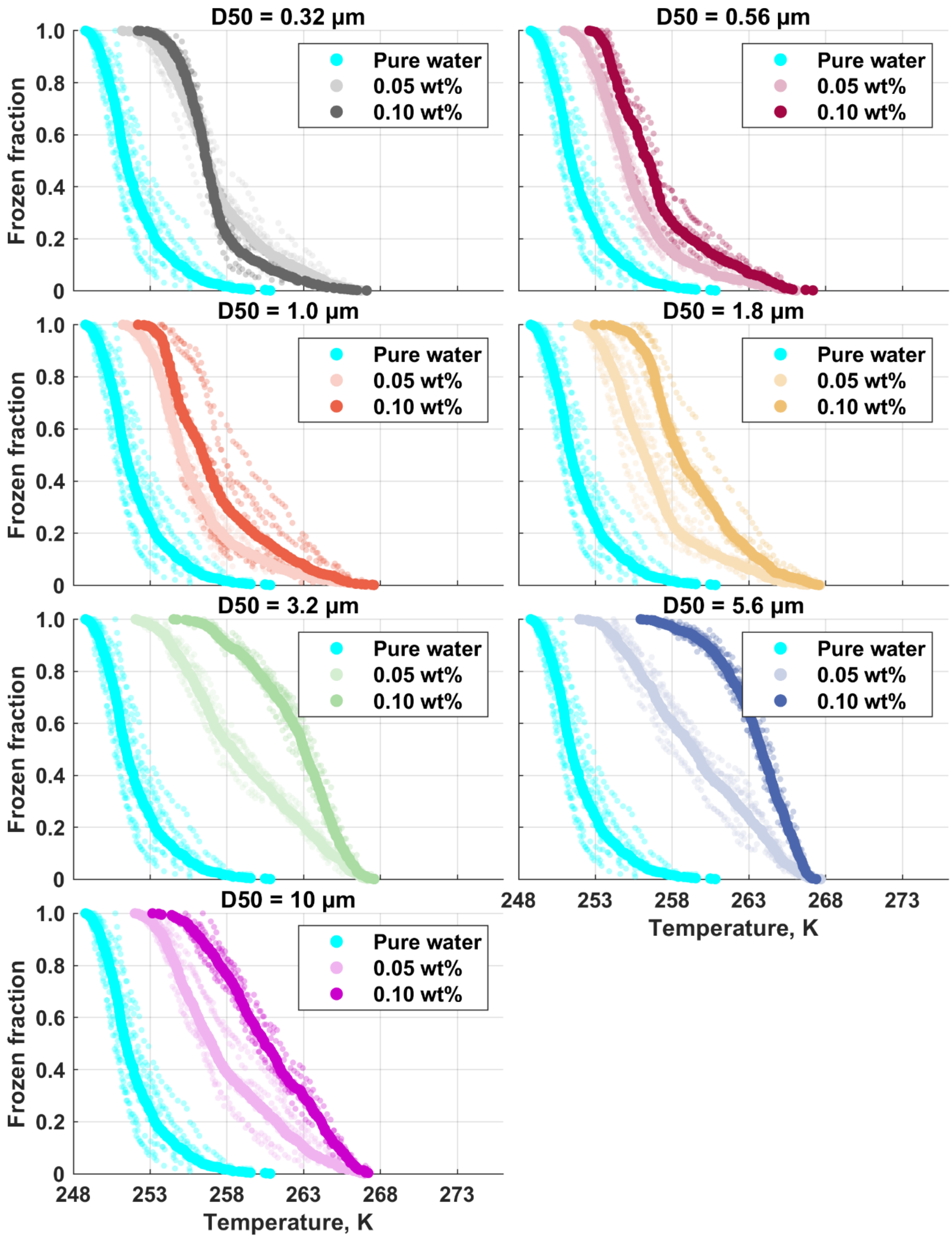

Figure S3. Frozen fractions of 50 µL **DRINCZ** droplets containing **0.05wt%** and **0.1wt%** KGa-1b particles from the seven investigated impactor stages (D50 = 0.32, 0.56, 1.0, 1.8, 3.2, 5.6, and 10 µm).

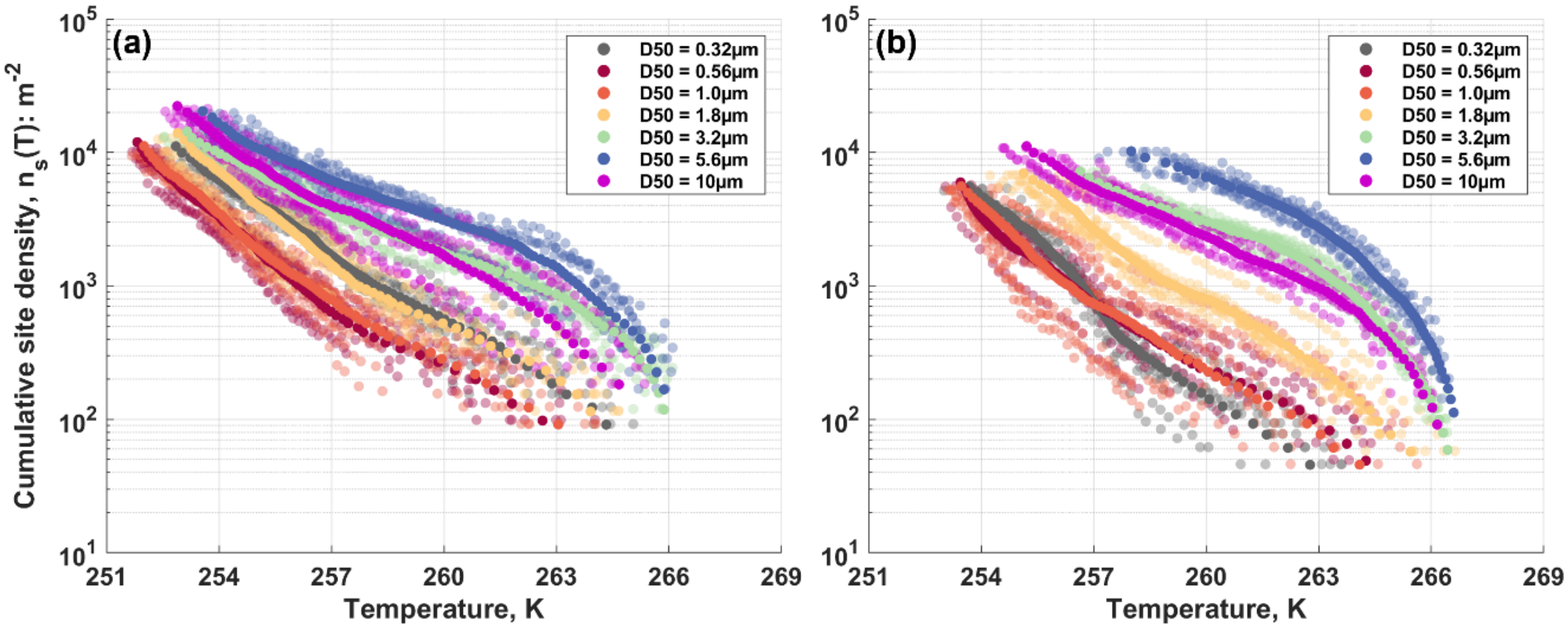

Figure S4. INAS densities derived from DRINCZ experiments with concentrations of **(a) 0.05 wt%** and **(b) 0.1 wt%** KGa-1b particles collected from the investigated impactor stages (D50 = 0.32, 0.56, 1.0, 1.8, 3.2, 5.6, and 10 µm). Averaged INAS densities are shown in deeper shades, while individual datasets are displayed with higher transparency.

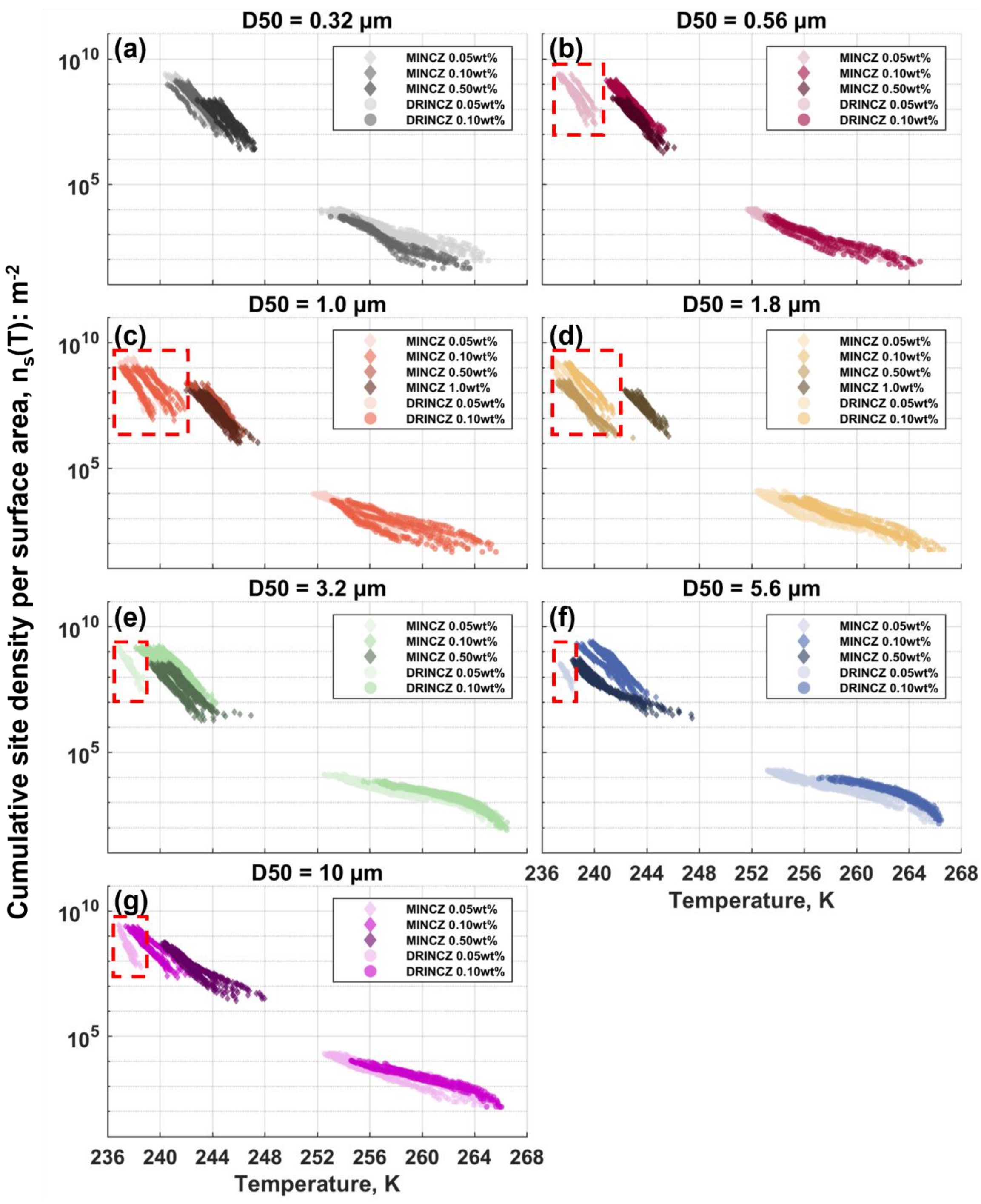

Figure S5. INAS densities derived from MINCZ and DRINCZ experiments performed with KGa-1b particles for the investigated impactor stages (D50 = 0.32, 0.56, 1.0, 1.8, 3.2, 5.6, and 10 µm) for all investigated concentrations. INAS densities enclosed by red dashed rectangles deviate from the general trend and are excluded from the simulation of the ice-nucleating particle fraction.

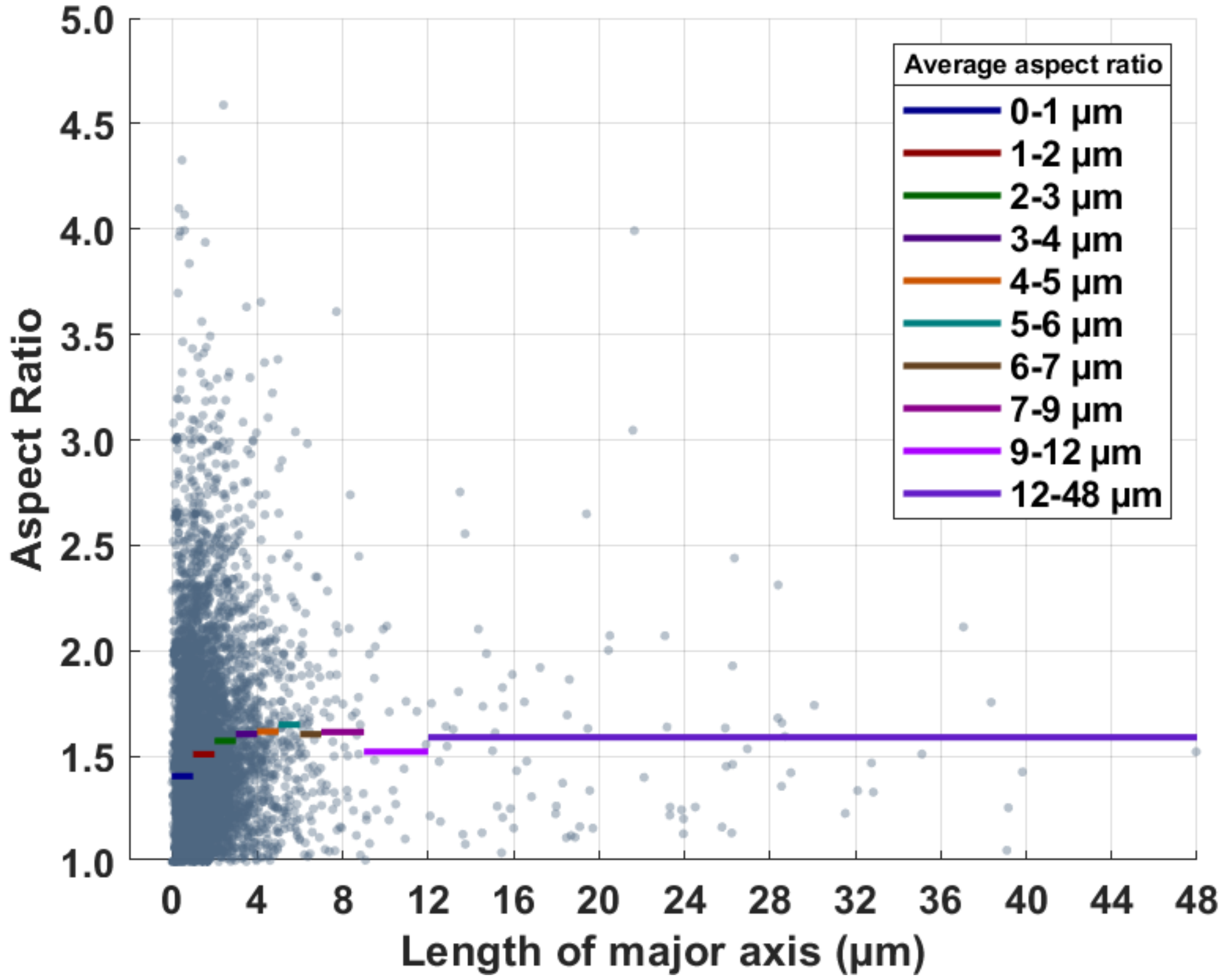

Figure S6. Apparent aspect ratios of the particles as a function of their major axis length (gray dots) and average aspect ratio for each size class (horizontal lines, see legend for size classes).

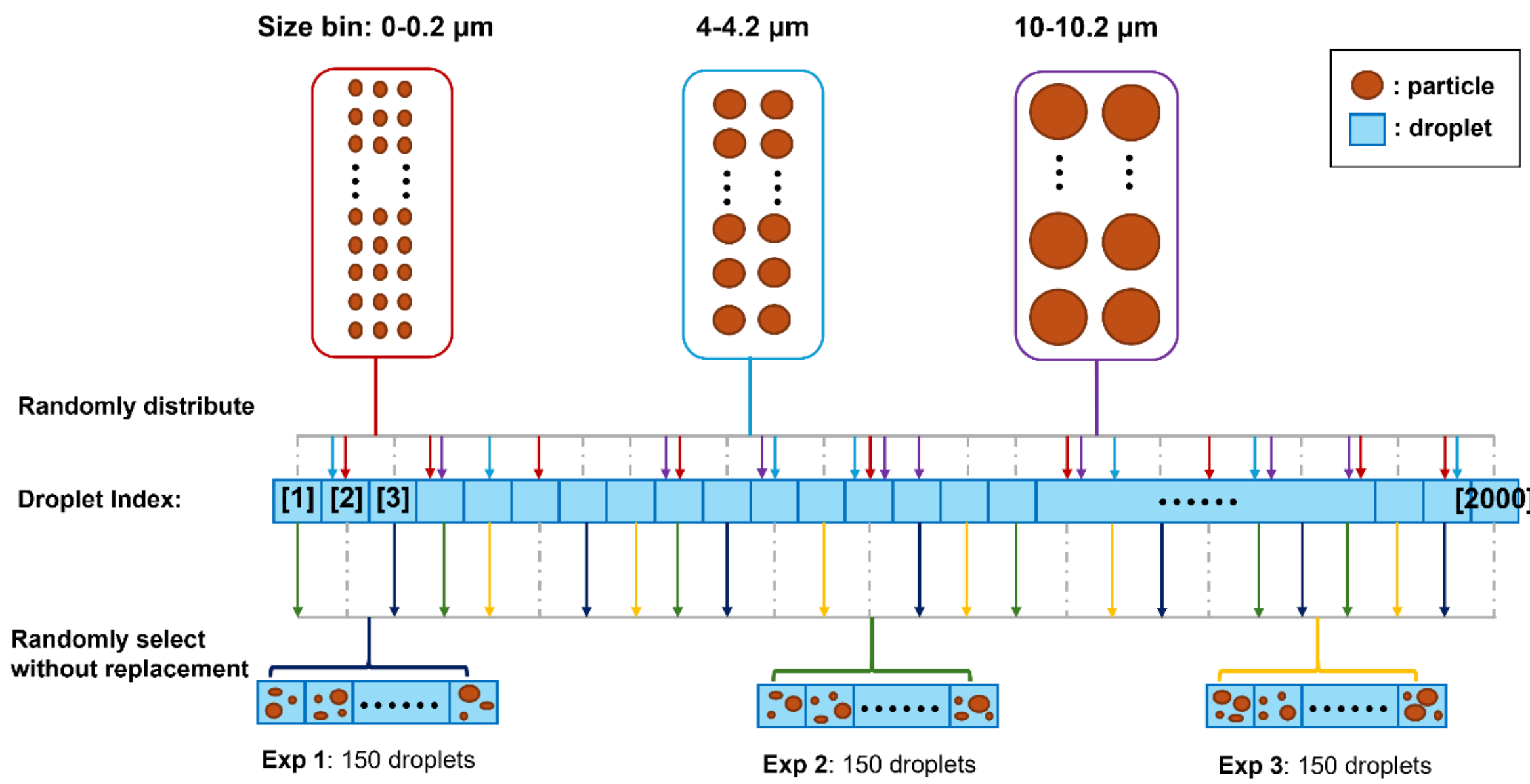

Figure S7. Schematic of the Monte Carlo simulation used to distribute the kaolinite particles randomly across the droplets in MINCZ experiments. The bulk suspension was represented by 2000 indexed droplets. N_i particles from each size bin i were randomly distributed across the droplets, and 150 randomly selected droplets were combined to a droplet population of a MINCZ experiment. This process was repeated twice to simulate a total of three independent experiments.

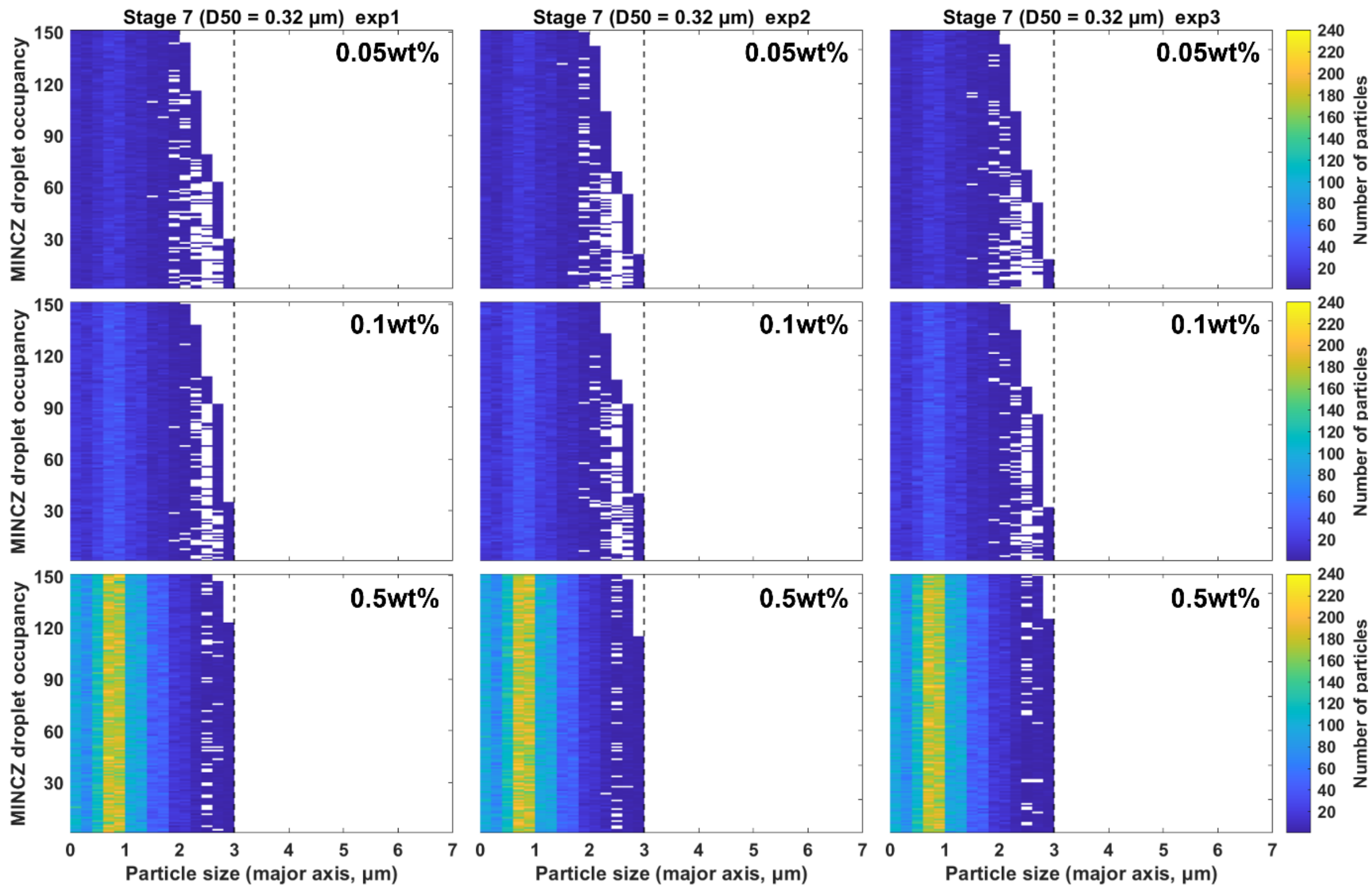

Figure S8. Particle size distributions in 150 individual MINCZ droplets at **0.05 wt%, 0.1 wt%**, and **0.5 wt%** from Monte Carlo simulations for **D50 = 0.32 μm**. For each concentration, three independent experiments were simulated (denoted **exp1**, **exp2** and **exp3**). Droplets are ordered with respect to the largest particle present in each droplet (y-axis). Note that the vertical dashed line at D = 3.0 μm marks the largest particle detected in SEM images of this stage.

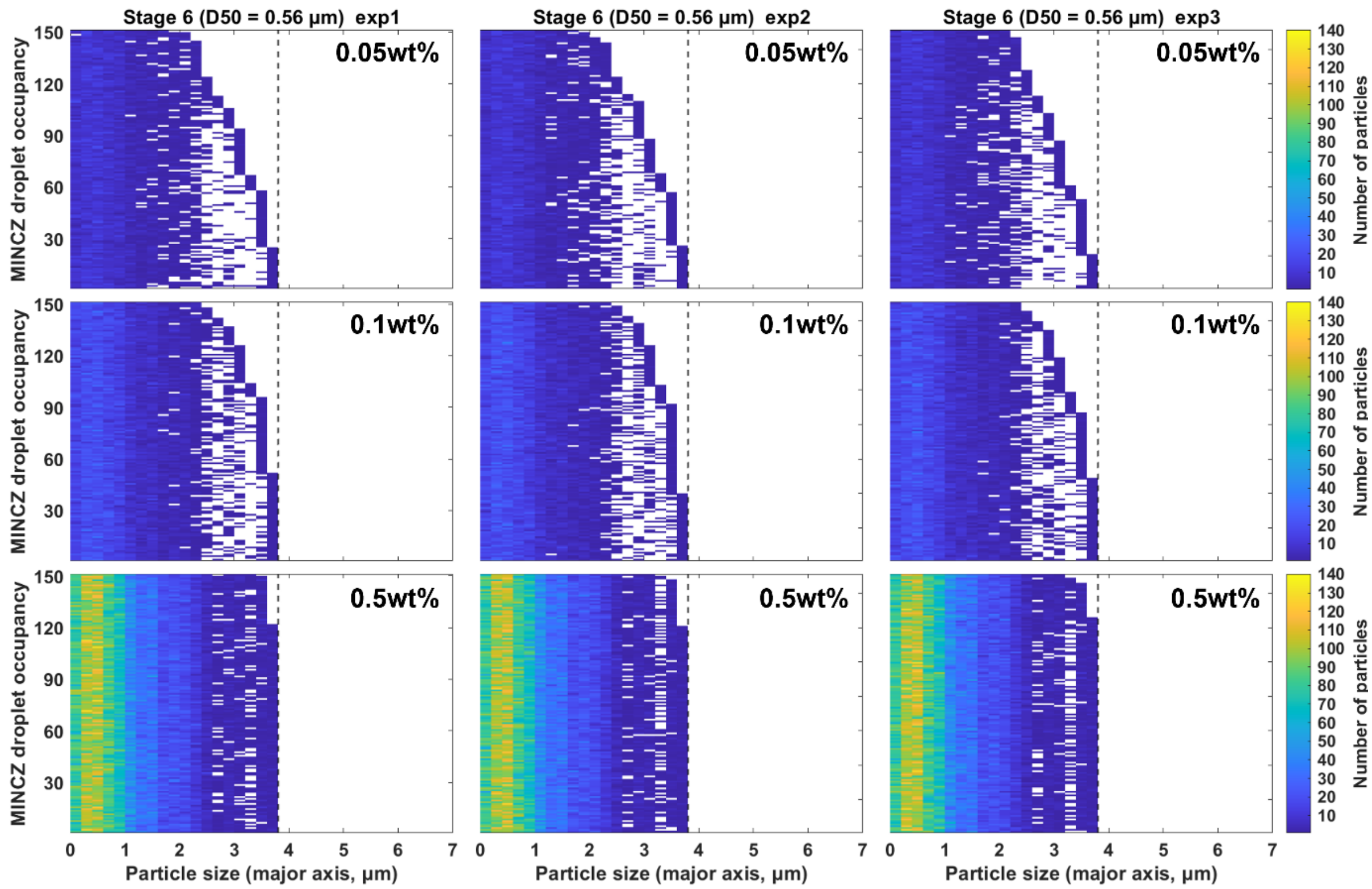

Figure S9. Particle size distributions in 150 individual MINCZ droplets at **0.05 wt%, 0.1 wt%**, and **0.5 wt%** from Monte Carlo simulations for **D50 = 0.56 µm**. For each concentration, three independent experiments were simulated (denoted **exp1**, **exp2** and **exp3**). Droplets are ordered with respect to the largest particle present in each droplet (y-axis). Note that the vertical dashed line at D = 3.8 µm marks the largest particle detected in SEM images of this stage.

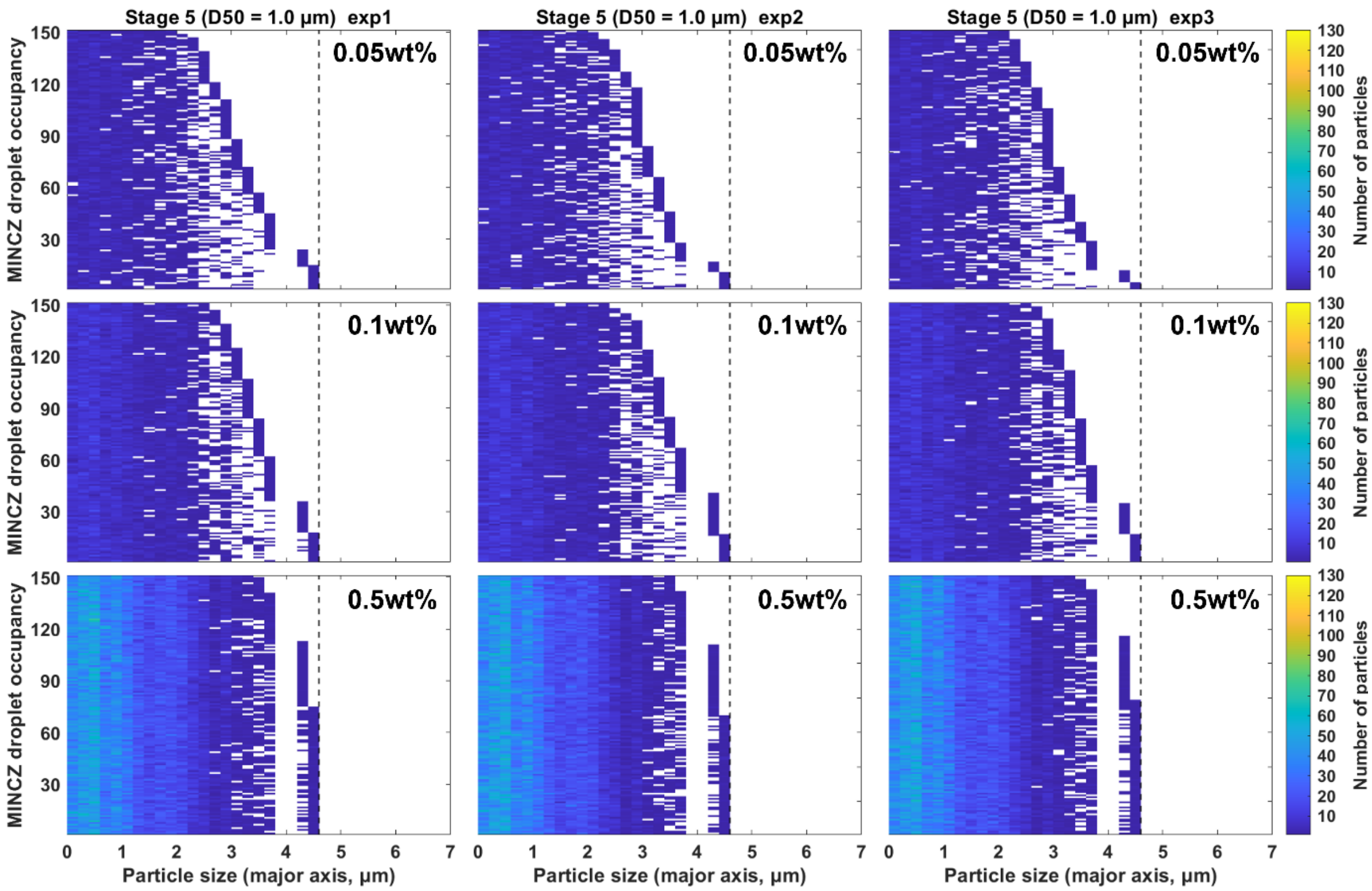

Figure S10. Particle size distributions in 150 individual MINCZ droplets at **0.05 wt%, 0.1 wt%**, and **0.5 wt%** from Monte Carlo simulations for **D50 = 1.0 µm**. For each concentration, three independent experiments were simulated (denoted **exp1**, **exp2** and **exp3**). Droplets are ordered with respect to the largest particle present in each droplet (y-axis). Note that the vertical dashed line at D = 4.6 µm marks the largest particle detected in SEM images of this stage.

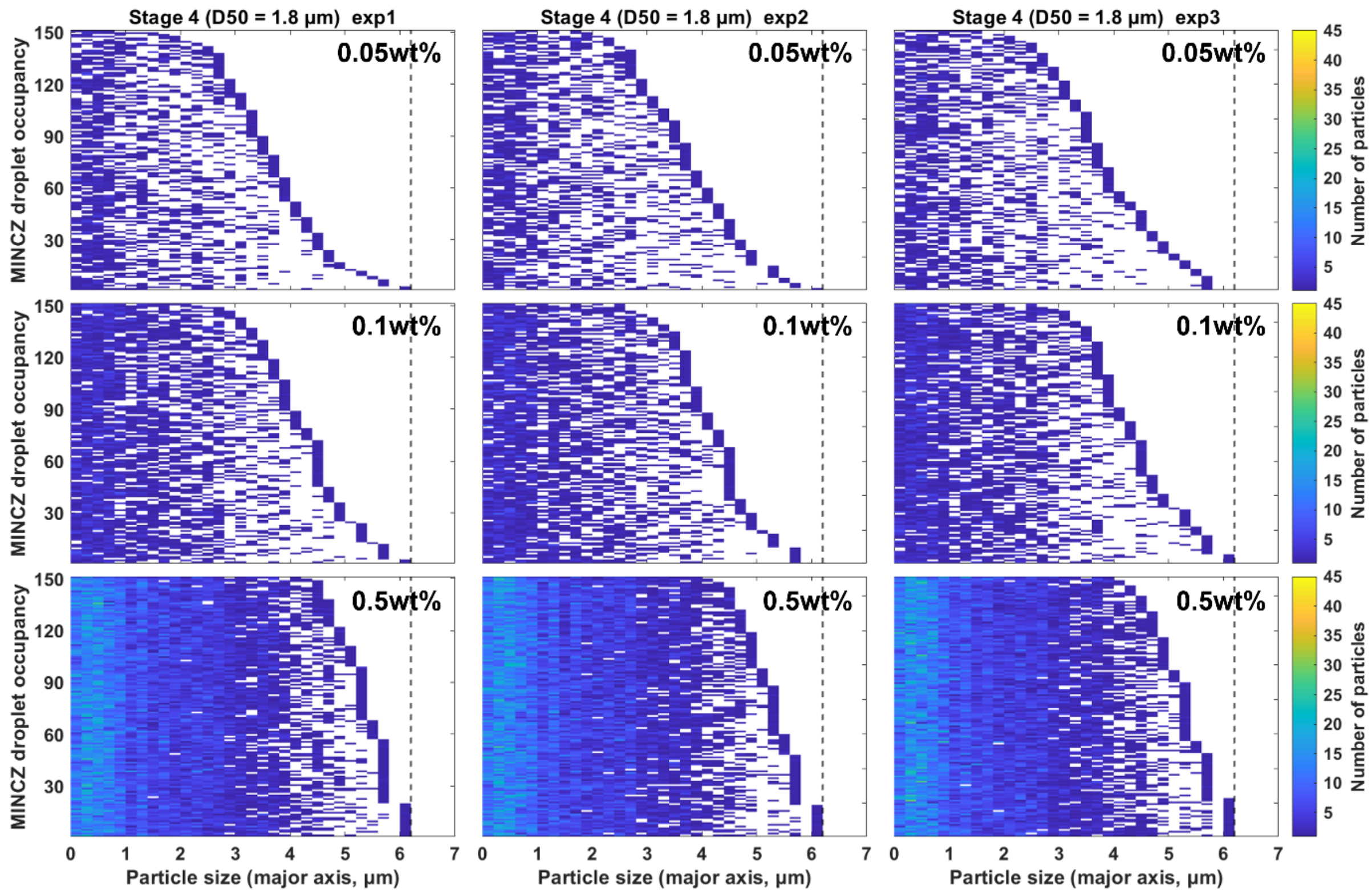

Figure S11. Particle size distributions in 150 individual MINCZ droplets at **0.05 wt%, 0.1 wt%**, and **0.5 wt%** from Monte Carlo simulations for **D50 = 1.8 µm**. For each concentration, three independent experiments were simulated (denoted **exp1**, **exp2** and **exp3**). Droplets are ordered with respect to the largest particle present in each droplet (y-axis). Note that the vertical dashed line at D = 6.2 µm marks the largest particle detected in SEM images of this stage.

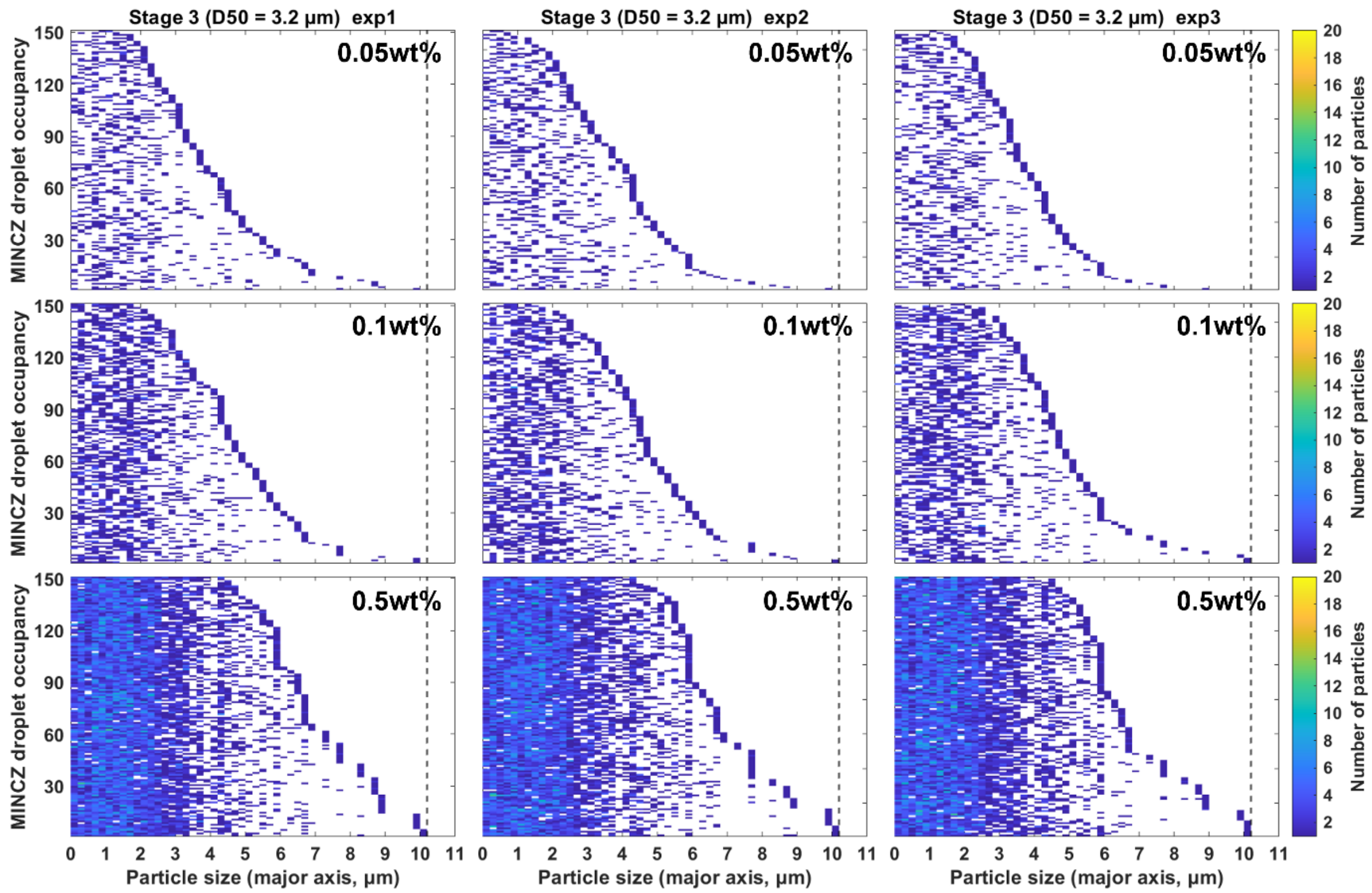

Figure S12. Particle size distributions in 150 individual MINCZ droplets at **0.05 wt%, 0.1 wt%**, and **0.5 wt%** from Monte Carlo simulations for **D50 = 3.2 μm**. For each concentration, three independent experiments were simulated (denoted **exp1**, **exp2** and **exp3**). Droplets are ordered with respect to the largest particle present in each droplet (y-axis). Note that the vertical dashed line at D = 10.2 μm marks the largest particle detected in SEM images of this stage.

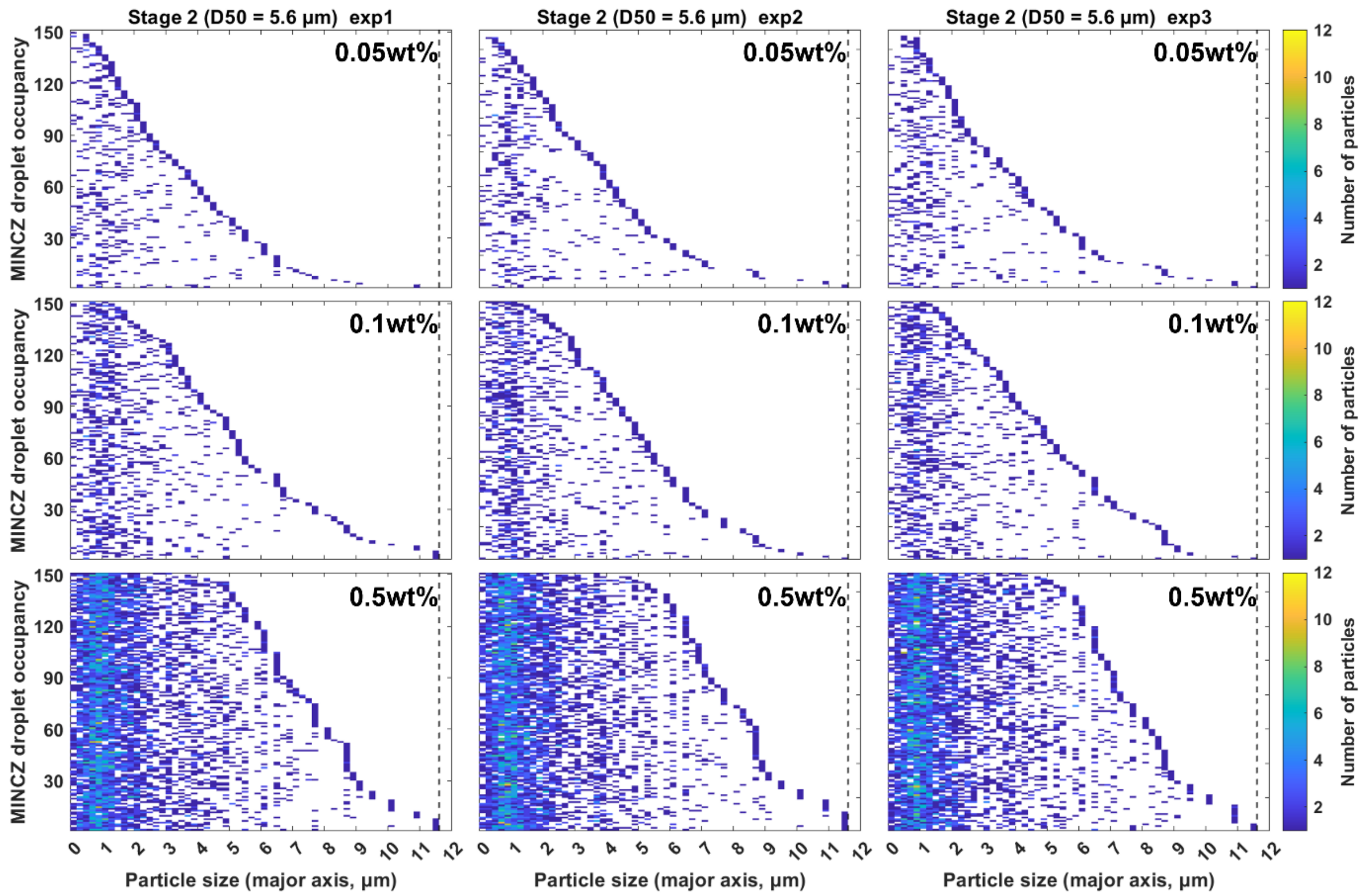

Figure S13. Particle size distributions in 150 individual MINCZ droplets at **0.05 wt%, 0.1 wt%**, and **0.5 wt%** from Monte Carlo simulations for **D50 = 5.6 µm**. For each concentration, three independent experiments were simulated (denoted **exp1**, **exp2** and **exp3**). Droplets are ordered with respect to the largest particle present in each droplet (y-axis). Note that the vertical dashed line at D = 11.6 µm marks the largest particle detected in SEM images of this stage.

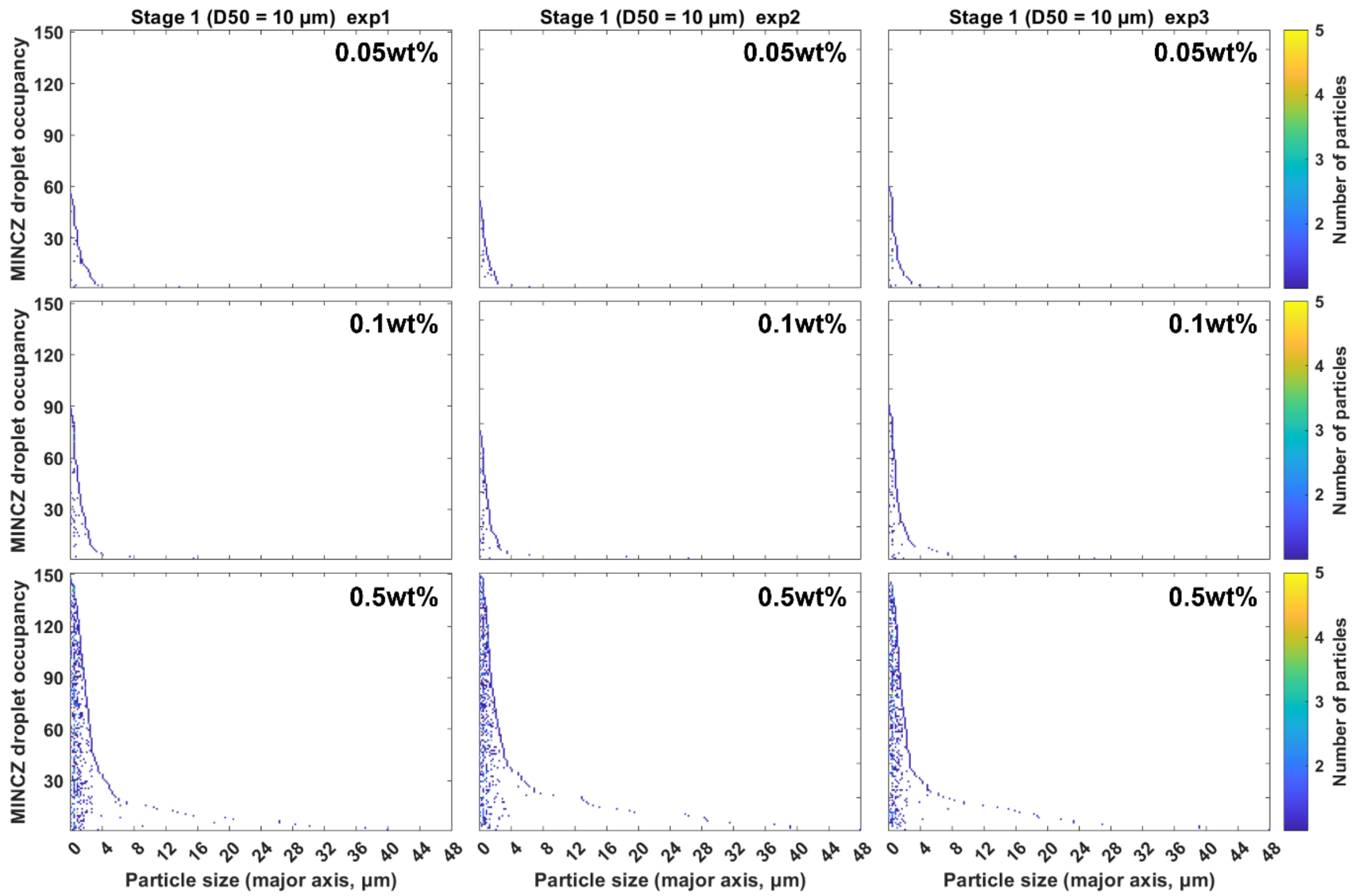

Figure S14. Particle size distributions in 150 individual MINCZ droplets at **0.05 wt%, 0.1 wt%**, and **0.5 wt%** from Monte Carlo simulations for **D50 = 10 µm**. For each concentration, three independent experiments were simulated (denoted **exp1**, **exp2** and **exp3**). Droplets are ordered with respect to the largest particle present in each droplet (y-axis). Note that the end of the x-axis (D = 48 µm*)* marks the largest particle detected in SEM images of this stage.

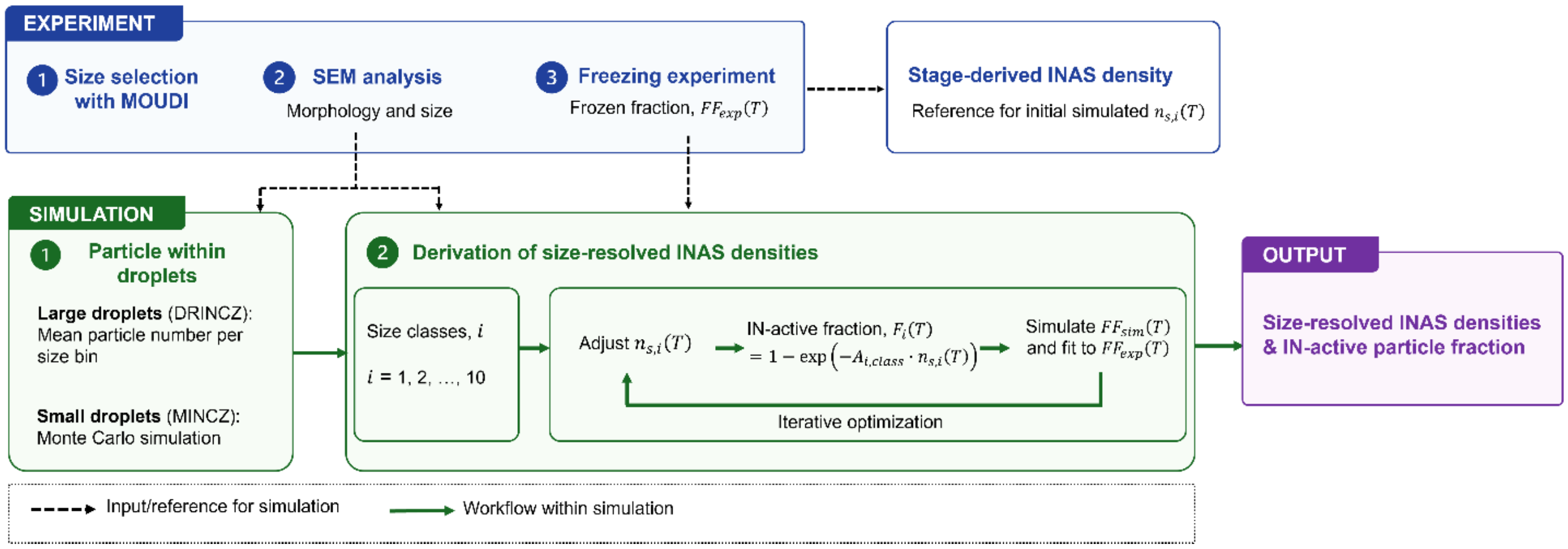

Figure S15. Experimental and simulation framework for deriving size-resolved INAS densities ($n_{s,i}(T)$) from freezing measurements. Aerosol particles were size segregated onto impactor stages depending on aerodynamic cut-off diameters (D50) and characterized by SEM imaging to obtain particle morphologies and size distributions. Freezing experiments were conducted for particles collected on each stage to determine frozen fractions and derive stage-based INAS densities. The stage-derived INAS densities were used as initial guess for simulated size-resolved $n_{s,i}(T)$. Size-bin-resolved particle numbers within droplets were estimated separately for DRINCZ and MINCZ experiments using mean particle-number calculations and Monte Carlo simulations, respectively. The particles per size bin were merged into the 10 size classes for the FF simulation, and $n_{s,i}(T)$ was iteratively optimized by calculating IN-active fractions, simulating frozen fractions, and minimizing the difference between simulated and experimental frozen fractions.

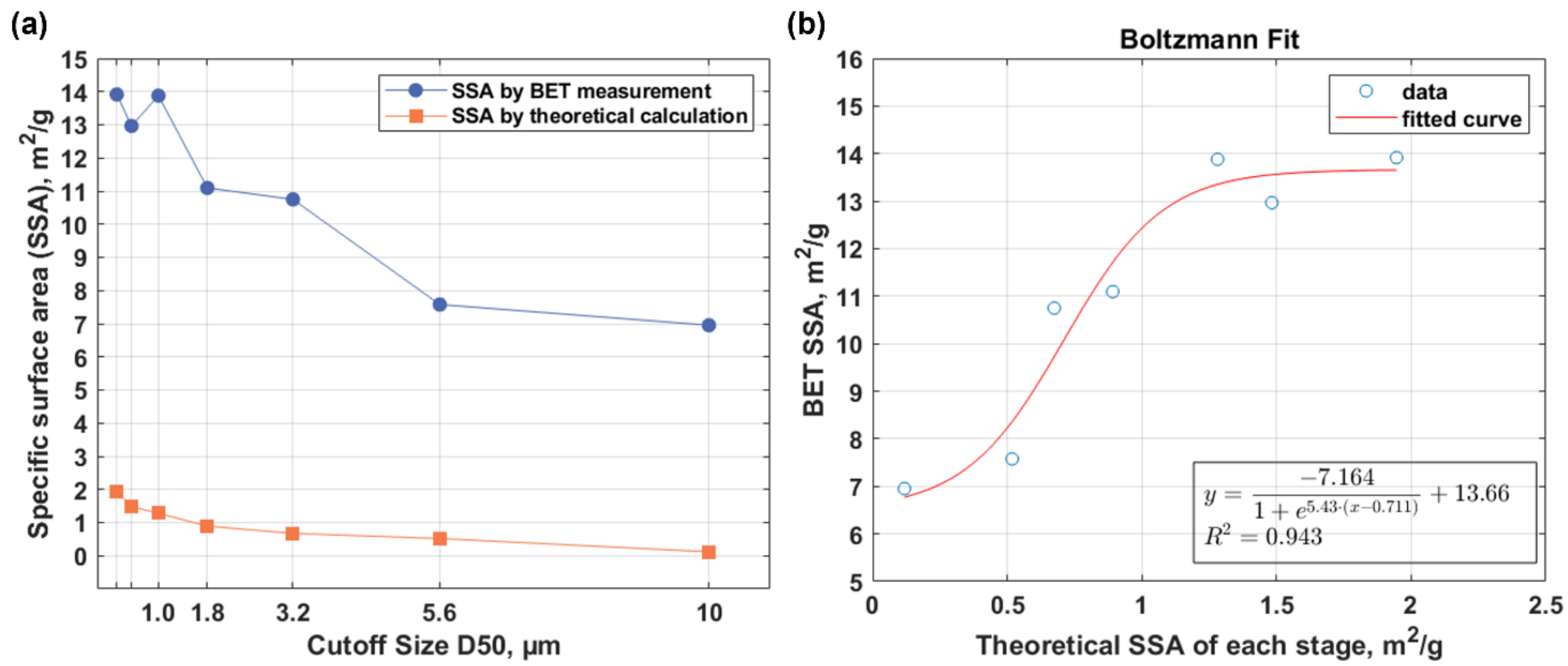

Figure S16. (a) Comparison of theoretical specific surface areas (SSA_{th}) calculated assuming spherical particles, with those measured by BET (SSA_{BET}); (b) Relationship between SSA_{th} and SSA_{BET} fitted using a four-parameter Boltzmann function.

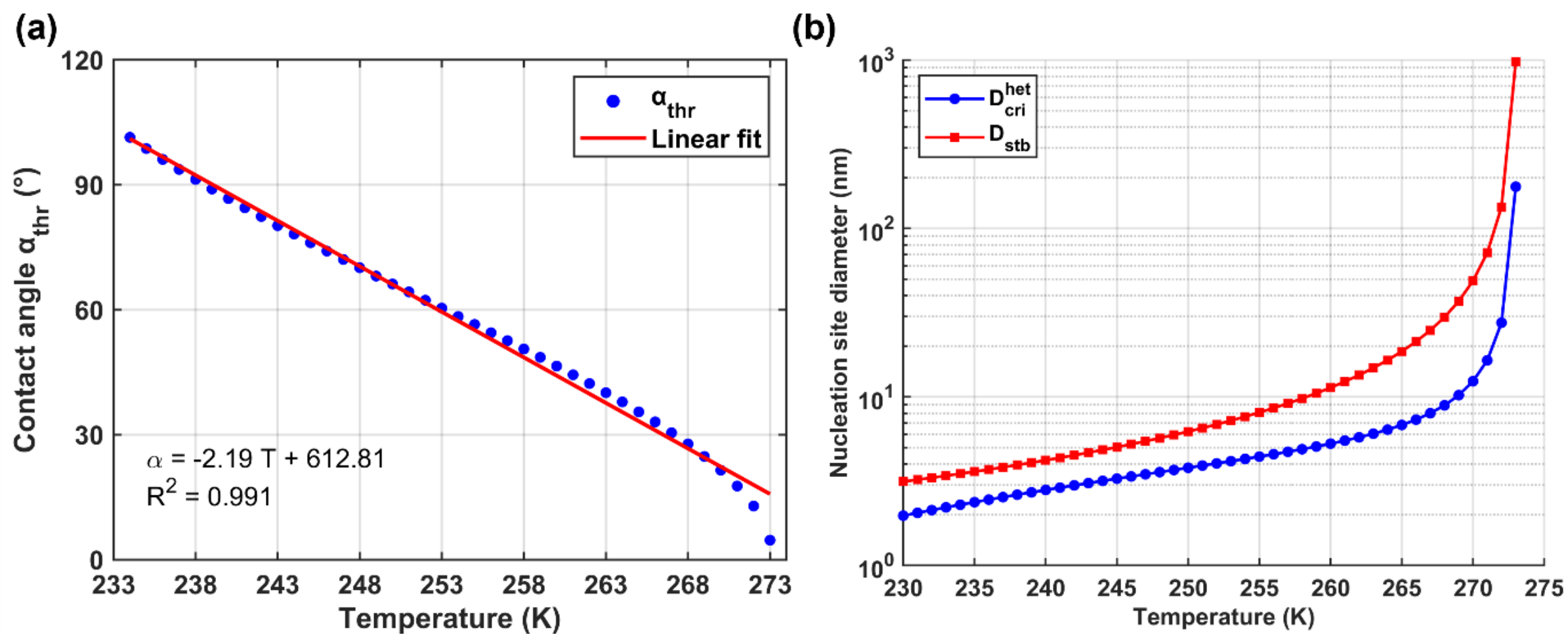

Figure S17. (a) Linear fit between temperature and threshold contact angle (α_{thr}); (b) Diameters of critical (D_{cri}^{het}) and stable (D_{stb}) ice embryos as a function of temperature.

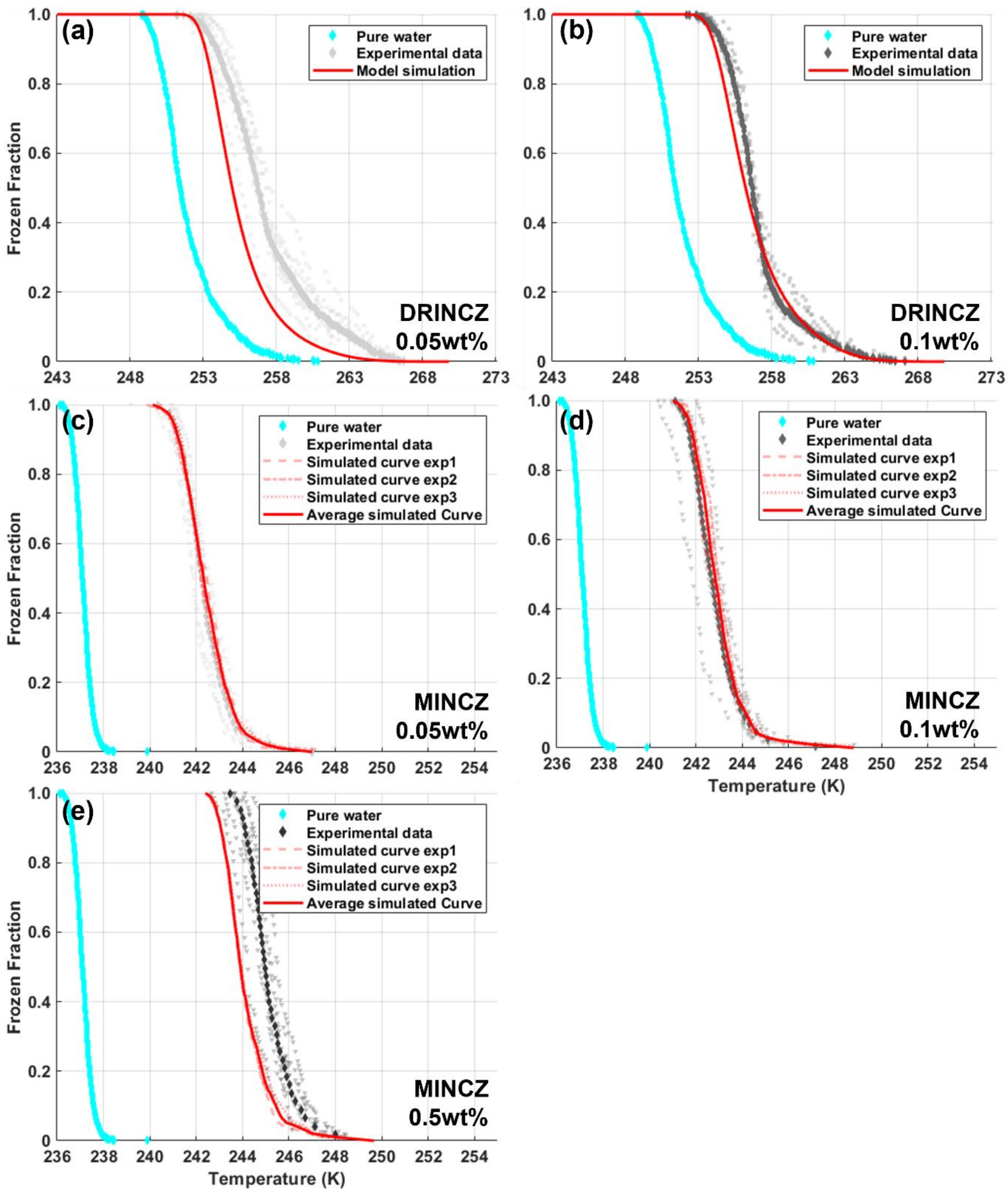

Figure S18. Comparison between measured and simulated frozen fractions for **D50 = 0.32 μm**. Simulated DRINCZ frozen fractions (panels a and b) were generated using a probabilistic model, resulting in smooth curves. For simulated MINCZ curves (c–e), droplet freezing temperatures were determined by the highest freezing temperature of contained particles. Particle size distributions and freezing temperatures were derived from Monte Carlo simulations, producing comparatively rougher curves.

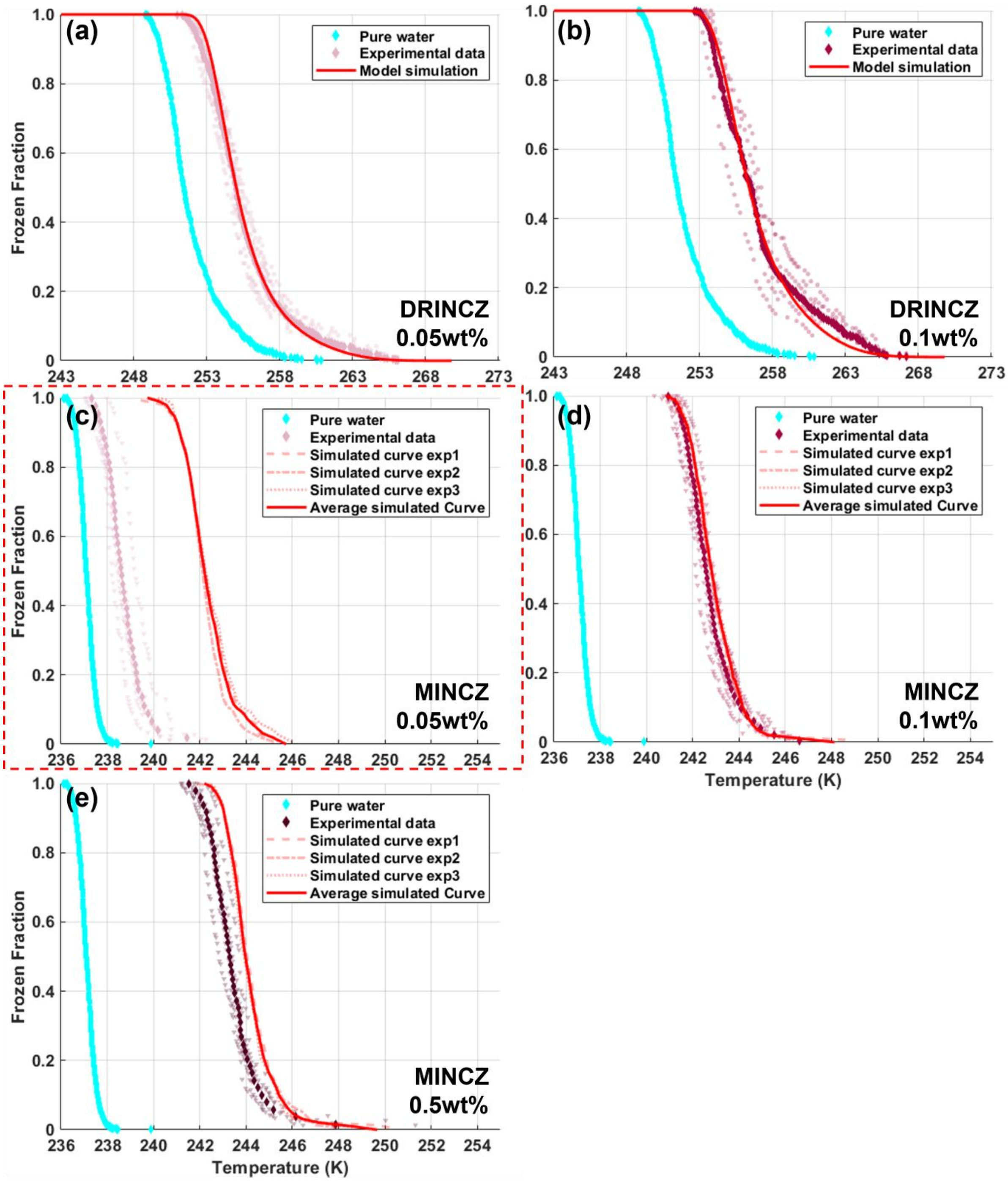

Figure S19. Comparison between measured and simulated frozen fractions for **D50 = 0.56 µm**. Simulated DRINCZ frozen fractions (panels a and b) were generated using a probabilistic model, resulting in smooth curves. For simulated MINCZ curves (c–e), droplet freezing temperatures were determined by the highest freezing temperature of contained particles. Particle size distributions and freezing temperatures were derived from Monte Carlo simulations, producing comparatively rougher curves. The red dashed rectangle denotes the experiments that were excluded from the simulation of the size-dependent ice-nucleating particle fraction.

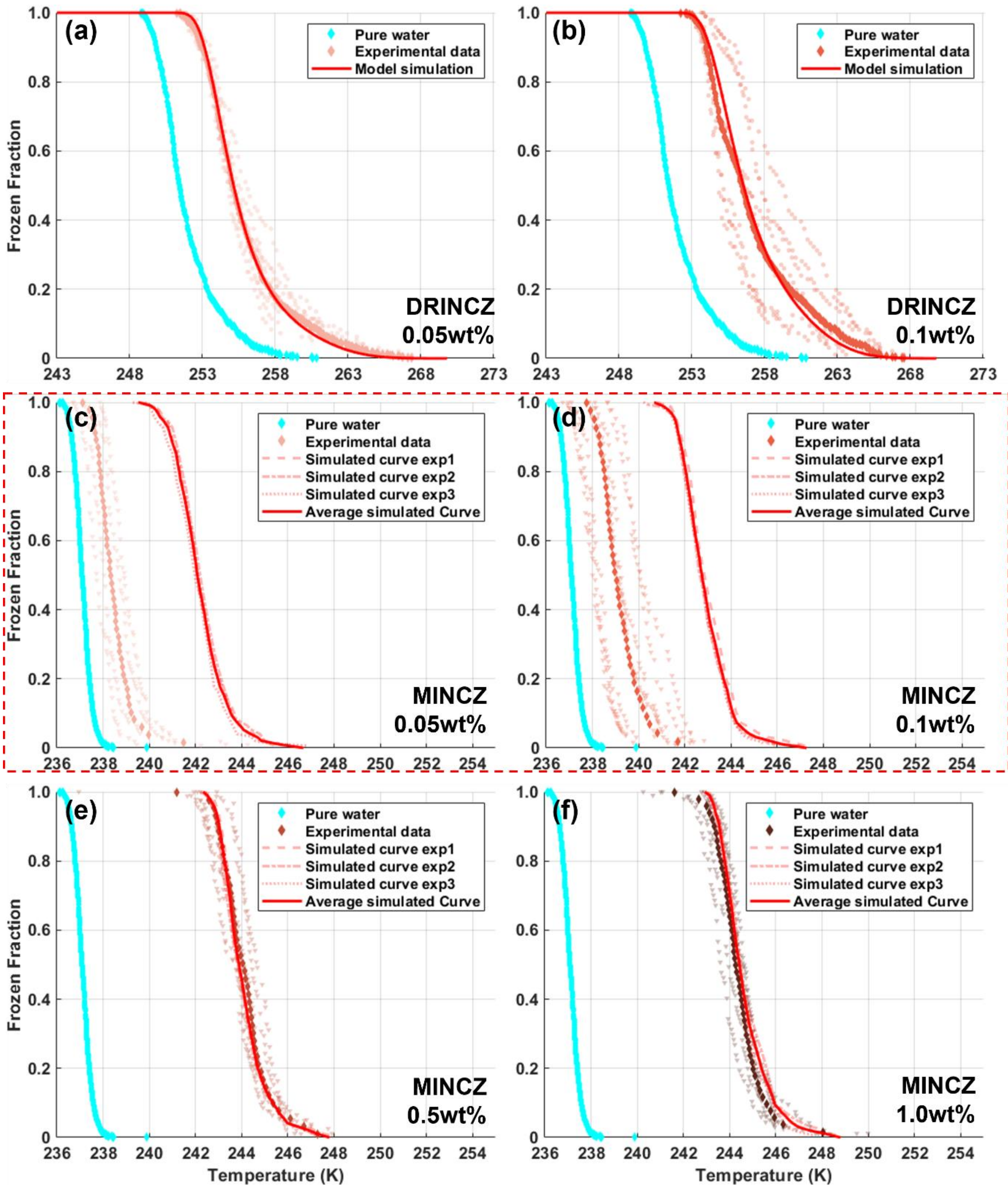

Figure S20. Comparison between measured and simulated frozen fractions for **D50 = 1.0 µm**. Simulated DRINCZ frozen fractions (panels a and b) were generated using a probabilistic model, resulting in smooth curves. For simulated MINCZ curves (c–e), droplet freezing temperatures were determined by the highest freezing temperature of contained particles. Particle size distributions and freezing temperatures were derived from Monte Carlo simulations, producing comparatively rougher curves. The red dashed rectangle denotes the experiments that were excluded from the simulation of the size-dependent ice-nucleating particle fraction.

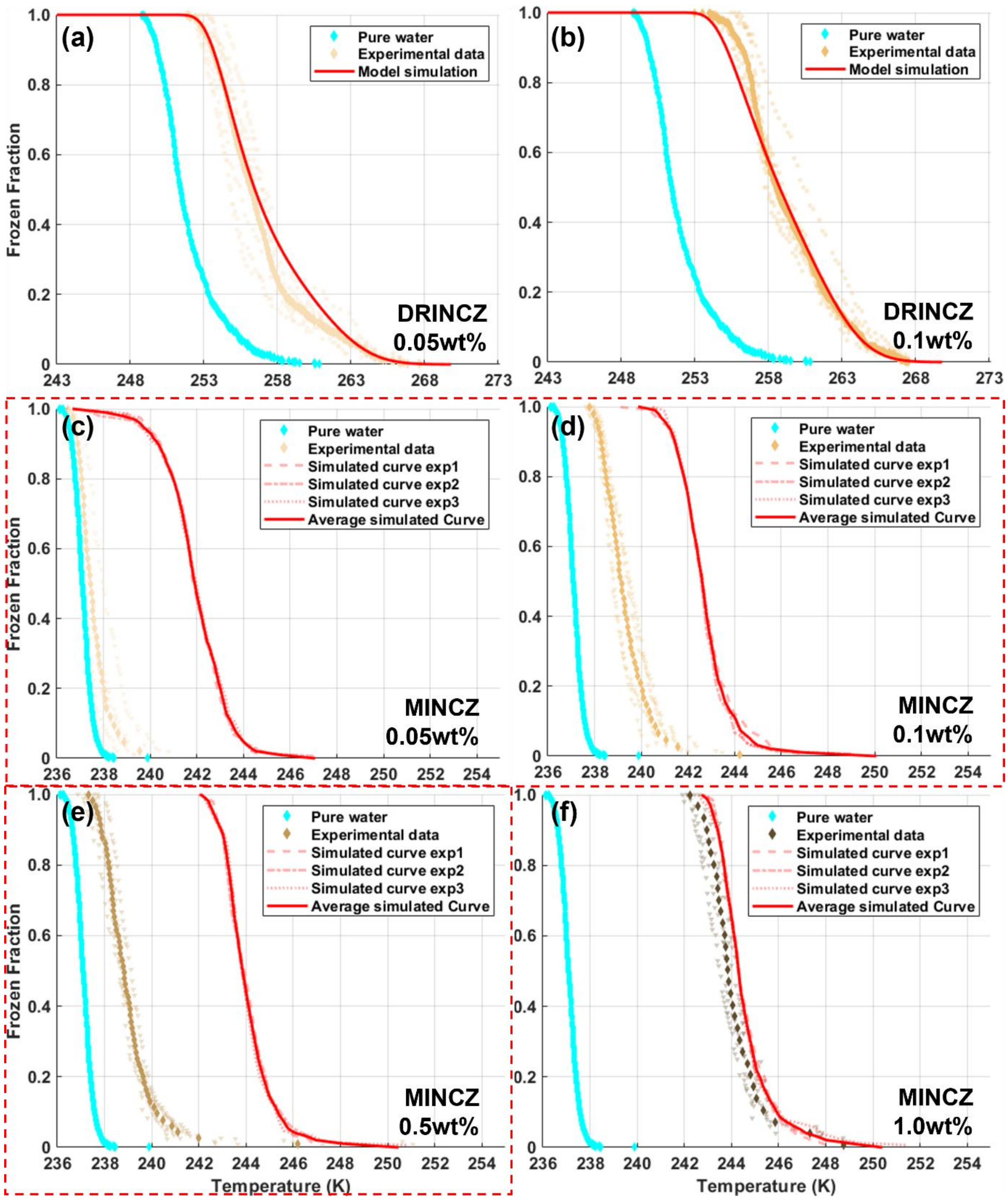

Figure S21. Comparison between measured and simulated frozen fractions for **D50 = 1.8 µm**. Simulated DRINCZ frozen fractions (panels a and b) were generated using a probabilistic model, resulting in smooth curves. For simulated MINCZ curves (c–e), droplet freezing temperatures were determined by the highest freezing temperature of contained particles. Particle size distributions and freezing temperatures were derived from Monte Carlo simulations, producing comparatively rougher curves. The red dashed rectangle denotes the experiments that were excluded from the simulation of the size-dependent ice-nucleating particle fraction.

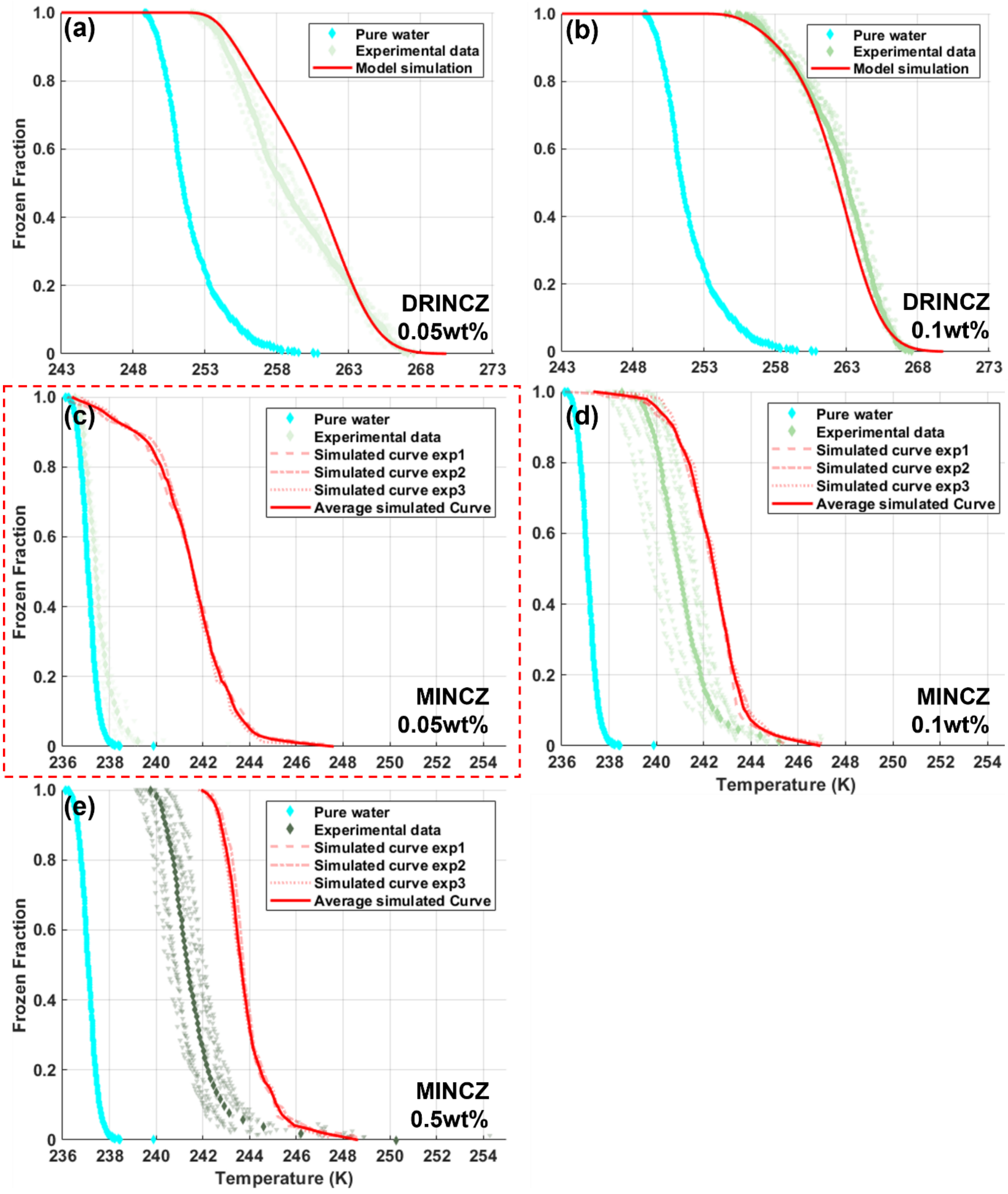

Figure S22. Comparison between measured and simulated frozen fractions for **D50 = 3.2 µm**. Simulated DRINCZ frozen fractions (panels a and b) were generated using a probabilistic model, resulting in smooth curves. For simulated MINCZ curves (c–e), droplet freezing temperatures were determined by the highest freezing temperature of contained particles. Particle size distributions and freezing temperatures were derived from Monte Carlo simulations, producing comparatively rougher curves. The red dashed rectangle denotes the experiments that were excluded from the simulation of the size-dependent ice-nucleating particle fraction.

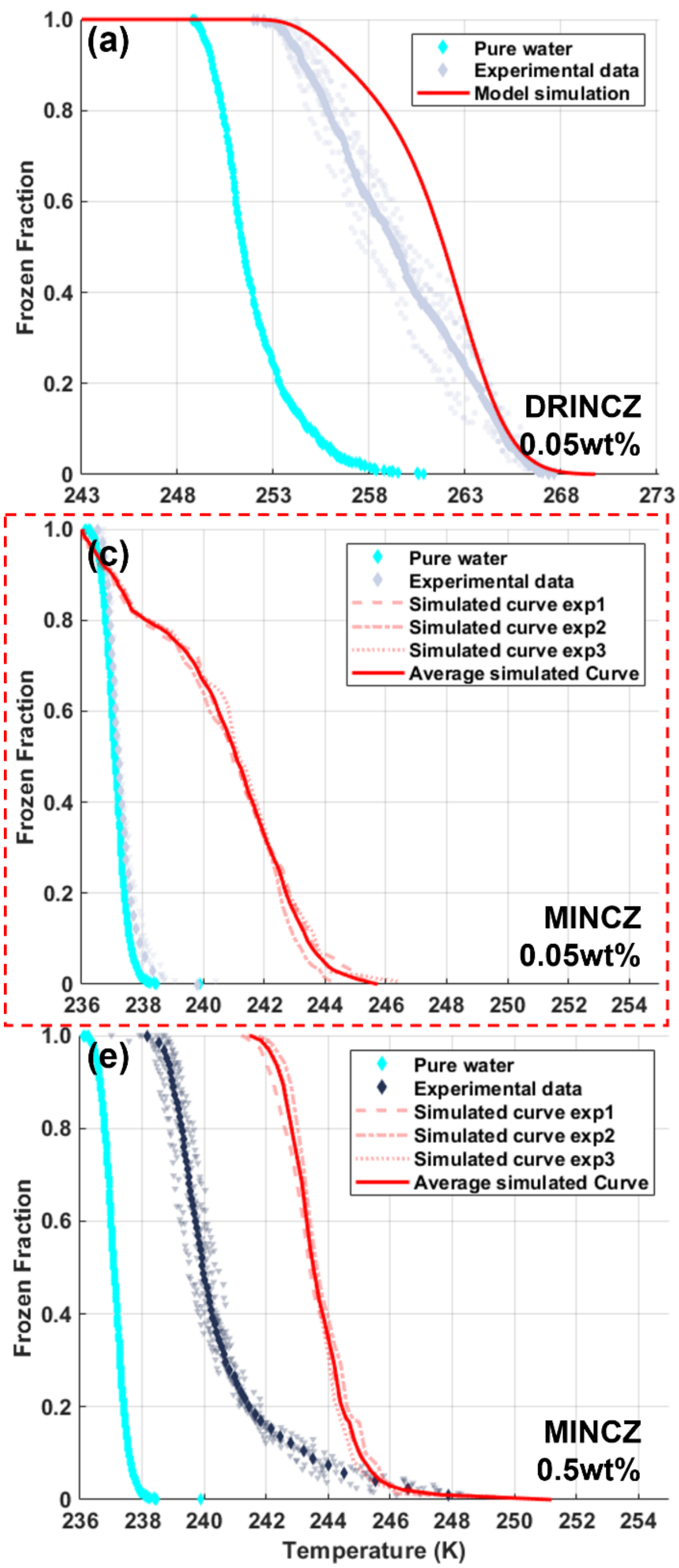

Figure S23. Comparison between measured and simulated frozen fractions for **D50 = 5.6 µm**. Simulated DRINCZ frozen fractions (panels a and b) were generated using a probabilistic model, resulting in smooth curves. For simulated MINCZ curves (c–e), droplet freezing temperatures were determined by the highest freezing temperature of contained particles. Particle size distributions and freezing temperatures were derived from Monte Carlo simulations, producing comparatively rougher curves. The red dashed rectangle denotes the experiment that was excluded from the simulation of the size-dependent ice-nucleating particle fraction.

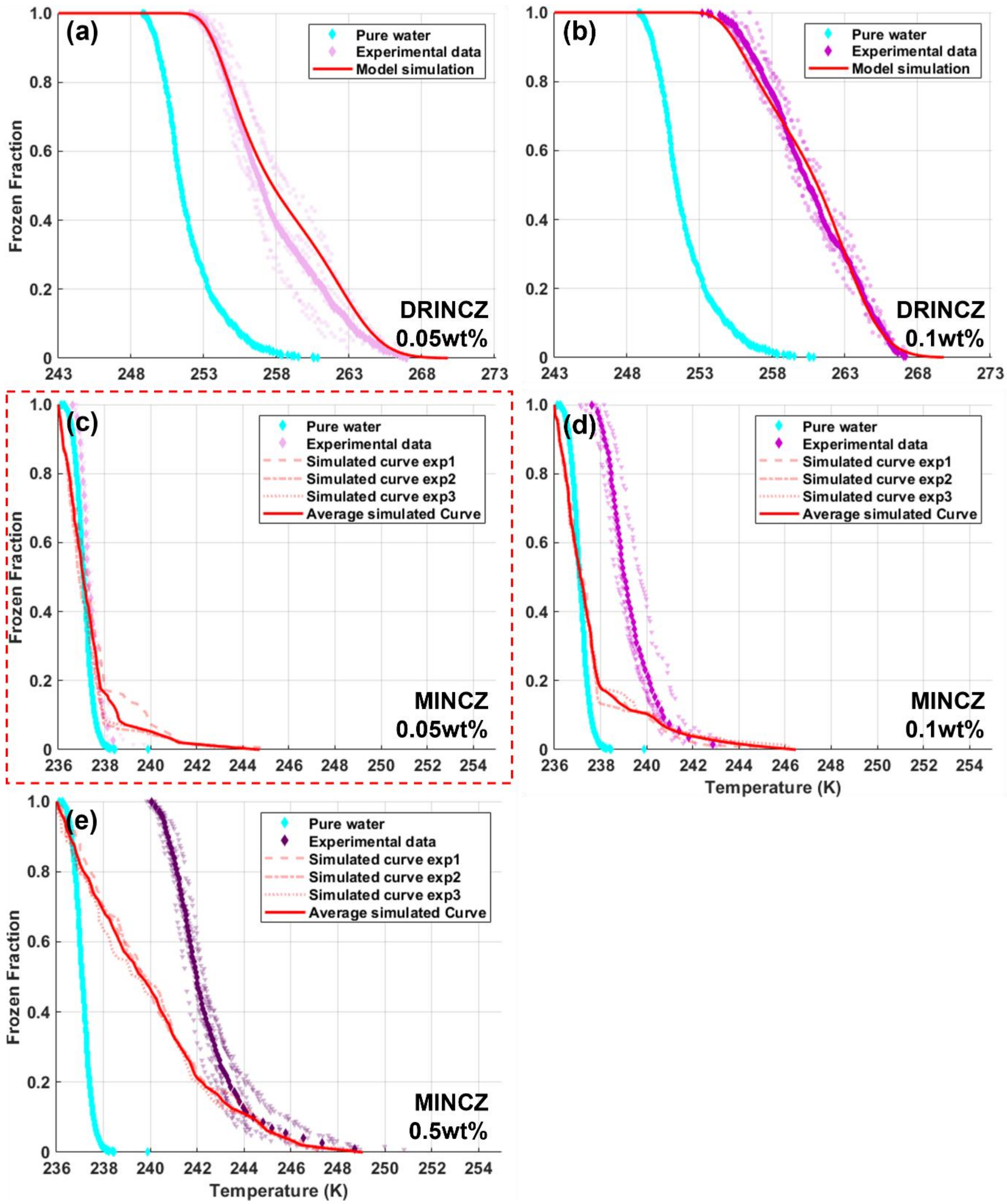

Figure S24. Comparison between measured and simulated frozen fractions for **D50 = 10 µm**. Simulated DRINCZ frozen fractions (panels a and b) were generated using a probabilistic model, resulting in smooth curves. For simulated MINCZ curves (c–e), droplet freezing temperatures were determined by the highest freezing temperature of contained particles. Particle size distributions and freezing temperatures were derived from Monte Carlo simulations, producing comparatively rougher curves. The red dashed rectangle denotes the experiment that was excluded from the simulation of the size-dependent ice-nucleating particle fraction.

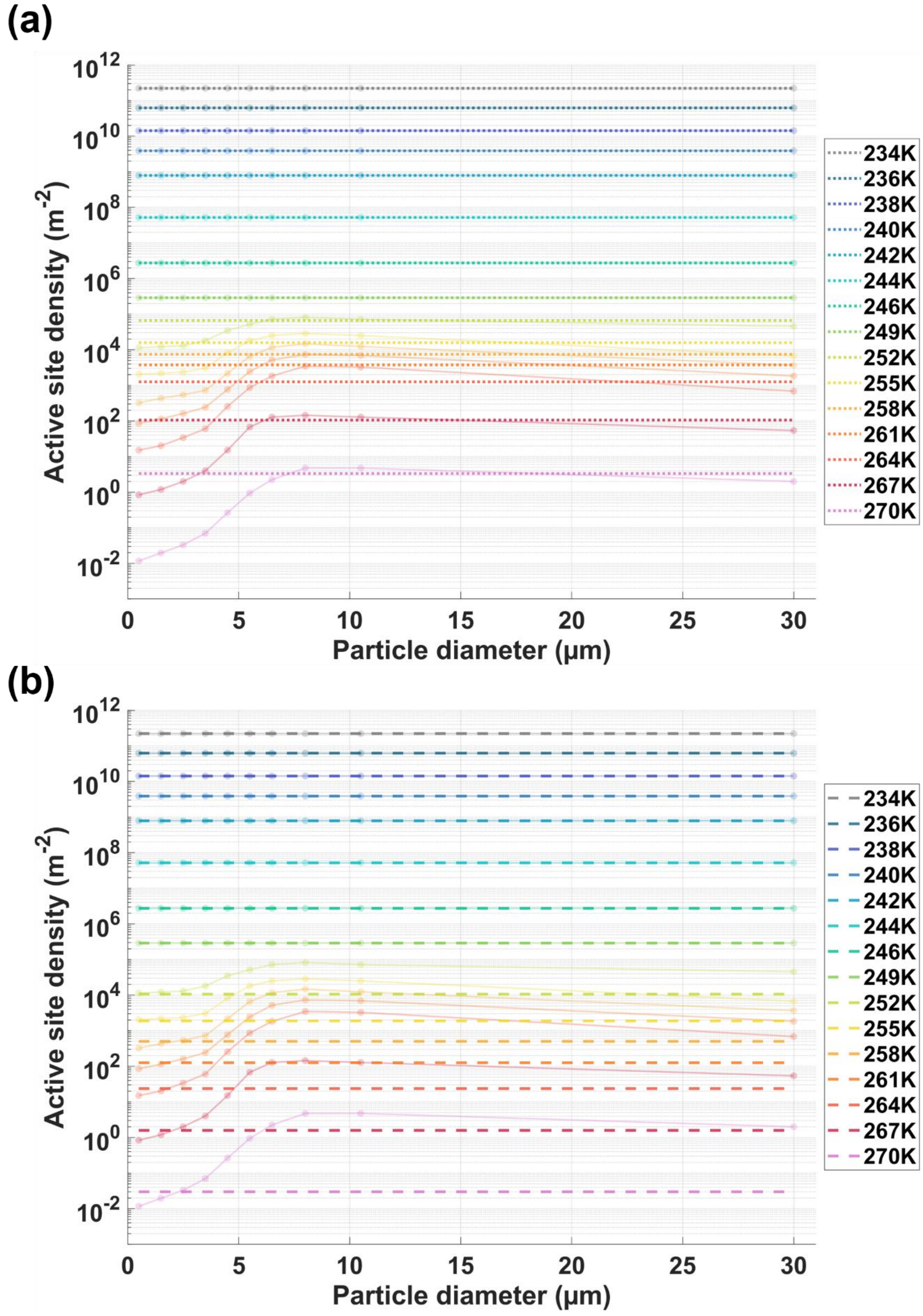

Figure S25. Constant INAS density values optimized to fit either (a) the highest DRINCZ frozen fraction (D50 = 5.6 µm) or (b) the lowest DRINCZ frozen fraction (D50 = 0.32 µm) well. The transparent solid line and markers indicate the best-fit INAS density that reproduces all frozen fraction curves.

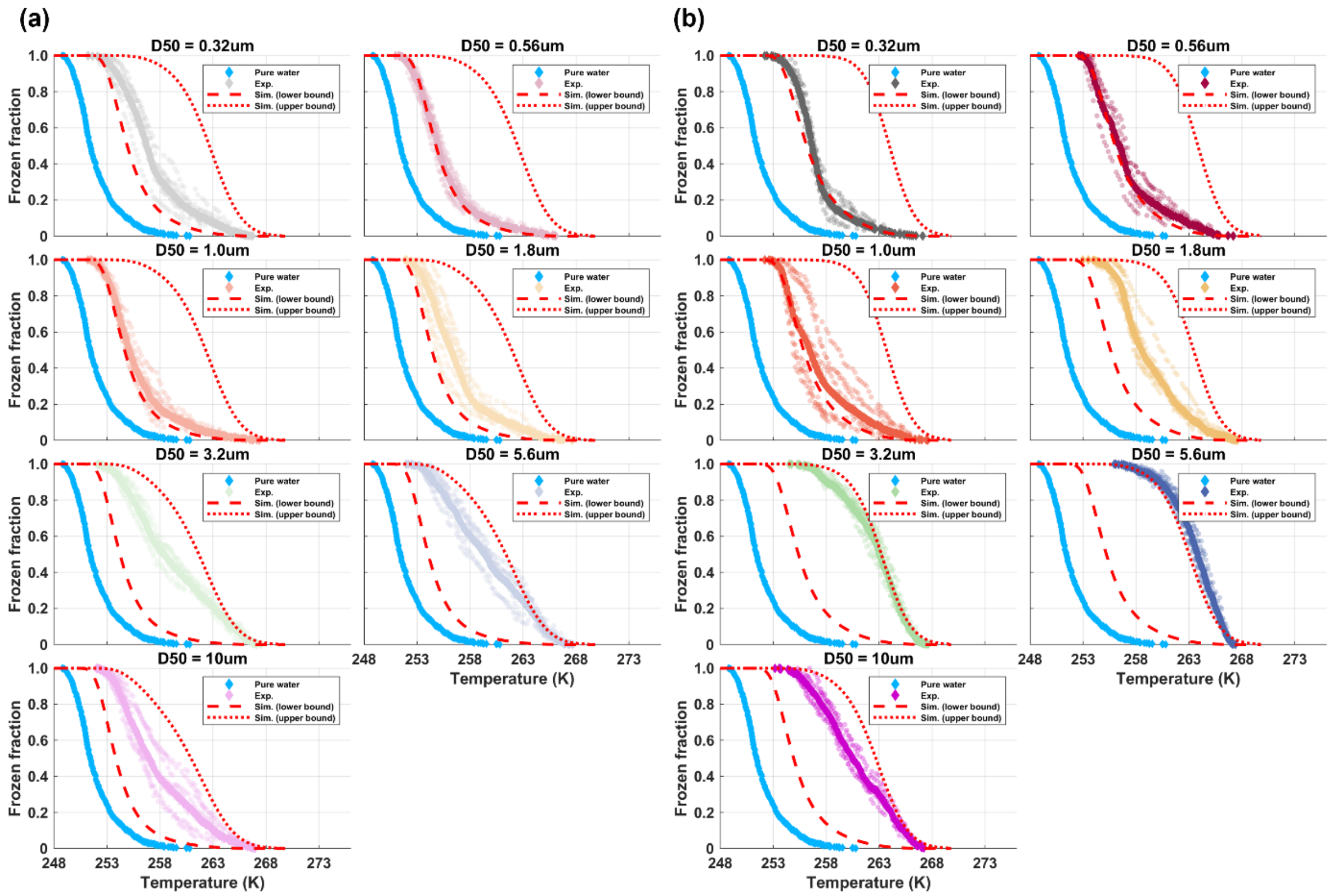

Figure S26. Comparison of measured and simulated frozen fractions for all impactor stages at (a) 0.05 wt% and (b) 0.1 wt%. Dotted lines show simulations using the upper bound of constant INAS densities (Fig. S25a), while dashed lines show simulations using the lower bound (Fig. S25b).

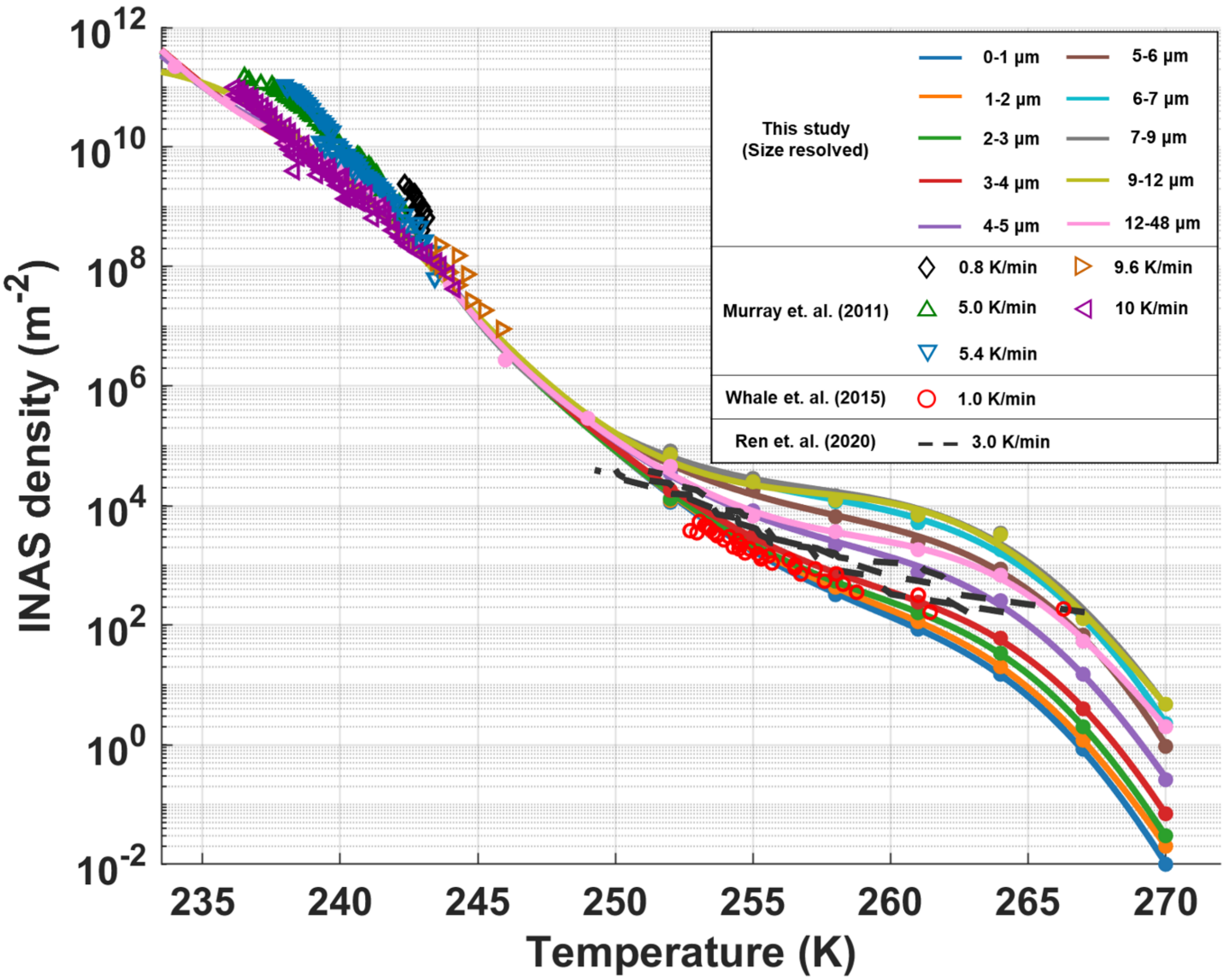

Figure S27. INAS density fits derived in this study shown together with literature results from droplet freezing experiments on KGa-1b performed with the bulk samples. Data are from experiments by Murray et al. (2011) performed with cooling rates from 0.8 to 10 K min^{-1}, Whale et al. (2015) with a cooling rate of 1 K min^{-1} and Ren et al. (2020) with a cooling rate of 3 K min^{-1}. In this study, the cooling rate was 1 K min^{-1}.

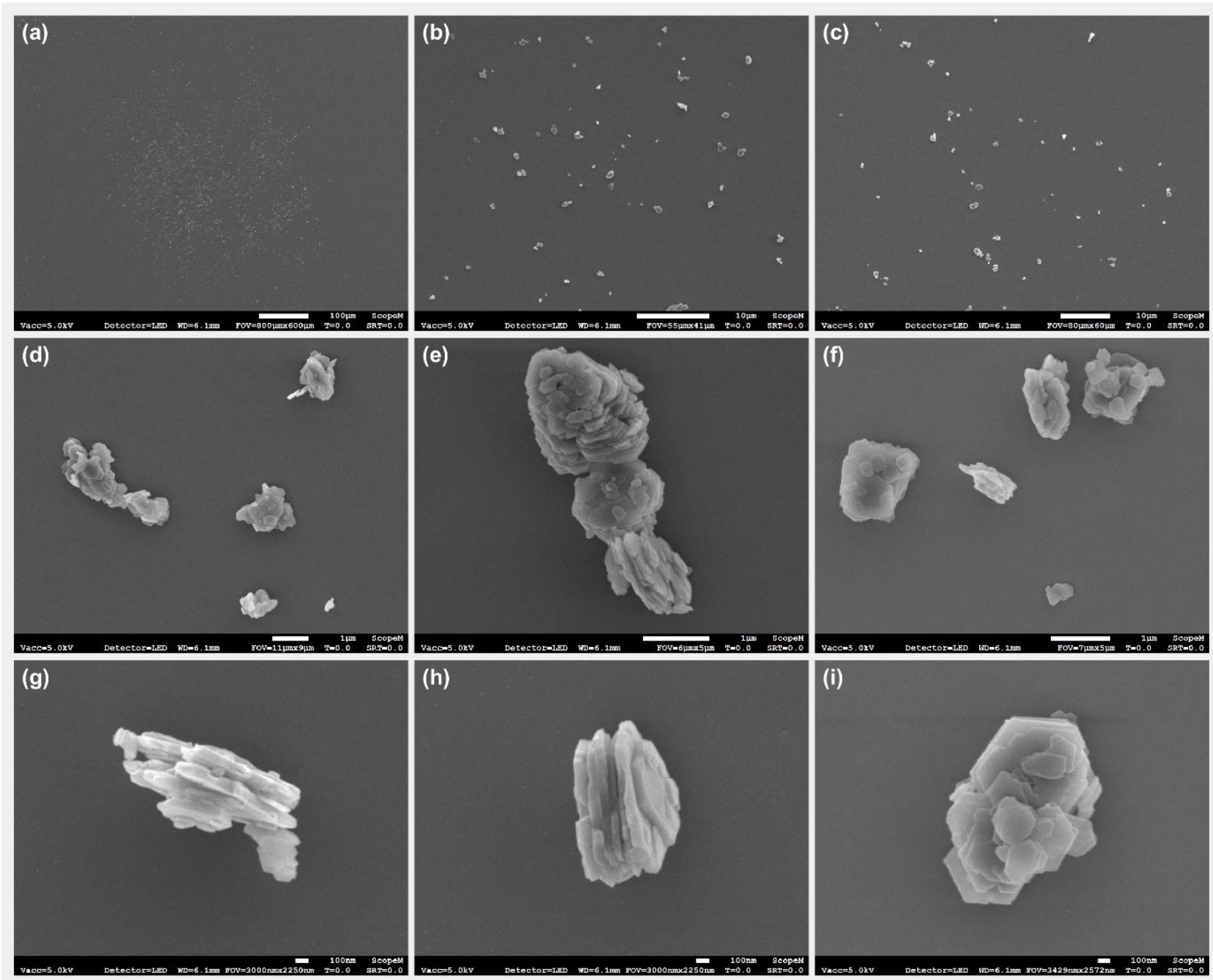

Figure S28. SEM images of particles that impacted on the stage with **D50 = 0.32 µm** from low to high magnification levels. (a) Low magnification (×150) showing the overall particle distribution; (b–c) Medium magnification (×2200 and ×1500), highlighting individual particle clusters; (d–f) High magnification (×11,000, ×20,000, and ×18,000), focusing on particle morphology; (g–i) Ultra-high magnification (×40,000, ×40,000, and ×35,000), revealing fine surface details. Panel h is also included in the composite image shown in Fig. 4d.

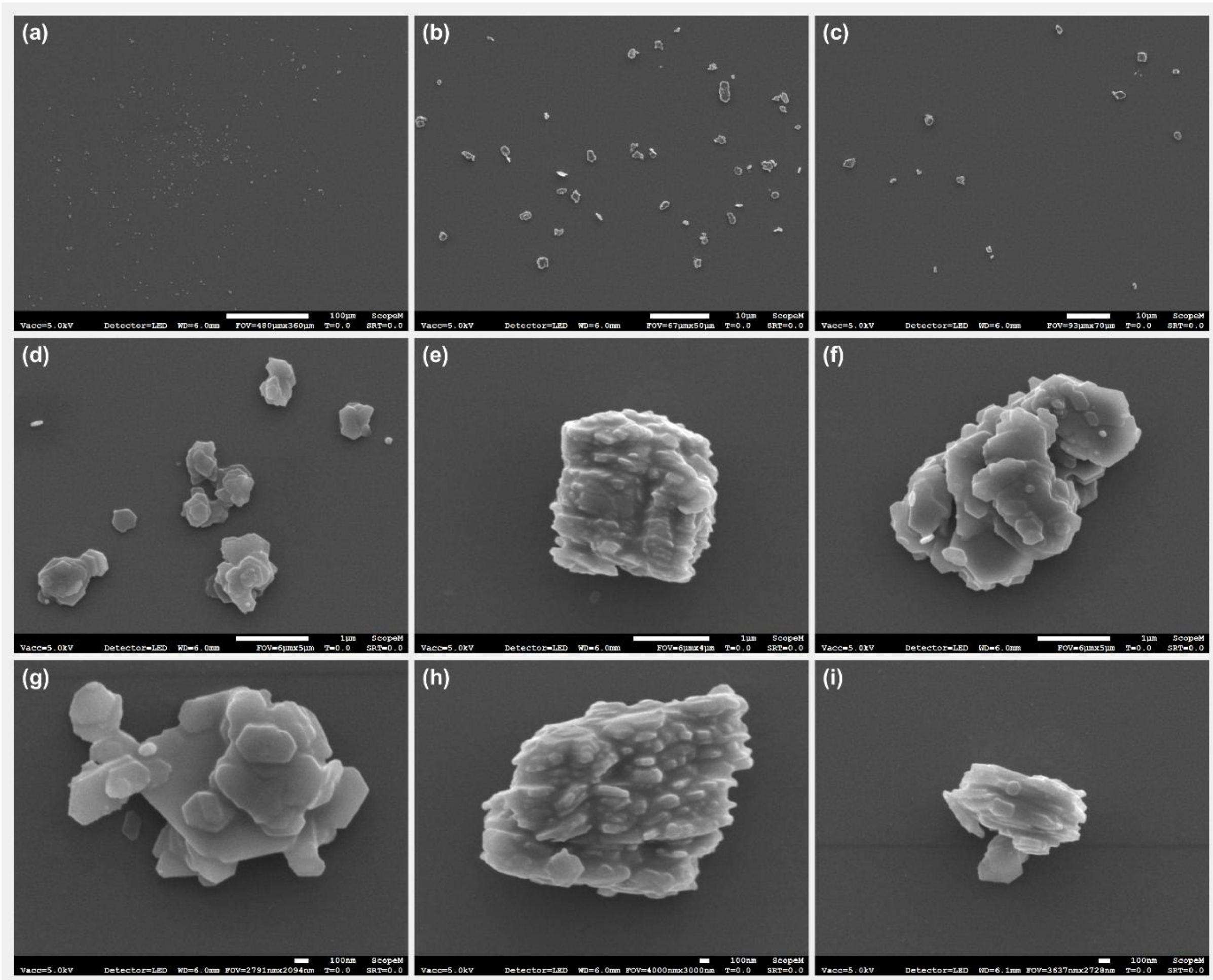

Figure S29. SEM images of particles that impacted on the stage with **D50 = 0.56 µm** from low to high magnification levels. (a) Low magnification (×250) showing the overall particle distribution; (b–c) Medium magnification (×1800 and ×1300), highlighting individual particle clusters; (d–f) High magnification (×22,000, ×23,000, and ×22,000), focusing on particle morphology; (g–i) Ultra-high magnification (×43,000, × 30,000, and × 33,000), revealing fine surface details.

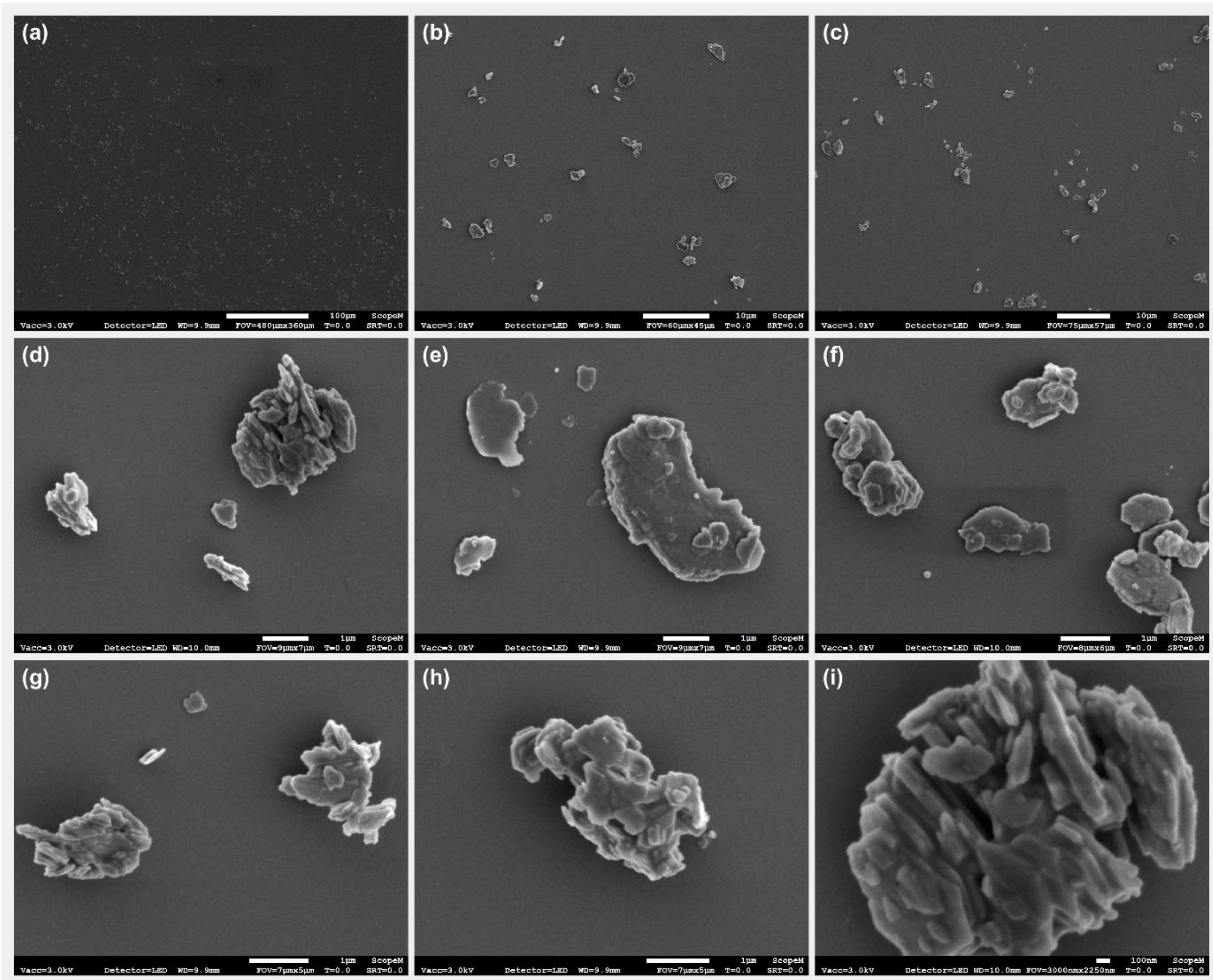

Figure S30. SEM images of particles that impacted on the stage with **D50 = 1.0 µm** from low to high magnification levels. (a) Low magnification (×250) showing the overall particle distribution; (b–c) Medium magnification (×2000 and ×1600), highlighting individual particle clusters; (d–h) High magnification (×14,000, ×14,000, ×15,000, ×18,000, ×19,000), focusing on particle morphology; (i) Ultra-high magnification (×40,000), revealing fine surface details.

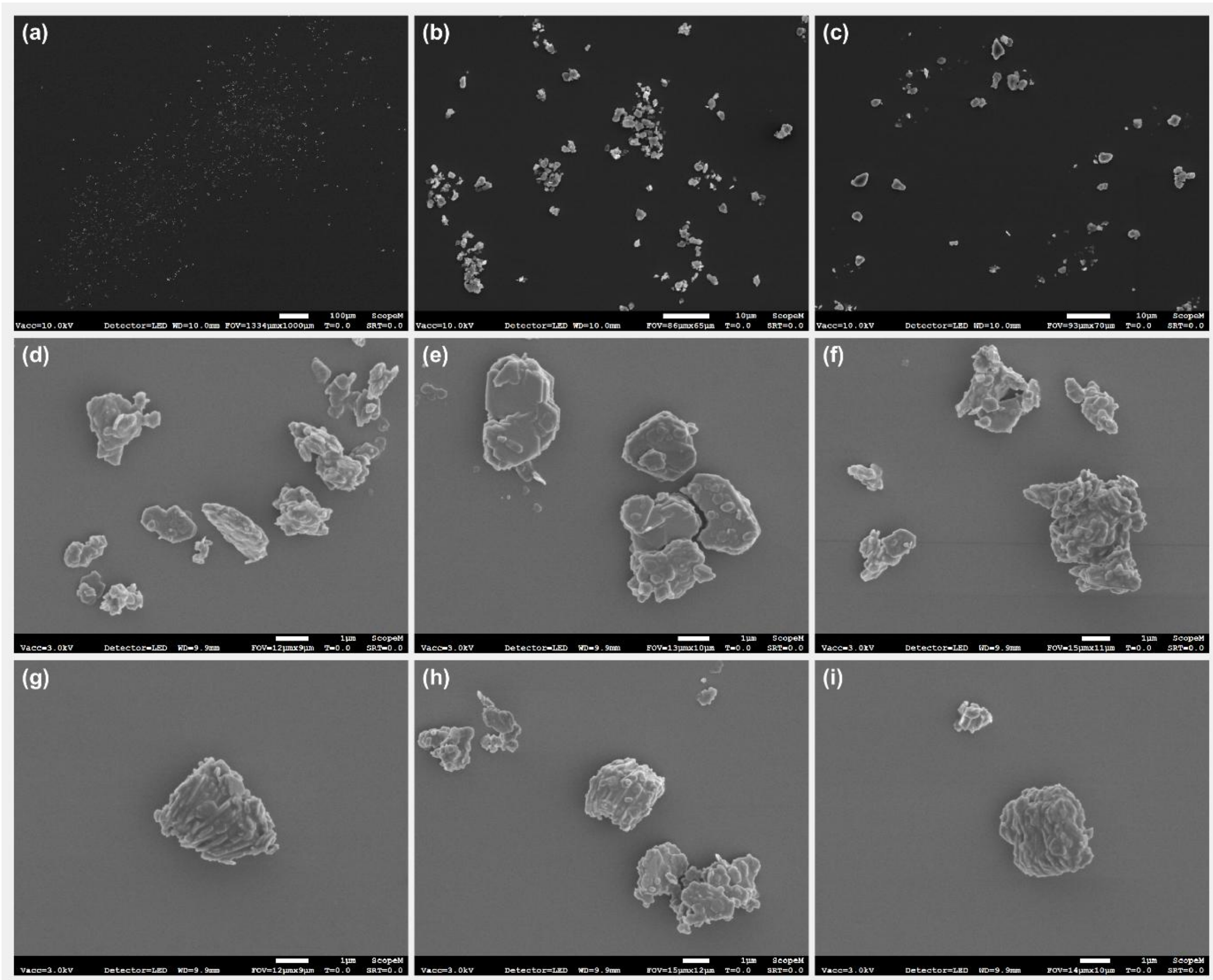

Figure S31. SEM images of particles collected that impacted on the stage with **D50 = 1.8 µm** from low to high magnification levels. (a) Low magnification (×90) showing the overall particle distribution; (b–c) Medium magnification (×1400 and ×1300), highlighting individual particle clusters; (d–i) High magnification (×10,000, ×9,500, ×8,500, ×10,000, ×8,000, ×9,000), focusing on particle morphology.

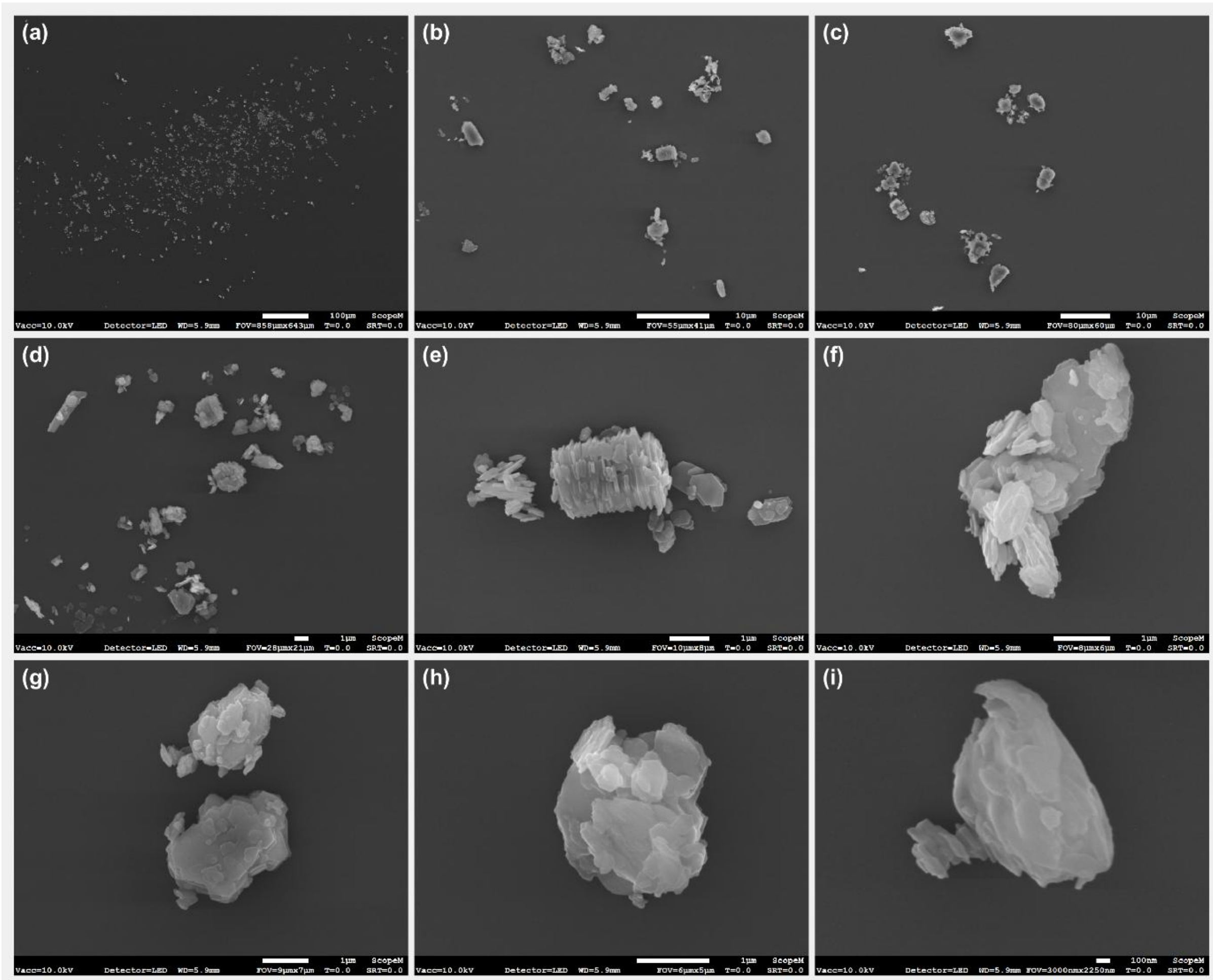

Figure S32. SEM images of particles that impacted on the stage with **D50 = 3.2 µm** from low to high magnification levels. (a) Low magnification (×140) showing the overall particle distribution; (b–d) Medium magnification (×2200, ×1500, and ×4300), highlighting individual particle clusters; (e–i) High magnification (×12,000, ×17,000, ×14,000, ×22,000, and ×40,000), focusing on particle morphology.

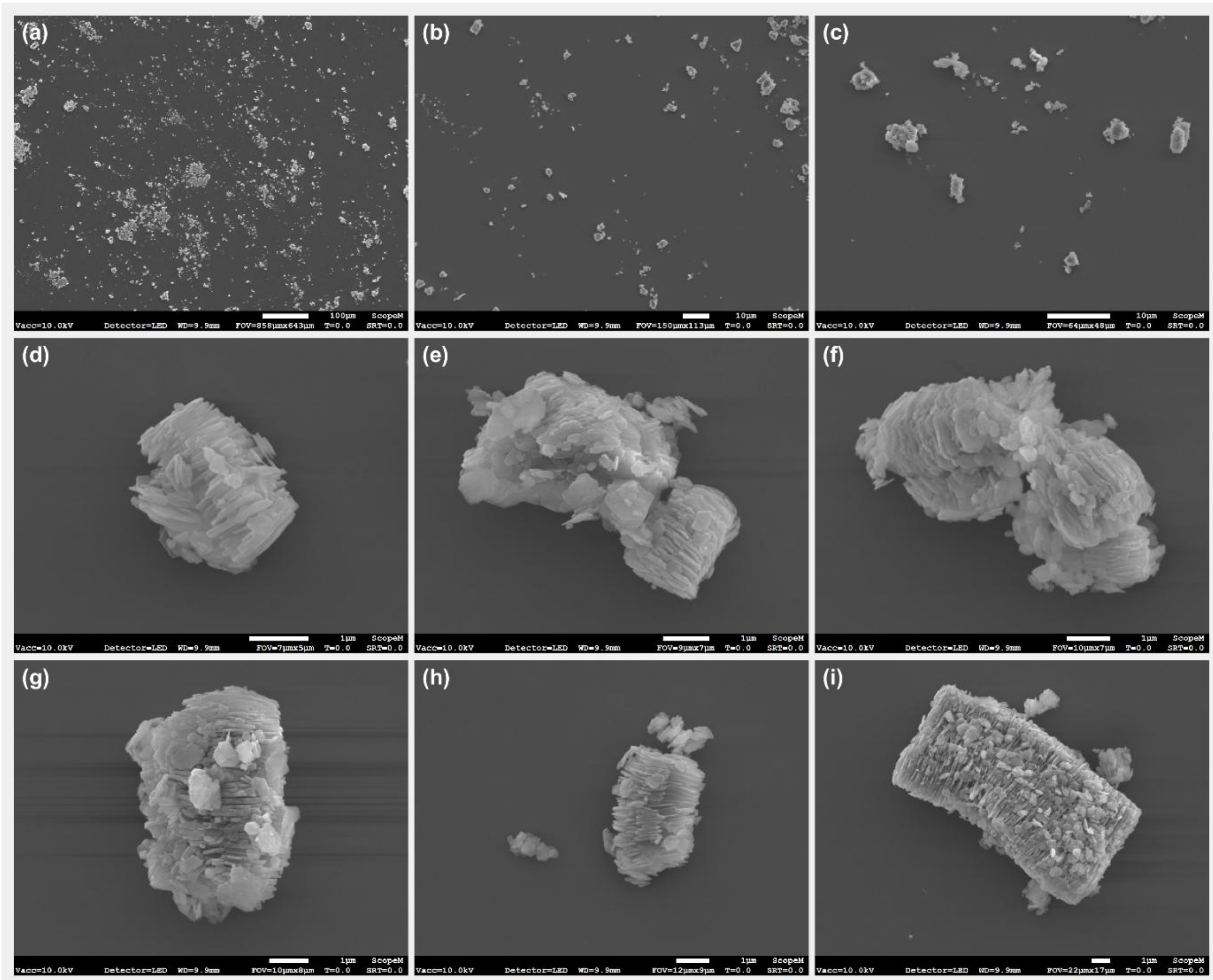

Figure S33. SEM images of particles that impacted on the stage with **D50 = 5.6 µm** from low to high magnification levels. (a) Low magnification (×140) showing the overall particle distribution; (b–c) Medium magnification (×800 and ×1900), highlighting individual particle clusters; (d–i) High magnification (×18,000, ×14,000, ×13,000, ×12,000, ×10,000, and ×5500), focusing on particle morphology. Panel d is also included in the composite image shown in Fig. 4e.

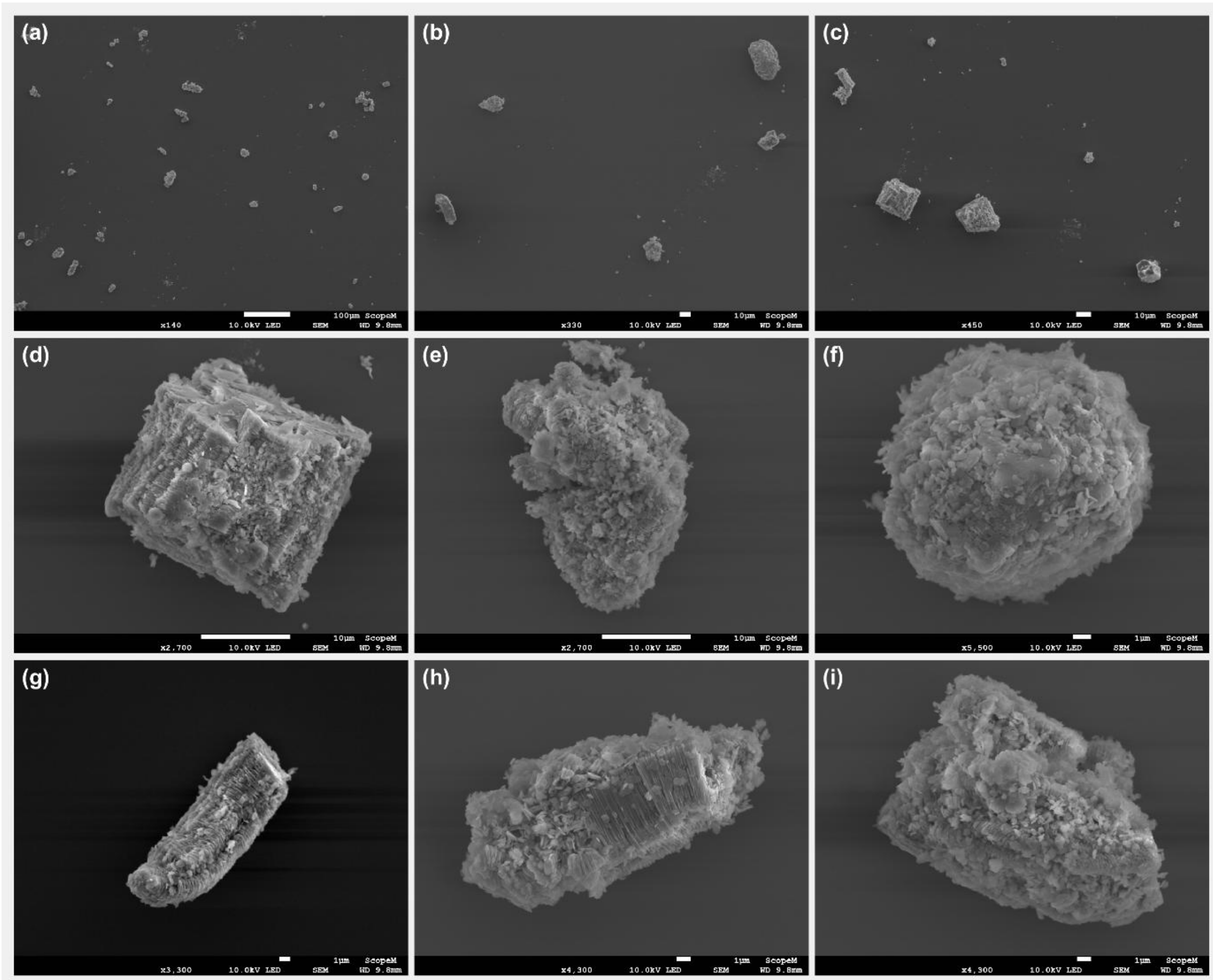

Figure S34. SEM images of particles that impacted on the stage with **D50 = 10 µm** from low to high magnification levels. (a) Low magnification (×140) showing the overall particle distribution; (b–c) Medium magnification (×330 and ×450), highlighting individual particle clusters; (d–i) High magnification (×2700, ×2700, ×5500, ×3300, ×4300, and ×4300), focusing on particle morphology. Panels h and i are also included in the composite image shown in Fig. 4f.

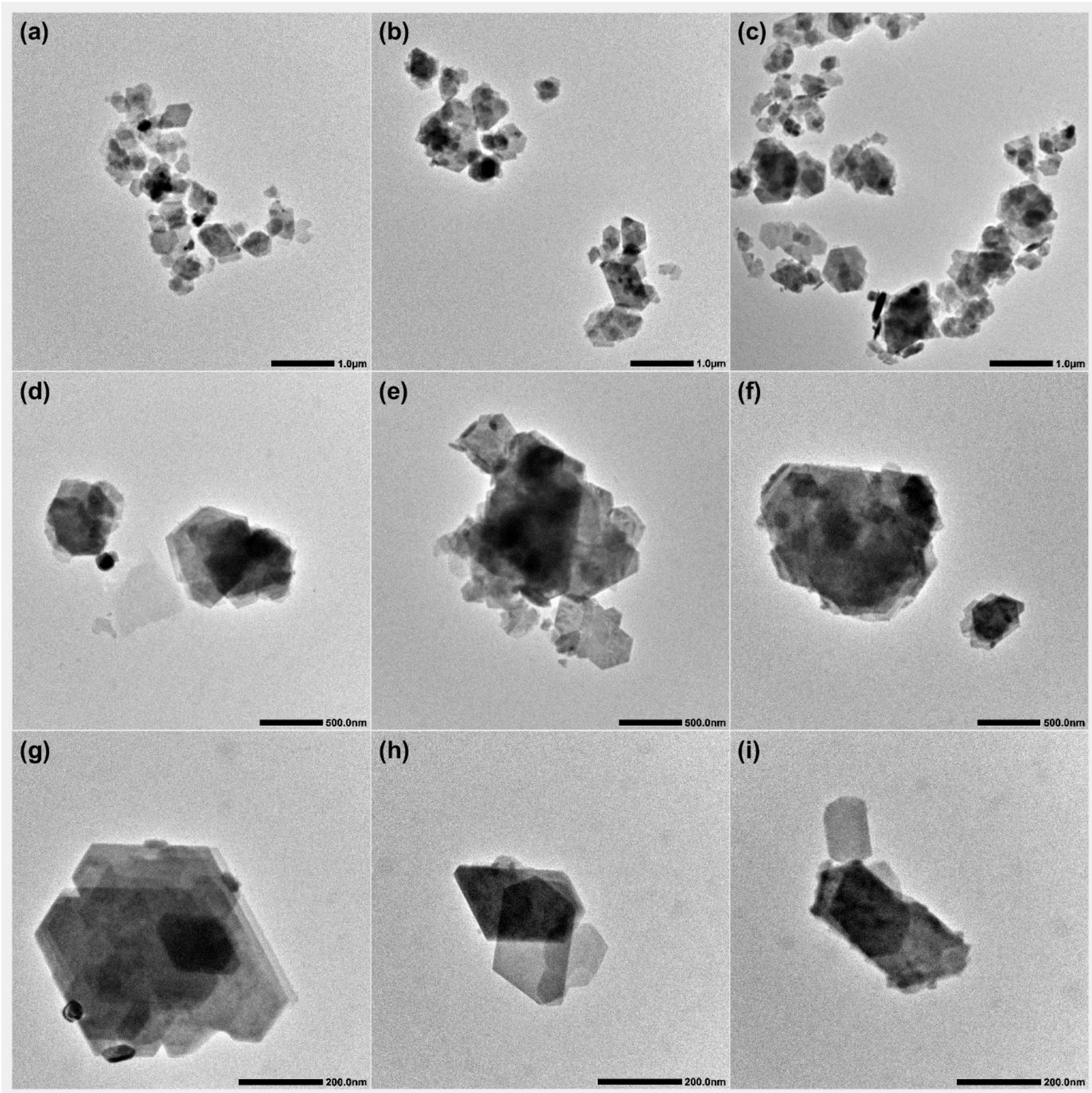

Figure S35. TEM images of particles that impacted on the stage with **D50 = 0.32 μm** at increasing magnification levels. (a–c) Low magnification (×6000), showing overall particle distribution; (d–f) Medium magnification (×12,000), highlighting individual particle clusters; (g–i) High magnification (×40,000), focusing on detailed particle morphology. Panels g and h are also included in the composite image shown in Fig. 4a.

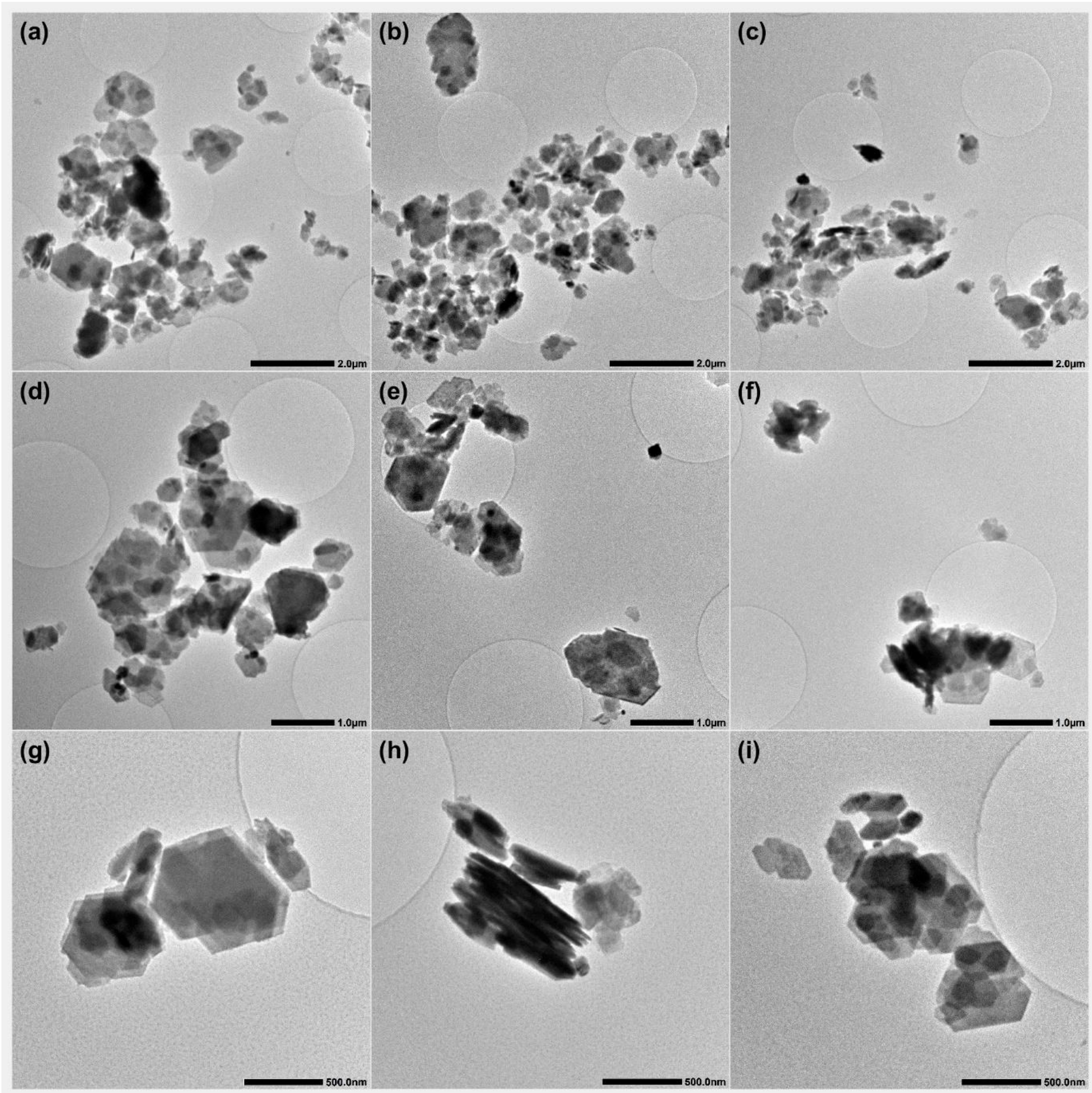

Figure S36. TEM images of particles that impacted on the stage with **D50 = 0.56 µm** at increasing magnification levels. (a–c) Low magnification (×4000), showing overall particle distribution; (d–f) Medium magnification (×6000), highlighting individual particle clusters; (g–i) High magnification (×15,000), focusing on detailed particle morphology.

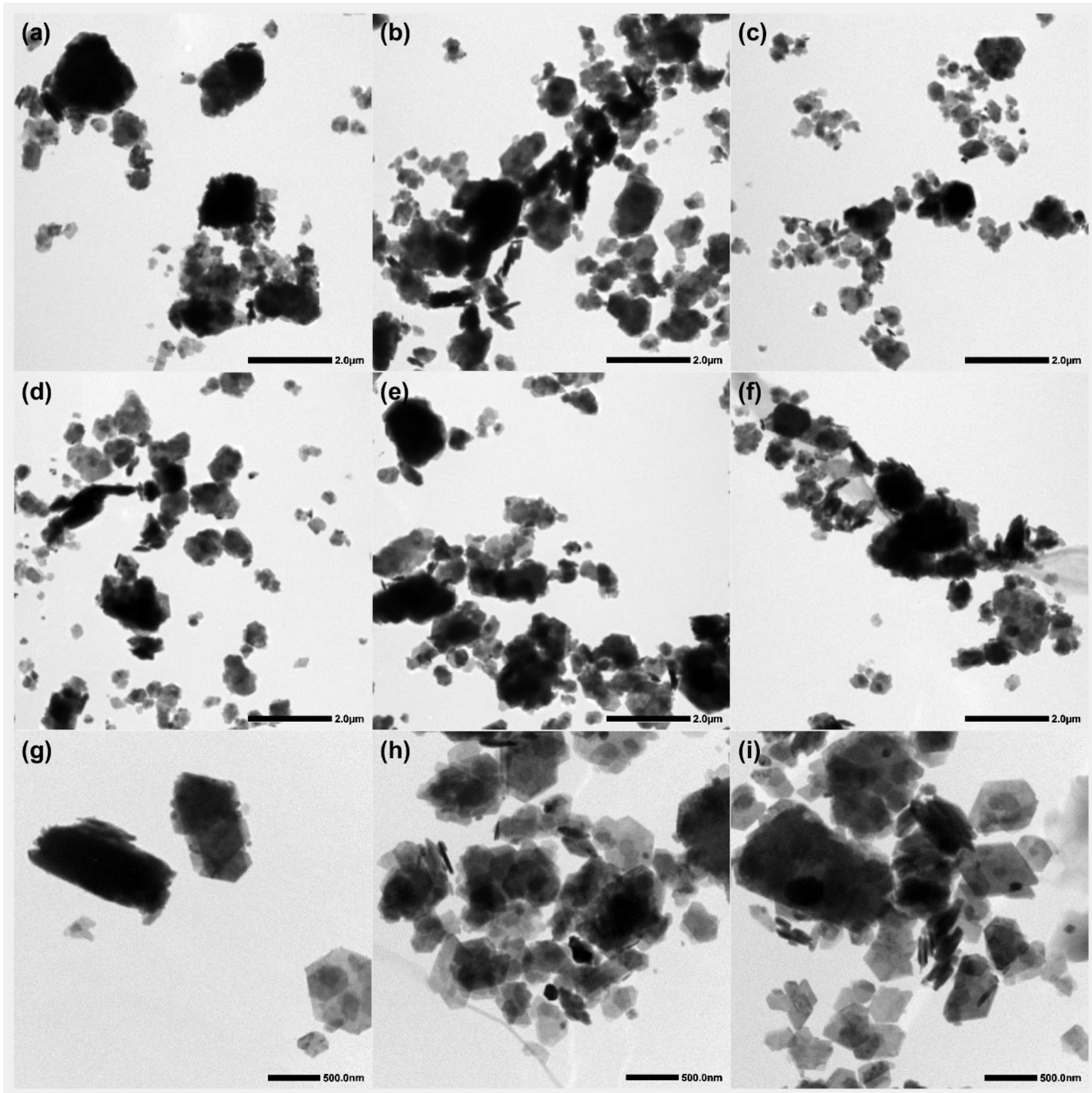

Figure S37. TEM images of particles that impacted on the stage with **D50 = 1.0 μm** at increasing magnification levels. (a–f) Low magnification (×4000), showing overall particle distribution; (g–i) Medium magnification (×10,000), highlighting individual particle clusters.

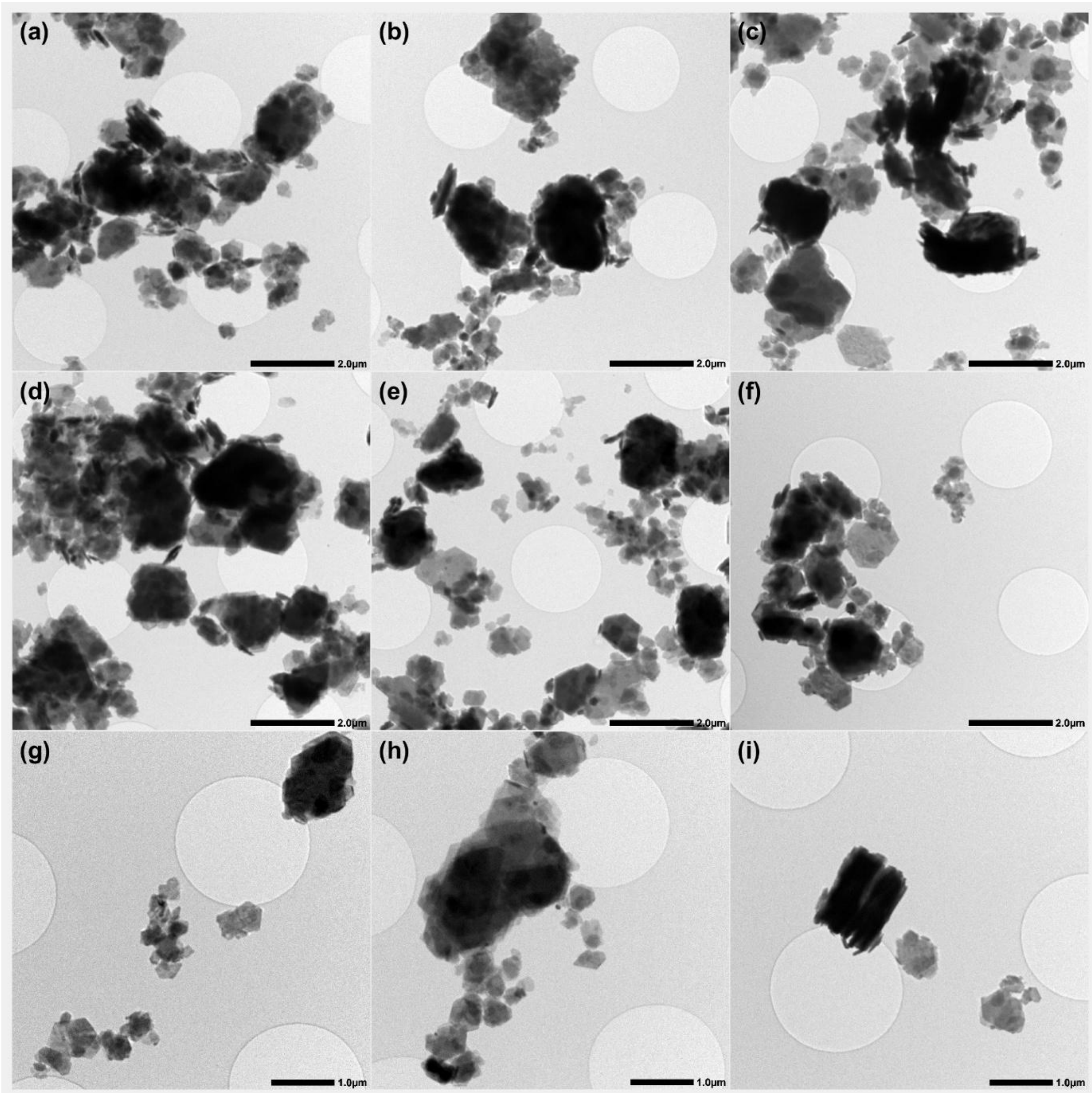

Figure S38. TEM images of particles that impacted on the stage with **D50 = 1.8 μm** at increasing magnification levels. (a–f) Low magnification (×4000), showing overall particle distribution; (g–i) Medium magnification (×6000), highlighting individual particle clusters. Panel i is also included in the composite image shown in Fig. 4c.

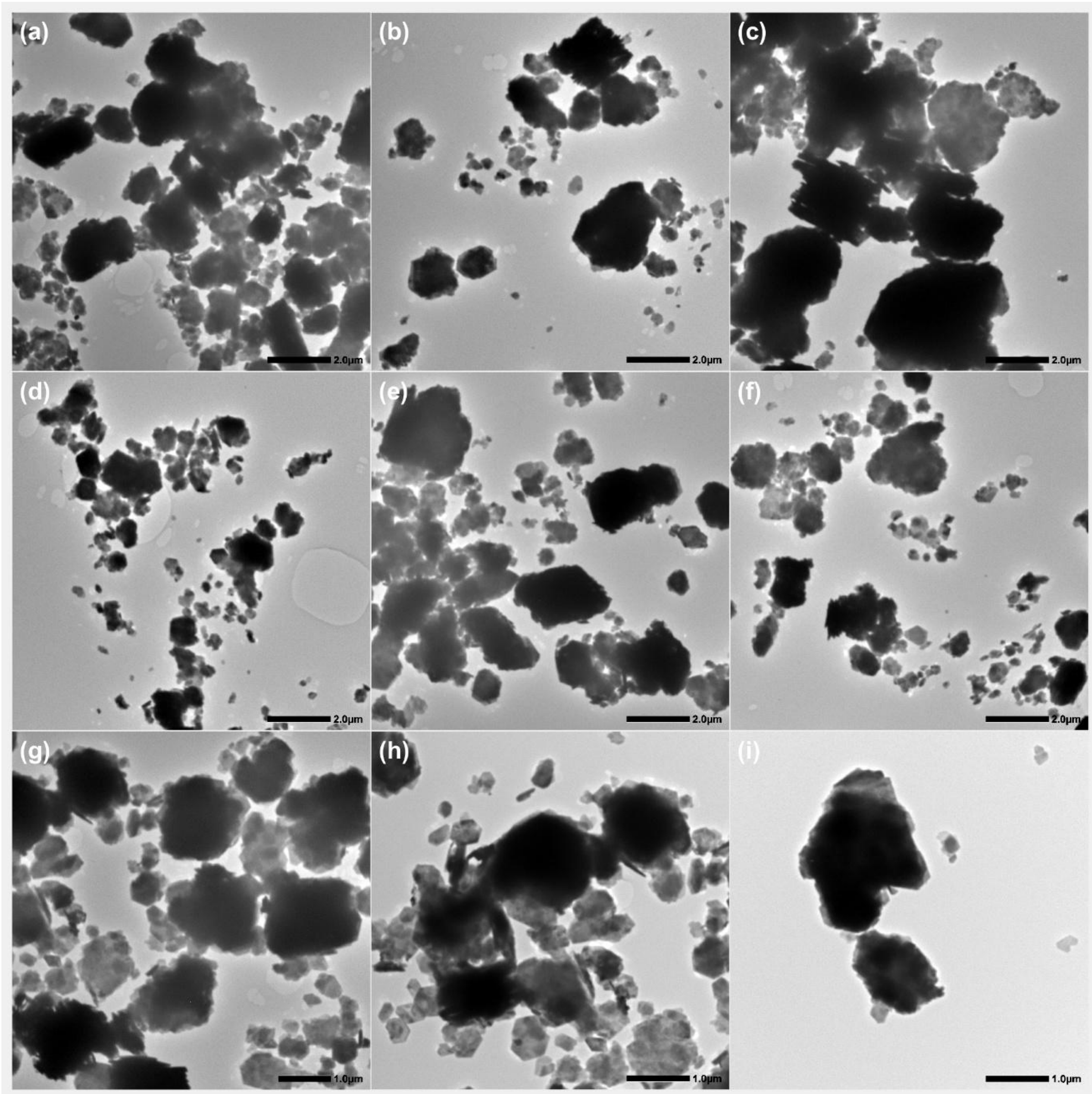

Figure S39. TEM images of particles that impacted on the stage with **D50 = 3.2 μm** at increasing magnification levels. (a–f) Low magnification (×3000), showing overall particle distribution; (g–i) Medium magnification (×5000, ×6000, ×6000), highlighting individual particle clusters.

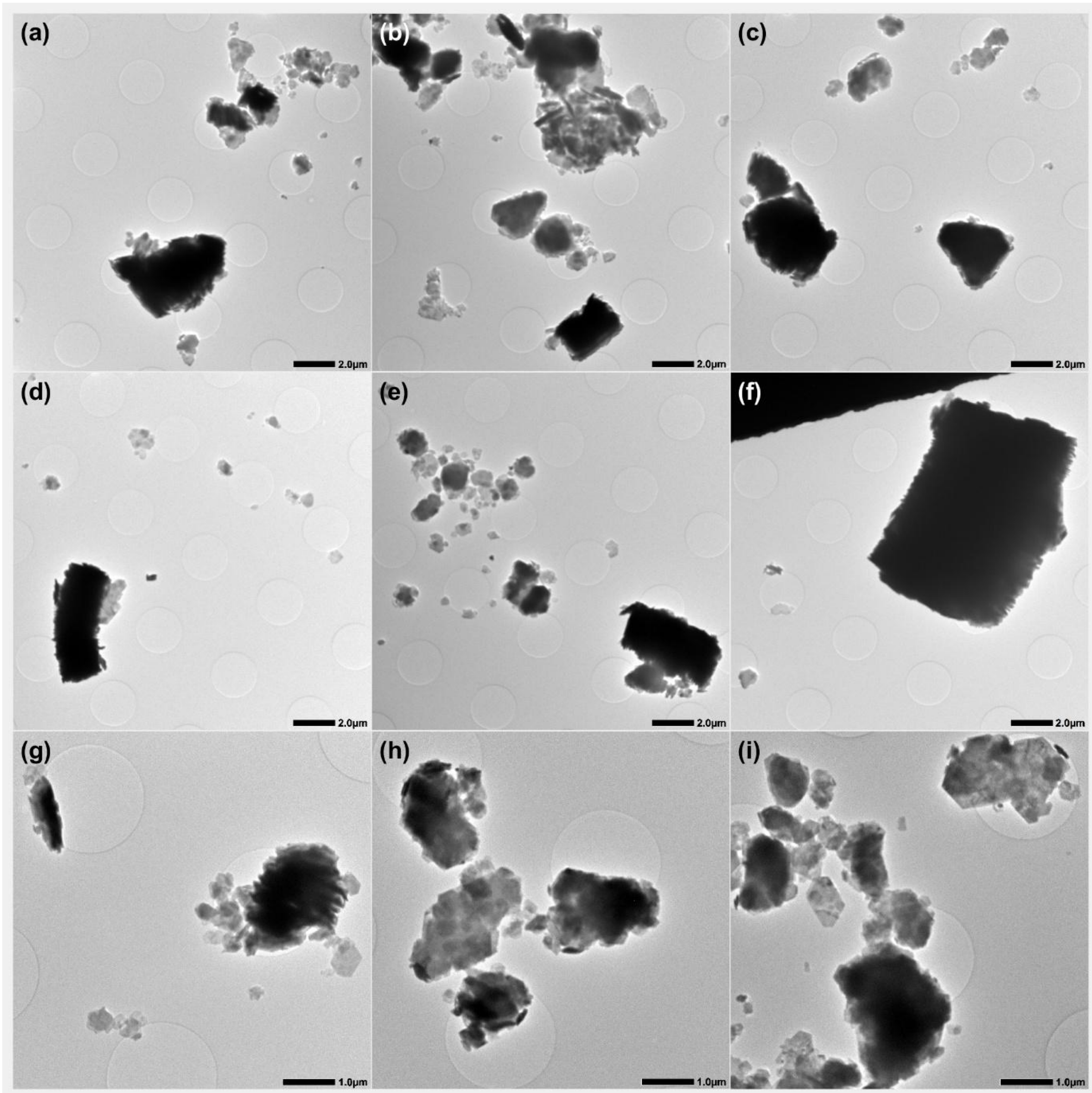

Figure S40. TEM images of particles that impacted on the stage with **D50 = 5.6 μm** at increasing magnification levels. (a–f) Low magnification (×2000), showing overall particle distribution; (g–i) Medium magnification (×5000), highlighting individual particle clusters.

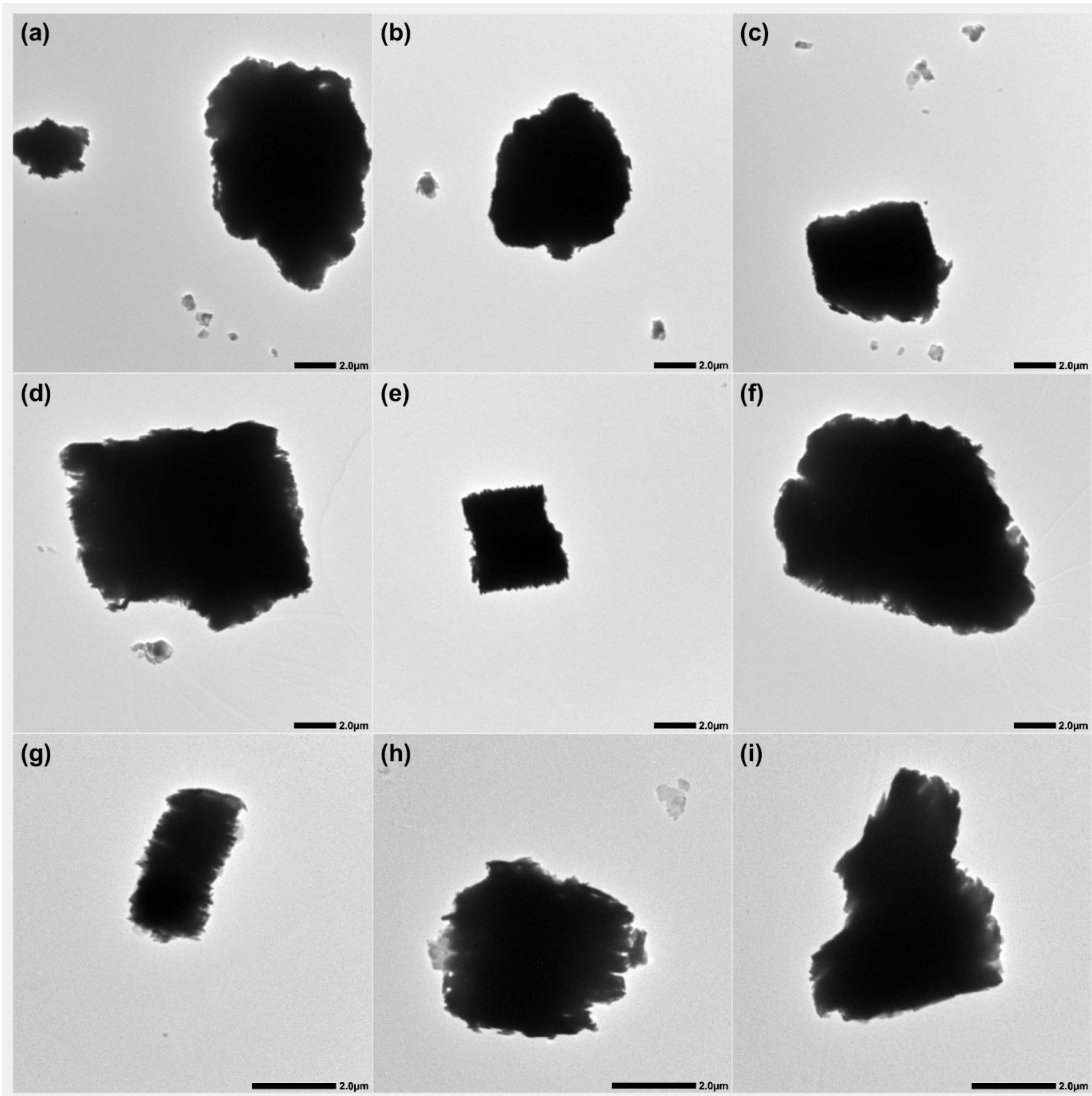

Figure S41. TEM images of particles that impacted on the stage with **D50 = 10 µm** at increasing magnification levels. (a–f) Low magnification (×2000); (g–i) Medium magnification (×4000). Both sets of images highlight the morphology of large particles.

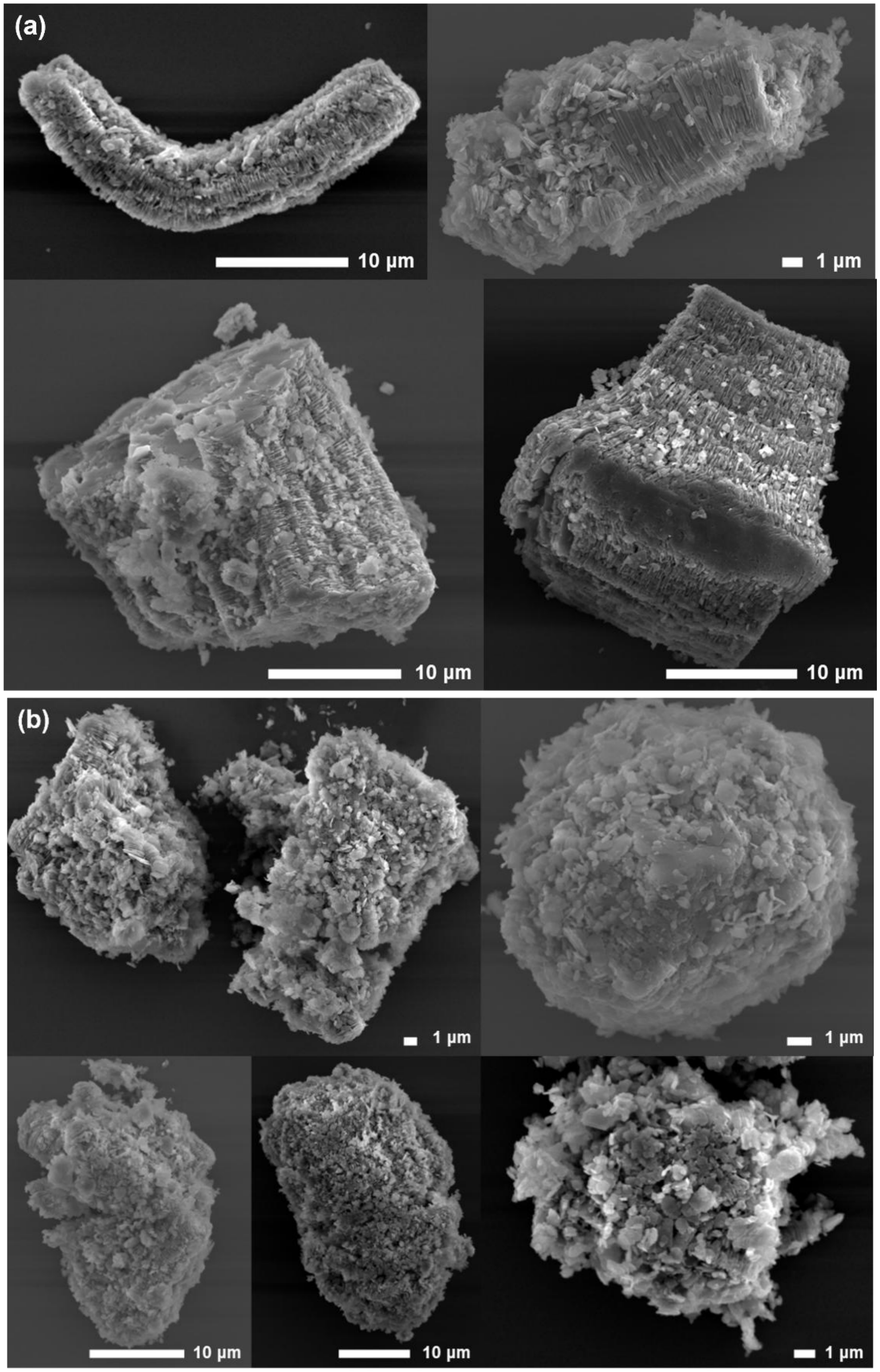

Figure S42. SEM images of particles in the largest size class (12–48 μm): (a) particles exhibiting a clear edge–basal distinction; (b) particles lacking a clear edge–basal distinction. Panel a is a composite image incorporating panel h from Fig. S34, which is also included in Fig. 4f. Panel b is a composite image incorporating panels e and f from Fig. S34.

Table S1. Fitting parameters for the three-parameter Boltzmann fit of the SA water freezing background and for the 95th percentile confidence interval for DRINCZ and MINCZ experiments.

		a	b	c	R^2
DRINCZ	Best	2.345	250.06	0.2091	0.9994
	-95th%	2.272	250.04	0.2011	NA
	+95th%	2.419	250.08	0.2171	NA
MINCZ	Best	4.9225	237.0390	0.8279	0.9994
	-95th%	4.8580	237.0297	0.8026	NA
	+95th%	4.9870	237.0483	0.8533	NA

Table S2. Mass and relative abundance (%) of particles collected on each MOUDI stage (D50 = 0.056–18 μm) after collecting for 6 days. Bold rows indicate the impactor stages with sufficient particle mass for subsequent experiments and analysis. For the D50 = 0.32 μm stage, the notation "+22" indicates that an additional 22 mg of sample was obtained from a second MOUDI run for 2.5 days, since the initial 13.7 mg was insufficient for the full set of experiments.

**D50 (μm)**	**Stage**	**Mass (mg)**	**Mass ratio (%)**
18	inlet	8.4	1.44
**10**	**S01**	**43.0**	**7.37**
**5.6**	**S02**	**57.7**	**9.89**
**3.2**	**S03**	**141.1**	**24.18**
**1.8**	**S04**	**140.3**	**24.04**
**1.0**	**S05**	**135.0**	**23.13**
**0.56**	**S06**	**40.0**	**6.85**
**0.32**	**S07**	**13.7 (+22)**	**2.35**
0.18	S08	1.3	0.22
0.10	S09	1.5	0.26
0.056	S10	1.6	0.27

Table S3. Average number of droplets per MINCZ experiment (N_{av}) for each investigated impactor stage.

**Impactor stage (D50, µm)**	**0.05 wt%**	**0.1 wt%**	**0.5 wt%**	**1.0 wt%**
0.32	81	87	71	\
0.56	80	102	84	\
1.0	109	80	74	86
1.8	93	105	119	42
3.2	85	120	97	\
5.6	112	100	107	\
10	67	93	114	\

Table S4. Calculated average number of particles per size bin at 0.05wt% and 0.1wt% for **D50 = 0.32 µm** in individual **DRINCZ (50 µL)** droplets.

**Size bin**	**0.05wt%**	**0.1wt%**
0.0-0.2	2180484	4360967
0.2-0.4	1708046	3416091
0.4-0.6	2580239	5160478
0.6-0.8	4070236	8140472
0.8-1.0	3997553	7995107
1.0-1.2	2216825	4433650
1.2-1.4	2107801	4215602
1.4-1.6	1017559	2035118
1.6-1.8	981218	1962435
1.8-2.0	399755	799511
2.0-2.2	327073	654145
2.2-2.4	145366	290731
2.4-2.6	36341	72683
2.6-2.8	72683	145366
2.8-3.0	36341	72683

Table S5. Calculated average number of particles per size bin at 0.05wt% and 0.1wt% for **D50 = 0.56 µm** in individual **DRINCZ (50 µL)** droplets.

Size bin	0.05wt%	0.1wt%
0.0-0.2	1800460	3600921
0.2-0.4	2260578	4521156
0.4-0.6	2320593	4641187
0.6-0.8	1860476	3720951
0.8-1.0	1580404	3160808
1.0-1.2	980251	1960501
1.2-1.4	720184	1440368
1.4-1.6	740189	1480378
1.6-1.8	460118	920235
1.8-2.0	480123	960246
2.0-2.2	420107	840215
2.2-2.4	260066	520133
2.4-2.6	120031	240061
2.6-2.8	40010	80020
2.8-3.0	80020	160041
3.0-3.2	60015	120031
3.2-3.4	20005	40010
3.4-3.6	60015	120031
3.6-3.8	40010	80020

Table S6. Calculated average number of particles per size bin at 0.05wt% and 0.1wt% for **D50 = 1.0 µm** in individual **DRINCZ (50 µL)** droplets.

**Size bin**	**0.05wt%**	**0.1wt%**
0.0-0.2	895809	1791618
0.2-0.4	1034814	2069628
0.4-0.6	1112039	2224078
0.6-0.8	787694	1575388
0.8-1.0	864919	1729838
1.0-1.2	695024	1390049
1.2-1.4	478795	957589
1.4-1.6	386125	772249
1.6-1.8	432460	864919
1.8-2.0	447905	895809
2.0-2.2	370680	741359
2.2-2.4	247120	494240
2.4-2.6	169895	339790
2.6-2.8	77225	154450
2.8-3.0	123560	247120
3.0-3.2	61780	123560
3.2-3.4	46335	92670
3.4-3.6	30890	61780
3.6-3.8	30890	61780
4.2-4.4	15445	30890
4.4-4.6	15445	30890

Table S7. Calculated average number of particles per size bin at 0.05wt% and 0.1wt% for **D50 = 1.8 µm** in individual **DRINCZ (50 µL)** droplets.

Size bin	0.05wt%	0.1wt%
0.0-0.2	259444	518888
0.2-0.4	328212	656425
0.4-0.6	318835	637670
0.6-0.8	268821	537643
0.8-1.0	218808	437616
1.0-1.2	140662	281325
1.2-1.4	187550	375100
1.4-1.6	118782	237563
1.6-1.8	146914	293828
1.8-2.0	109404	218808
2.0-2.2	106278	212557
2.2-2.4	118782	237563
2.4-2.6	106278	212557
2.6-2.8	109404	218808
2.8-3.0	65642	131285
3.0-3.2	62517	125033
3.2-3.4	46887	93775
3.4-3.6	59391	118782
3.6-3.8	53139	106278
3.8-4.0	34384	68768
4.0-4.2	21881	43762
4.2-4.4	15629	31258
4.4-4.6	25007	50013
4.6-4.8	12503	25007
4.8-5.0	9377	18755
5.0-5.2	3126	6252
5.2-5.4	9377	18755
5.4-5.6	3126	6252
5.6-5.8	6252	12503
6.0-6.2	3126	6252

Table S8. Calculated average number of particles per size bin at 0.05wt% and 0.1wt% for **D50 = 3.2 µm** in individual **DRINCZ (50 µL)** droplets.

Size bin	0.05wt%	0.1wt%	Size bin	0.05wt%	0.1wt%
0.0-0.2	67374	134748	4.2-4.4	24394	48788
0.2-0.4	97576	195152	4.4-4.6	17424	34849
0.4-0.6	65051	130101	4.6-4.8	10455	20909
0.6-0.8	94091	188182	4.8-5.0	8131	16263
0.8-1.0	102222	204445	5.0-5.2	8131	16263
1.0-1.2	84798	169596	5.2-5.4	4646	9293
1.2-1.4	95253	190505	5.4-5.6	9293	18586
1.4-1.6	88283	176566	5.6-5.8	3485	6970
1.6-1.8	99899	199798	5.8-6.0	10455	20909
1.8-2.0	69697	139394	6.0-6.2	1162	2323
2.0-2.2	82475	164950	6.2-6.4	2323	4646
2.2-2.4	76667	153334	6.4-6.6	2323	4646
2.4-2.6	55758	111515	6.6-6.8	4646	9293
2.6-2.8	34849	69697	6.8-7.0	1162	2323
2.8-3.0	36010	72020	7.2-7.4	1162	2323
3.0-3.2	37172	74344	7.6-7.8	3485	6970
3.2-3.4	33687	67374	8.2-8.4	1162	2323
3.4-3.6	22071	44141	8.6-8.8	1162	2323
3.6-3.8	22071	44141	8.8-9.0	1162	2323
3.8-4.0	9293	18586	9.8-10.0	1162	2323
4.0-4.2	12778	25556	10.0-10.2	1162	2323

Table S9. Calculated average number of particles per size bin at 0.05wt% and 0.1wt% for **D50 = 5.6 µm** in individual **DRINCZ (50 µL)** droplets.

Size bin	0.05wt%	0.1wt%	Size bin	0.05wt%	0.1wt%
0.0-0.2	22570	45139	4.8-5.0	10259	20518
0.2-0.4	45139	90278	5.0-5.2	5129	10259
0.4-0.6	54372	108744	5.2-5.4	7181	14362
0.6-0.8	77968	155935	5.4-5.6	6155	12311
0.8-1.0	91304	182608	5.6-5.8	1026	2052
1.0-1.2	76942	153883	5.8-6.0	5129	10259
1.2-1.4	59502	119003	6.0-6.2	7181	14362
1.4-1.6	41036	82071	6.4-6.6	7181	14362
1.6-1.8	41036	82071	6.6-6.8	2052	4104
1.8-2.0	34880	69760	6.8-7.0	3078	6155
2.0-2.2	35906	71812	7.0-7.2	3078	6155
2.2-2.4	24621	49243	7.2-7.4	1026	2052
2.4-2.6	22570	45139	7.4-7.6	1026	2052
2.6-2.8	15388	30777	7.6-7.8	3078	6155
2.8-3.0	10259	20518	8.0-8.2	1026	2052
3.0-3.2	19492	38984	8.2-8.4	1026	2052
3.2-3.4	9233	18466	8.4-8.6	1026	2052
3.4-3.6	11285	22570	8.6-8.8	4104	8207
3.6-3.8	15388	30777	8.8-9.0	1026	2052
3.8-4.0	15388	30777	9.0-9.2	1026	2052
4.0-4.2	9233	18466	9.4-9.6	1026	2052
4.2-4.4	9233	18466	10.0-10.2	1026	2052
4.4-4.6	10259	20518	10.8-11.0	1026	2052
4.6-4.8	6155	12311	11.4-11.6	1026	2052

Table S10. Calculated average number of particles per size bin at 0.05wt% and 0.1wt% for **D50 = 10 µm** in individual **DRINCZ (50 µL)** droplets.

Size bin	0.05wt%	0.1wt%	Size bin	0.05wt%	0.1wt%	Size bin	0.05wt%	0.1wt%
0.0-0.2	6993	13986	7.2-7.4	77	155	18.0-18.2	26	52
0.2-0.4	7845	15689	7.4-7.6	103	206	18.2-18.4	26	52
0.4-0.6	23224	46449	7.6-7.8	77	155	18.4-18.6	52	103
0.6-0.8	8206	16412	7.8-8.0	52	103	18.6-18.8	52	103
0.8-1.0	11225	22450	8.0-8.2	26	52	18.8-19.0	26	52
1.0-1.2	6580	13160	8.2-8.4	77	155	19.0-19.2	26	52
1.2-1.4	6271	12541	8.4-8.6	26	52	19.4-19.6	77	155
1.4-1.6	4464	8928	8.6-8.8	26	52	19.6-19.8	26	52
1.6-1.8	3432	6864	8.8-9.0	26	52	20.4-20.6	52	103
1.8-2.0	2709	5419	9.0-9.2	52	103	21.4-21.6	26	52
2.0-2.2	1858	3716	9.2-9.4	52	103	21.6-21.8	26	52
2.2-2.4	2142	4284	9.4-9.6	77	155	22.0-22.2	26	52
2.4-2.6	1290	2580	10.0-10.2	26	52	23.0-23.2	52	103
2.6-2.8	1264	2529	10.2-10.4	26	52	23.2-23.4	52	103
2.8-3.0	619	1239	10.4-10.6	26	52	23.8-24.0	77	155
3.0-3.2	619	1239	10.8-11.0	52	103	24.4-24.6	26	52
3.2-3.4	387	774	11.8-12.0	26	52	25.6-25.8	26	52
3.4-3.6	464	929	12.0-12.2	52	103	25.8-26.0	52	103
3.6-3.8	335	671	12.4-12.6	52	103	26.2-26.4	103	206
3.8-4.0	335	671	12.8-13.0	52	103	26.8-27.0	26	52
4.0-4.2	284	568	13.0-13.2	26	52	28.2-28.4	52	103
4.2-4.4	258	516	13.4-13.6	52	103	28.4-28.6	52	103
4.4-4.6	77	155	13.6-13.8	77	155	28.6-28.8	26	52
4.6-4.8	155	310	14.2-14.4	26	52	28.8-29.0	26	52
4.8-5.0	258	516	14.4-14.6	52	103	30.0-30.2	26	52
5.0-5.2	77	155	14.6-14.8	26	52	31.4-31.6	26	52
5.2-5.4	206	413	15.0-15.2	52	103	32.0-32.2	26	52
5.4-5.6	155	310	15.2-15.4	26	52	32.6-32.8	26	52
5.6-5.8	77	155	15.4-15.6	103	206	32.8-33.0	26	52
5.8-6.0	155	310	15.8-16.0	52	103	35.0-35.2	26	52
6.0-6.2	77	155	16.0-16.2	52	103	37.0-37.2	26	52
6.2-6.4	232	464	16.4-16.6	26	52	38.2-38.4	26	52
6.4-6.6	26	52	16.6-16.8	26	52	39.0-39.2	52	103
6.6-6.8	26	52	16.8-17.0	26	52	39.8-40.0	26	52
6.8-7.0	77	155	17.2-17.4	26	52	47.8-48.0	26	52
7.0-7.2	52	103	17.8-18.0	26	52			

Table S11. Aspect ratios of the KGa-1b particle size classes *i* determined from the SEM analysis.

**Size class *i***	**Size range (µm)**	**Aspect ratio**
1	0–1	1.405
2	1–2	1.509
3	2–3	1.572
4	3–4	1.605
5	4–5	1.616
6	5–6	1.649
7	6–7	1.604
8	7–9	1.615
9	9–12	1.521
10	12–48	1.589

Table S12. Specific surface areas (*SSA*) of the investigated impactor stages as determined by BET measurements (SSA_{BET}) and theoretical values based on the assumption of spherical particles (SSA_{th}).

Impactor stage (D50, µm)	SSA_{BET} (m^2g^{-1})	SSA_{th} (m^2g^{-1})
0.32	13.915	1.945
0.56	12.966	1.483
1.0	13.879	1.281
1.8	11.095	0.892
3.2	10.749	0.674
5.6	7.577	0.518
10	6.953	0.118

Table S13. Estimated actual surface areas per particle in each size class ($A_{i,\ class}$) with the corresponding 5th and 95th percentile uncertainty bounds derived from Monte Carlo analysis.

**Size class i**	**Size range (µm)**	**Median surface area per particle, A_{median} (m²)**	**Lower uncertainty bound, A_{lower} (5th percentile, m²)**	**Upper uncertainty bound, A_{upper} (95th percentile, m²)**
1	0–1	3.06×10^{-12}	2.98×10^{-12}	3.14×10^{-12}
2	1–2	3.58×10^{-11}	3.51×10^{-11}	3.66×10^{-11}
3	2–3	1.43×10^{-10}	1.39×10^{-10}	1.47×10^{-10}
4	3–4	3.36×10^{-10}	3.27×10^{-10}	3.46×10^{-10}
5	4–5	6.10×10^{-10}	5.85×10^{-10}	6.33×10^{-10}
6	5–6	1.00×10^{-9}	9.51×10^{-10}	1.04×10^{-9}
7	6–7	1.56×10^{-9}	1.49×10^{-9}	1.64×10^{-9}
8	7–9	2.76×10^{-9}	2.59×10^{-9}	2.94×10^{-9}
9	9–12	5.86×10^{-9}	5.32×10^{-9}	6.42×10^{-9}
10	12–48	7.52×10^{-8}	6.00×10^{-8}	9.36×10^{-8}

Table S14. Calculated threshold contact angles as a function of temperature.

**Temperature**	**Threshold contact angle (α_{thr}, unit: °)**
234	101.4
235	98.7
236	96.1
237	93.7
238	91.3
239	89.0
240	86.7
241	84.5
242	82.4
243	80.2
244	78.2
245	76.1
246	74.1
247	72.1
248	70.1
249	68.1
250	66.2
251	64.3
252	62.3
253	60.4
254	58.4
255	56.5
256	54.5
257	52.6
258	50.6
259	48.6
260	46.5
261	44.4
262	42.3
263	40.1
264	37.9
265	35.5
266	33.1
267	30.5
268	27.8
269	24.8
270	21.5
271	17.7
272	12.9
273	4.7

Table S15. Overview of DRINCZ and MINCZ experiments that were used to simulate size-dependent ice-nucleating particle fractions. Crosses mark suspension concentrations that were discarded.

Experiment	Concentration	Nominal cut sizes of impactor stages (D50)						
		0.32μm	0.56μm	1.0μm	1.8μm	3.2μm	5.6μm	10μm
DRINCZ	0.05wt%							
	0.1wt%							
MINCZ	0.05wt%		X	X	X	X	X	X
	0.1wt%			X	X			
	0.5wt%				X			
	1.0wt%							

Table S16. Particle count with and without a clear edge–basal distinction for each size class. Fractions indicate the proportion of particles lacking a clear distinction relative to the total particles in the corresponding size class.

Size class *i*	Size range (µm)	Particles with clear edge–basal distinction	Particles without clear edge–basal distinction	Fraction of particles without clear distinction
1	0–1	36	0	0
2	1–2	74	0	0
3	2–3	49	0	0
4	3–4	25	0	0
5	4–5	11	0	0
6	5–6	6	0	0
7	6–7	7	0	0
8	7–9	5	0	0
9	9–12	6	1	14.3%
10	12–48	36	24	40.0%

REFERENCES

1. T. Chen, W. B. Rossow, Y. Zhang, Radiative effects of cloud-type variations. *J. Climate* **13**, 264–286 (2000).

2. P. R. Field, A. J. Heymsfield, Importance of snow to global precipitation. *Geophys. Res. Lett.* **42**, 9512–9520 (2015).

3. U. Lohmann, J. Feichter, Global indirect aerosol effects: A review. *Atmos. Chem. Phys.* **5**, 715–737 (2005).

4. J. Mülmenstädt, O. Sourdeval, J. Delanoë, J. Quaas, Frequency of occurrence of rain from liquid-, mixed-, and ice-phase clouds derived from A-Train satellite retrievals. *Geophys. Res. Lett.* **42**, 6502–6509 (2015).

5. U. Lohmann, Aerosol effects on clouds and climate. *Space Sci. Rev.* **125**, 129–137 (2007).

6. R. R. Rogers, M. K. Yau, A short course in cloud physics. *Bull. Am. Meteorol. Soc.* **70**, 1159a (1989).

7. B. J. Murray, K. S. Carslaw, P. R. Field, Opinion: Cloud-phase climate feedback and the importance of ice-nucleating particles. *Atmos. Chem. Phys.* **21**, 665–679 (2021).

8. A. J. Heymsfield, M. Krämer, A. Luebke, P. Brown, D. J. Cziczo, C. Franklin, P. Lawson, U. Lohmann, G. M. Farquhar, Z. Ulanowski, K. Van Tricht, Cirrus Clouds. *Meteorol. Monogr.* **58**, 2.1–2.26 (2017).

9. G. de Boer, T. Hashino, G. J. Tripoli, Ice nucleation through immersion freezing in mixed-phase stratiform clouds: Theory and numerical simulations. *Atmos. Res.* **96**, 315–324 (2010).

10. C. D. Westbrook, A. J. Illingworth, The formation of ice in a long-lived supercooled layer cloud. *Q. J. Roy. Meteorol. Soc.* **139**, 2209–2221 (2013).

11. J. F. Kok, T. Storelvmo, V. A. Karydis, A. A. Adebiyi, N. M. Mahowald, A. T. Evan, C. He, D. M. Leung, Mineral dust aerosol impacts on global climate and climate change. *Nat. Rev. Earth Environ.* **4**, 71–86 (2023).

12. Z. A. Kanji, L. A. Ladino, H. Wex, Y. Boose, M. Burkert-Kohn, D. J. Cziczo, M. Krämer, Overview of ice nucleating particles. *Meteorol. Monogr.* **58**, 1.1–1.33 (2017).

13. B. J. Murray, D. O'Sullivan, J. D. Atkinson, M. E. Webb, Ice nucleation by particles immersed in supercooled cloud droplets. *Chem. Soc. Rev.* **41**, 6519–6554 (2012).

14. J. D. Atkinson, B. J. Murray, M. T. Woodhouse, T. F. Whale, K. J. Baustian, K. S. Carslaw, S. Dobbie, D. O'Sullivan, T. L. Malkin, The importance of feldspar for ice nucleation by mineral dust in mixed-phase clouds. *Nature* **498**, 355–358 (2013).

15. L. Kaufmann, C. Marcolli, J. Hofer, V. Pinti, C. R. Hoyle, T. Peter, Ice nucleation efficiency of natural dust samples in the immersion mode. *Atmos. Chem. Phys.* **16**, 11177–11206 (2016).

16. D. A. Knopf, P. A. Alpert, Atmospheric ice *nucleation*. *Nat. Rev. Phys.* **5**, 203–217 (2023).

17. B. J. Murray, S. L. Broadley, T. W. Wilson, J. D. Atkinson, R. H. Wills, Heterogeneous freezing of water droplets containing kaolinite particles. *Atmos. Chem. Phys.* **11**, 4191–4207 (2011).

18. H. R. Pruppacher, J. D. Klett, P. K. Wang, Microphysics of clouds and precipitation. *Aerosol Sci. Tech.* **28**, 381–382 (1998).

19. M. A. Holden, J. M. Campbell, F. C. Meldrum, B. J. Murray, H. K. Christenson, Active sites for ice nucleation differ depending on nucleation mode. *Proc. Natl. Acad. Sci. U.S.A.* **118**, e2022859118 (2021).

20. M. Niemand, O. Möhler, B. Vogel, H. Vogel, C. Hoose, P. Connolly, H. Klein, H. Bingemer, P. DeMott, J. Skrotzki, T. Leisner, A particle-surface-area-based parameterization of immersion freezing on desert dust particles. *J. Atmos. Sci.* **69**, 3077–3092 (2012).

21. F. Lüönd, O. Stetzer, A. Welti, U. Lohmann, Experimental study on the ice nucleation ability of size-selected kaolinite particles in the immersion mode. *J. Geophys. Res. Atmos.* **115**, D14201 (2010).

22. A. Welti, U. Lohmann, Z. A. Kanji, Ice nucleation properties of K-feldspar polymorphs and plagioclase feldspars. *Atmos. Chem. Phys.* **19**, 10901–10918 (2019).

23. J. Chen, Z. Wu, J. Chen, N. Reicher, X. Fang, Y. Rudich, M. Hu, Size-resolved atmospheric ice-nucleating particles during East Asian dust events. *Atmos. Chem. Phys.* **21**, 3491–3506 (2021).

24. J. Chen, Z. Wu, X. Gong, Y. Qiu, S. Chen, L. Zeng, M. Hu, Anthropogenic dust as a significant source of ice-nucleating particles in the urban environment. *Earth's Future* **12**, e2023EF003738 (2024).

25. N. Reicher, C. Budke, L. Eickhoff, S. Raveh-Rubin, I. Kaplan-Ashiri, T. Koop, Y. Rudich, Size-dependent ice nucleation by airborne particles during dust events in the eastern Mediterranean. *Atmos. Chem. Phys.* **19**, 11143–11158 (2019).

26. N. Hiranuma, S. Augustin-Bauditz, H. Bingemer, C. Budke, J. Curtius, A. Danielczok, K. Diehl, K. Dreischmeier, M. Ebert, F. Frank, N. Hoffmann, K. Kandler, A. Kiselev, T. Koop, T. Leisner, O. Möhler, B. Nillius, A. Peckhaus, D. Rose, S. Weinbruch, H. Wex, Y. Boose, P. J. DeMott, J. D. Hader, T. C. J. Hill, Z. A. Kanji, G. Kulkarni, E. J. T. Levin, C. S. McCluskey, M. Murakami, B. J. Murray, D. Niedermeier, M. D. Petters, D. O'Sullivan, A. Saito, G. P. Schill, T. Tajiri, M. A. Tolbert, A. Welti, T. F. Whale, T. P. Wright, K. Yamashita, A comprehensive laboratory study on the immersion freezing behavior of illite NX particles: A comparison of 17 ice nucleation measurement techniques. *Atmos. Chem. Phys.* **15**, 2489–2518 (2015).

27. Y. Bereznitski, M. Jaroniec, P. Maurice, Adsorption characterization of two Clay Minerals Society standard kaolinites. *J. Colloid Interface Sci.* **205**, 528–530 (1998).

28. S. J. Chipera, D. L. Bish, Baseline studies of the clay minerals society source clays: Powder x-ray diffraction analyses. *Clays Clay Miner.* **49**, 398–409 (2001).

29. K. Klumpp, C. Marcolli, A. Alonso-Hellweg, C. H. Dreimol, T. Peter, Comparing the ice nucleation properties of the kaolin minerals kaolinite and halloysite. *Atmos. Chem. Phys.* **23**, 1579–1598 (2023).

30. G. C. Sosso, T. Li, D. Donadio, G. A. Tribello, A. Michaelides, microscopic mechanism and kinetics of ice formation at complex interfaces: Zooming in on kaolinite. *J. Phys. Chem. Lett.* **7**, 2350–2355 (2016).

31. A. Welti, F. Lüönd, Z. A. Kanji, O. Stetzer, U. Lohmann, Time dependence of immersion freezing: An experimental study on size selected kaolinite particles. *Atmos. Chem. Phys.* **12**, 9893–9907 (2012).

32. H. Wex, P. J. DeMott, Y. Tobo, S. Hartmann, M. Rösch, T. Clauss, L. Tomsche, D. Niedermeier, F. Stratmann, Kaolinite particles as ice nuclei: Learning from the use of different kaolinite samples and different coatings. *Atmos. Chem. Phys.* **14**, 5529–5546 (2014).

33. S. A. Zielke, A. K. Bertram, G. N. Patey, Simulations of Ice Nucleation by Kaolinite (001) with Rigid and Flexible Surfaces. *J. Phys. Chem. B* **120**, 1726–1734 (2016).

34. G. Brown, *Crystal Structures of Clay Minerals and Their X-Ray Identification* (The Mineralogical Society of Great Britain and Ireland, 1982), vol. 5.

35. J. W. Gruner, The crystal structure of kaolinite. *Z. Kristallogr. - Cryst. Mater.* **83**, 75–88 (1932).

36. C. T. Johnston, Probing the nanoscale architecture of clay minerals. *Clay Miner.* **45**, 245–279 (2010).

37. M. Zbik, R. S. C. Smart, Nanomorphology of kaolinites: Comparative SEM and AFM Studies. *Clays Clay Miner.* **46**, 153–160 (1998).

38. E. Galicia-Andres, D. Petrov, M. H. Gerzabek, C. Oostenbrink, D. Tunega, Polarization effects in simulations of kaolinite-water interfaces. *Langmuir* **35**, 15086–15099 (2019).

39. N. Kumar, M. P. Andersson, D. van den Ende, F. Mugele, I. Siretanu, Probing the surface charge on the basal planes of kaolinite particles with high-resolution atomic force microscopy. *Langmuir* **33**, 14226–14237 (2017).

40. B. Wang, D. A. Knopf, S. China, B. W. Arey, T. H. Harder, M. K. Gilles, A. Laskin, Direct observation of ice nucleation events on individual atmospheric particles. *Phys. Chem. Chem. Phys.* **18**, 29721–29731 (2016).

41. T. F. Whale, B. J. Murray, D. O'Sullivan, T. W. Wilson, N. S. Umo, K. J. Baustian, J. D. Atkinson, D. A. Workneh, G. J. Morris, A technique for quantifying heterogeneous ice nucleation in microlitre supercooled water droplets. *Atmos. Meas. Tech.* **8**, 2437–2447 (2015).

42. Y. Ren, A. K. Bertram, G. N. Patey, Effects of inorganic ions on ice nucleation by the Al surface of kaolinite immersed in water. *J. Phys. Chem. B* **124**, 4605–4618 (2020).

43. L. Ickes, A. Welti, C. Hoose, U. Lohmann, Classical nucleation theory of homogeneous freezing of water: Thermodynamic and kinetic parameters. *Phys. Chem. Chem. Phys.* **17**, 5514–5537 (2015).

44. U. Lohmann, F. Lüönd, F. Mahrt, *An Introduction to Clouds: From the Microscale to Climate* (Cambridge Univ. Press, 2016); doi:10.1017/CBO9781139087513.

45. A. Kumar, K. Klumpp, C. Barak, G. Rytwo, M. Plötze, T. Peter, C. Marcolli, Ice nucleation by smectites: The role of the edges. *Atmos. Chem. Phys.* **23**, 4881–4902 (2023).

46. A. Kumar, C. Marcolli, T. Peter, Ice nucleation activity of silicates and aluminosilicates in pure water and aqueous solutions—Part 3: Aluminosilicates. *Atmos. Chem. Phys.* **19**, 6059–6084 (2019).

47. I. de Almeida Ribeiro, Y. Qiu, E. D. Gadea, A. K. Metya, V. Molinero, Immersion freezing at topographic active sites: Dual-barrier prediction of ice nucleation temperatures. *J. Am. Chem. Soc.* **148**, 3291–3304 (2026).

48. A. J. Page, R. P. Sear, Heterogeneous nucleation in and out of pores. *Phys. Rev. Lett.* **97**, 065701 (2006).

49. J. M. Campbell, H. K. Christenson, Nucleation- and emergence-limited growth of ice from pores. *Phys. Rev. Lett.* **120**, 165701 (2018).

50. R. O. David, C. Marcolli, J. Fahrni, Y. Qiu, Y. A. Perez Sirkin, V. Molinero, F. Mahrt, D. Bruhwiler, U. Lohmann, Z. A. Kanji, Pore condensation and freezing is responsible for ice formation below water saturation for porous particles. *Proc. Natl. Acad. Sci. U.S.A.* **116**, 8184–8189 (2019).

51. C. Marcolli, Technical note: Fundamental aspects of ice nucleation via pore condensation and freezing including Laplace pressure and growth into macroscopic ice. *Atmos. Chem. Phys.* **20**, 3209–3230 (2020).

52. K. Toshihiro, Stacking disorder in kaolinite revealed by HRTEM: A review. *Clay Sci.* **15**, 3–11 (2011).

53. R. Kuehn, R. Kilian, D. Lang, L. G. Morales, O. G. Grendal, M. Stipp, Clay alignment takes place during early stages of sedimentation. *Commun. Earth Environ.* **5**, 696 (2024).

54. P. Dagsson-Waldhauserova, J. B. Renard, H. Olafsson, D. Vignelles, G. Berthet, N. Verdier, V. Duverger, Vertical distribution of aerosols in dust storms during the Arctic winter. *Sci. Rep.* **9**, 16122 (2019).

55. A. Adebiyi, J. F. Kok, B. J. Murray, C. L. Ryder, J.-B. W. Stuut, R. A. Kahn, P. Knippertz, P. Formenti, N. M. Mahowald, C. Pérez García-Pando, M. Klose, A. Ansmann, B. H. Samset, A. Ito, Y. Balkanski, C. Di Biagio, M. N. Romanias, Y. Huang, J. Meng, A review of coarse mineral dust in the Earth system. *Aeolian Res.* **60**, 100849 (2023).

56. M. Leinen, J. M. Prospero, E. Arnold, M. Blank, Mineralogy of aeolian dust reaching the North Pacific Ocean: 1. Sampling and analysis. Sampling and analysis. *J. Geophys. Res.* **99**, 21017–21023 (1994).

57. K. Kandler, L. Schütz, C. Deutscher, M. Ebert, H. Hofmann, S. Jäckel, R. Jaenicke, P. Knippertz, K. Lieke, A. Massling, A. Petzold, A. Schladitz, B. Weinzierl, A. Wiedensohler, S. Zorn, S. Weinbruch, Size distribution, mass concentration, chemical and mineralogical composition and derived optical parameters of the boundary layer aerosol at Tinfou, Morocco, during SAMUM 2006. *Tellus B: Chem. Phys. Meteorol.* **61**, 32 (2022).

58. H. Beydoun, M. Polen, R. C. Sullivan, Effect of particle surface area on ice active site densities retrieved from droplet freezing spectra. *Atmos. Chem. Phys.* **16**, 13359–13378 (2016).

59. H. Beydoun, M. Polen, R. C. Sullivan, A new multicomponent heterogeneous ice nucleation model and its application to Snomax bacterial particles and a Snomax–illite mineral particle mixture. *Atmos. Chem. Phys.* **17**, 13545–13557 (2017).

60. A. Kumar, C. Marcolli, B. Luo, T. Peter, Ice nucleation activity of silicates and aluminosilicates in pure water and aqueous solutions—Part 1: The K-feldspar microcline. *Atmos. Chem. Phys.* **18**, 7057–7079 (2018).

61. L. G. Jahn, W. D. Fahy, D. B. Williams, R. C. Sullivan, Role of Feldspar and pyroxene minerals in the ice nucleating ability of three volcanic ashes. *ACS Earth Space Chem.* **3**, 626–636 (2019).

62. Y. Qiu, A. Hudait, V. Molinero, How size and aggregation of ice-binding proteins control their ice nucleation efficiency. *J. Am. Chem. Soc.* **141**, 7439–7452 (2019).

63. J. Forbes, A. Bissoyi, L. Eickhoff, N. Reicher, T. Hansen, C. G. Bon, V. K. Walker, T. Koop, Y. Rudich, I. Braslavsky, P. L. Davies, Water-organizing motif continuity is critical for potent ice nucleation protein activity. *Nat. Commun.* **13**, 5019 (2022).

64. G. Renzer, I. de Almeida Ribeiro, H. B. Guo, J. Frohlich-Nowoisky, R. J. Berry, M. Bonn, V. Molinero, K. Meister, Hierarchical assembly and environmental enhancement of bacterial ice nucleators. *Proc. Natl. Acad. Sci. U.S.A.* **121**, e2409283121 (2024).

65. A. Welti, F. Lüönd, O. Stetzer, U. Lohmann, Influence of particle size on the ice nucleating ability of mineral dusts. *Atmos. Chem. Phys.* **9**, 6705–6715 (2009).

66. V. A. Marple, K. L. Rubow, S. M. Behm, A Microorifice Uniform Deposit Impactor (MOUDI): Description, calibration, and use. *Aerosol Sci. Tech.* **14**, 434–446 (1991).

67. F. N. Isenrich, N. Shardt, M. Rösch, J. Nette, S. Stavrakis, C. Marcolli, Z. A. Kanji, A. J. deMello, U. Lohmann, The Microfluidic Ice Nuclei Counter Zürich (MINCZ): A platform for homogeneous and heterogeneous ice *nucleation*. *Atmos. Meas. Tech.* **15**, 5367–5381 (2022).

68. J. Chen, F. M. O. Jakob, A. Voliotis, H. Wu, S. A. Syafira, O. Oghama, N. Shardt, N. Faure, X. Kong, G. McFiggans, Z. A. Kanji, Ice nucleation abilities and chemical characteristics of laboratory-generated and aged biomass burning aerosols. *Environ. Sci. Technol.* **59**, 2575–2586 (2025).

69. R. O. David, M. Cascajo-Castresana, K. P. Brennan, M. Rösch, N. Els, J. Werz, V. Weichlinger, L. S. Boynton, S. Bogler, N. Borduas-Dedekind, C. Marcolli, Z. A. Kanji, Development of the DRoplet Ice Nuclei Counter Zurich (DRINCZ): Validation and application to field-collected snow samples. *Atmos. Meas. Tech.* **12**, 6865–6888 (2019).

70. G. Vali, Revisiting the differential freezing nucleus spectra derived from drop-freezing experiments: Methods of calculation, applications, and confidence limits. *Atmos. Meas. Tech.* **12**, 1219–1231 (2019).

71. O. K. Aromolaran, A. B. Adebayo, Mineralogical, geochemical, and industrial appraisal of kaolinitic claystones in Share area, northcentral Nigeria. *Arab. J. Geosci.* **15**, 789 (2022).

72. S. Brunauer, P. H. Emmett, E. Teller, Adsorption of gases in multimolecular layers. *J. Am. Chem. Soc.* **60**, 309–319 (1938).

73. K. Gao, F. Friebel, C.-W. Zhou, Z. A. Kanji, Enhanced soot particle ice nucleation ability induced by aggregate compaction and densification. *Atmos. Chem. Phys.* **22**, 4985–5016 (2022).

74. N. Hwang, A. R. Barron, BET surface area analysis of nanoparticles (The Connexions Project, 2011), pp. 1–11.

75. T. P. Wright, M. D. Petters, The role of time in heterogeneous freezing nucleation. *J. Geophys. Res. Atmos.* **118**, 3731–3743 (2013).

76. P. A. Alpert, D. A. Knopf, Analysis of isothermal and cooling-rate-dependent immersion freezing by a unifying stochastic ice nucleation model. *Atmos. Chem. Phys.* **16**, 2083–2107 (2016).

77. G. Vali, Interpretation of freezing nucleation experiments: Singular and stochastic; sites and surfaces. *Atmos. Chem. Phys.* **14**, 5271–5294 (2014).

78. S. L. Barr, B. Wyld, J. B. McQuaid, R. R. Neely III, B. J. Murray, Southern Alaska as a source of atmospheric mineral dust and ice-nucleating particles. *Sci. Adv.* **9**, eadg3708 (2023).

79. C. Hoose, O. Möhler, Heterogeneous ice nucleation on atmospheric aerosols: A review of results from laboratory experiments. *Atmos. Chem. Phys.* **12**, 9817–9854 (2012).

80. T. F. Whale, S. L. Barr, T. Surawy-Stepney, Survival analysis for droplet-freezing data: Kaplan–Meier confidence intervals and log-rank tests. *EGUsphere* **2026**, 1–15 (2026).

81. W. D. Fahy, C. R. Shalizi, R. C. Sullivan, A universally applicable method of calculating confidence bands for ice nucleation spectra derived from droplet freezing experiments. *Atmos. Meas. Tech.* **15**, 6819–6836 (2022).

82. R. S. Smith, B. D. Kay, The existence of supercooled liquid water at 150K. *Nature* **398**, 788–791 (1999).

83. B. Zobrist, T. Koop, B. P. Luo, C. Marcolli, T. Peter, Heterogeneous ice nucleation rate coefficient of water droplets coated by a nonadecanol monolayer. *J. Phys. Chem. C* **111**, 2149–2155 (2007).

84. P. W. Atkins, J. de Paula, J. Keeler, *Atkins' Physical Chemistry* (Oxford Univ. Press, ed. 12, 2023).

85. J. H. Seinfeld, S. N. Pandis, *Atmospheric Chemistry and Physics: From Air Pollution to Climate Change* (John Wiley & Sons, 1998).

86. A. Reinhardt, J. P. Doye, Note: Homogeneous TIP4P/2005 ice nucleation at low supercooling. *J. Chem. Phys.* **139**, 096102 (2013).

87. D. M. Murphy, T. Koop, Review of the vapour pressures of ice and supercooled water for atmospheric applications. *Q. J. Roy. Meteorol. Soc.* **131**, 1539–1565 (2005).

88. L. Kaufmann, C. Marcolli, B. Luo, T. Peter, Refreeze experiments with water droplets containing different types of ice nuclei interpreted by classical nucleation theory. *Atmos. Chem. Phys.* **17**, 3525–3552 (2017).
